# Azidothymidine “Clicked” into 1,2,3-Triazoles: First Report on Carbonic Anhydrase–Telomerase Dual-Hybrid Inhibitors

**DOI:** 10.1021/acs.jmedchem.0c00636

**Published:** 2020-05-28

**Authors:** Emanuela Berrino, Andrea Angeli, Dmitry D. Zhdanov, Anna P. Kiryukhina, Andrea Milaneschi, Alessandro De Luca, Murat Bozdag, Simone Carradori, Silvia Selleri, Gianluca Bartolucci, Thomas S. Peat, Marta Ferraroni, Claudiu T. Supuran, Fabrizio Carta

**Affiliations:** † NEUROFARBA Department, Sezione di Scienze Farmaceutiche e Nutraceutiche, Università degli Studi di Firenze, Via Ugo Schiff 6, 50019 Sesto Fiorentino (Florence), Italy; ‡ Institute of Biomedical Chemistry, Pogodinskaya st. 10/8, 119121 Moscow, Russia; § Peoples Friendship University of Russia (RUDN University), Miklukho-Maklaya st. 6, 117198 Moscow, Russia; ∥ Department of Pharmacy, “G. d’Annunzio” University of Chieti-Pescara, Via dei Vestini 31, 66100 Chieti, Italy; ⊥ CSIRO, 343 Royal Parade, Parkville, Victoria 3052, Australia; # Dipartimento di Chimica “Ugo Schiff”, Università di Firenze, Via della Lastruccia 3-13, 50019 Sesto Fiorentino (Florence), Italy

## Abstract

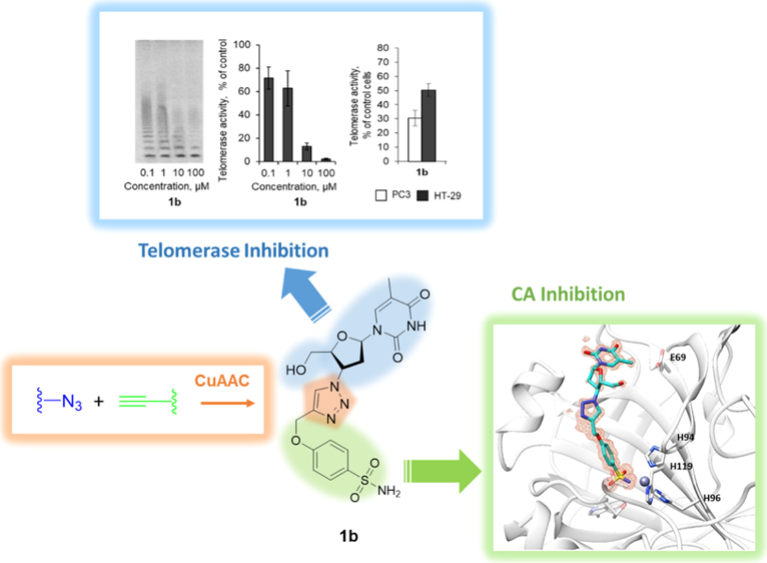

Cancer cells rely on the enzyme telomerase (EC 2.7.7.49) to promote cellular immortality. Telomerase inhibitors (i.e., azidothymidine) can represent promising antitumor agents, although showing high toxicity when administered alone. Better outcomes were observed within a multipharmacological approach instead. In this context, we exploited the validated antitumor targets carbonic anhydrases (CAs; EC 4.2.1.1) IX and XII to attain the first proof of concept on CA–telomerase dual-hybrid inhibitors. Compounds **1b**, **7b**, **8b**, and **11b** showed good in vitro inhibition potency against the CAs IX and XII, with *K*_I_ values in the low nanomolar range, and strong antitelomerase activity in PC-3 and HT-29 cells (IC_50_ values ranging from 5.2 to 9.1 μM). High-resolution X-ray crystallography on selected derivatives in the adduct with hCA II as a model study allowed to determine their binding modes and thus to set the structural determinants necessary for further development of compounds selectively targeting the tumoral cells.

## Introduction

Eukaryotic cells do possess limited replicative potential as progressive shortage of the chromosome ends (i.e., the telomeres) takes place after every duplication cycle.^1^ Once the critical physical limits are reached, cellular senescence programs, that is, apoptosis, are triggered.^2^ Such an effect is properly referred as the “Hayflick limit”, who first reported experimentally the finite capacity of normal cells to replicate.^1,3^

The state-of-the-art knowledge on telomeres accounts for rather complicated and highly dynamic structures which are evolutionarily conserved among the eukaryotic cells.^4^ Human telomeres are composed of repetitive, noncoding hexameric nucleotide repeats in complex with the telomere-associated proteins (i.e., the shelterin proteins) and the telomerase.^5−8^ The former are mainly responsible for maintaining the telomere structure and its signaling functions, whereas the latter for synthetizing new telomeric DNA strands from its own RNA template.^4,5^ This enzyme is normally highly active in adult germ line and stem cells, whereas it is poorly or not expressed at all in the somatic ones.^9,10^ Besides the canonical function of telomere elongation, the telomerase enzymes (EC 2.7.7.49) were also found to act as transcriptional regulators of the Wnt/β-catenin signaling pathway, thus suggesting a role in determining cell growth, differentiation, and apoptosis via a nontelomer-dependent manner.^11−13^

The majority of malignant tumors in humans were demonstrated to depend on the telomerase activity, which resulted in increased telomerase activity when compared to the nontumorigenic counterpart cells.^14^ As a matter of fact, the catalytic subunit of the telomerase enzyme (i.e., hTERT) was found overexpressed in several tumors,^15−18^ and its regulatory role in metastatic events was also proved.^19^ In light of such data, the telomerase is properly considered a tumor marker,^20^ and still it is taken into consideration as a rational target for developing potent and effective anticancer drugs.^15,20−22^

By making use of the DNA polymerase activity of the telomerase, nucleoside and nucleotide analogues have been extensively investigated as potential inhibitors.^23^ In particular, chain-terminator reverse-transcriptase inhibitors have been explored as antitumor agents.^23^ The first study of this type was conducted by Blackburn in 1994 on the ciliated protozoan *Tetrahymena thermophila* which is quite rich in telomeres.^24^ Such studies revealed that azidothymidine (**AZT**) was able to decrease the de novo telomere addition, thus resulting in shortening of telomeres.^24^ Further studies showed that in spite of the low affinity of **AZT** for mammalian DNA polymerases, its triphosphate derivative (**AZT-TP**) was incorporated into the telomeric region of an eukaryotic genome through a process mediated by the telomerases.^25,26^ The efficiency of **AZT** in affecting tumor growth was properly assessed,^27−29^ and its association with cisplatin, paclitaxel, or 5-fluorouracil showed synergistic interactions.^30,31^ Although such promising results were obtained, **AZT** was dropped as an antitumor drug because of its potential tumorigenic properties and the tardiness of the drug to be fully functional, which may expose patients to dangerous side effects.^32^ Various drawbacks are associated with the use of telomerase inhibitors for cancer therapy.^33^ The tardiness to take action is the most critical issue, as cellular senescence is induced only when telomeres have reached their critical length and thus implying that such agents do require appropriate time to become effective.^32,33^ Induction of cellular senescence by telomeric dysfunction may also result in activation of oncogenes and/or silencing of tumor suppressor genes, thus promoting malignant transformations to occur instead.^34^ In addition, the use of inhibitors of the telomerases may interfere with highly proliferative cells such germ lines and stem cells.^10,22^ For all these reasons, the use of telomerase inhibitors (i.e., **AZT**, Imetelstat, BIBR1532, and antisense molecules) for the management of cancer is better envisaged within a polypharmacologically based approach, and the metalloenzyme carbonic anhydrase (CA; EC 4.2.1.1) IX is well suited.^35−37^ CA IX (and marginally CA XII) is selectively overexpressed in hypoxic solid tumors, and it actively participates in a complex pH regulation machinery tuned to warrant cancer cell survival within a metabolically driven pH-dysregulated environment.^37−40^ The paramount importance of CA IX in regulating proton dynamics by means of eq 1 was conclusively demonstrated, which allowed to validate such an enzyme as a druggable target for the management of hypoxic tumors.^38,39^
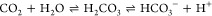
1

A recent contribution on the active involvement of CA IX in tumor physiology demonstrated such an enzyme to provide the H^+^ ions needed by the matrix metalloproteinase 14 to perform proteolytic cleavage of collagen, which in turn determines tumor invasiveness.^41^ In this context, during the last years, great interests have been turned to the CA IX “interactome”.^42−45^ A significant study conducted on HEK-293 cells showed that the ARM and/or HEAT-repeat domains are a feature of CA IX interacting partners.^45^ The majority of such proteins belong to the nuclear-cytoplasmic trafficking machinery, such as XPO1 exportin and TNPO 1 importin, and were found to interact with the CA IX C terminal region.^45^ These results strongly suggested that CA IX may play the role of a cell–surface signal transducer by undergoing nuclear translocation. This is in agreement with confocal immunofluorescence spectroscopy experiments, which showed nuclear distribution of CA IX in several cell lines, with a marked localization when experimental hypoxic conditions were established.^45^

In consideration of the robust antitumor effects observed when the telomerase and the CA IX were targeted, the research herein reported is aimed to obtain CA–telomerase dual small-molecule inhibitors (**CAI–TI**) that are able to (i) efficiently bind to the CA IX (XII) enzymes which is assumed as a discriminant feature between the tumor and normal cells and (ii) exert their antitumoral activity by inhibition of both the CA IX (or XII) and the telomerase. As a consequence, appropriate **CAI–TI** molecules will have the potential to achieve therapeutic performances far superior to the ones reached when coadministration of single therapeutic agents is considered. To the best of our knowledge, this is the first report on **CAI–TI**; dual-hybrid compounds designed to target two crucial players in cancer progression.

## Results and Discussion

### Design and Synthesis of Compounds

**Figure 1:**
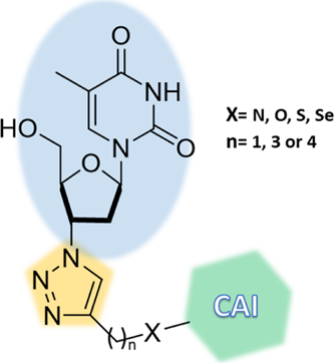
Schematic representation of the synthetized hybrids consisting of a CAI portion linked to **AZT** through the 1,2,3-triazole ring.

The hybridization strategy was performed by exploiting the versatile “click chemistry” approach, which allows to merge efficiently single chemical entities and thus grant easy access to wide molecular diversities.^46,47^ In this study, we performed a copper-catalyzed azide–alkyne cycloaddition (CuAAC) between the azide of the reverse-transcriptase inhibitor **AZT** with the terminal alkyne pendant installed on various CAI scaffolds (Figure 1). Our interest in establishing such a chemical connection was mainly based on (i) the rapid and regioselective formation of the 1,4-disubstituted-1,2,3-triazole ring under mild reaction conditions^47,48^ and (ii) the 1,2,3-triazole is among the most commonly used scaffolds in medicinal chemistry in the last decade because it is a bioisostere of the amide group and it shows good tolerance to metabolic processes as well as to pH fluctuations.^49,50^ In addition, the abundancy of electrons within the triazole ring allows it to establish H-bonds and π–π stacking interactions with biological targets and thus ensuring additional stabilization of the adducts formed.^49,50^

**Figure 2:**
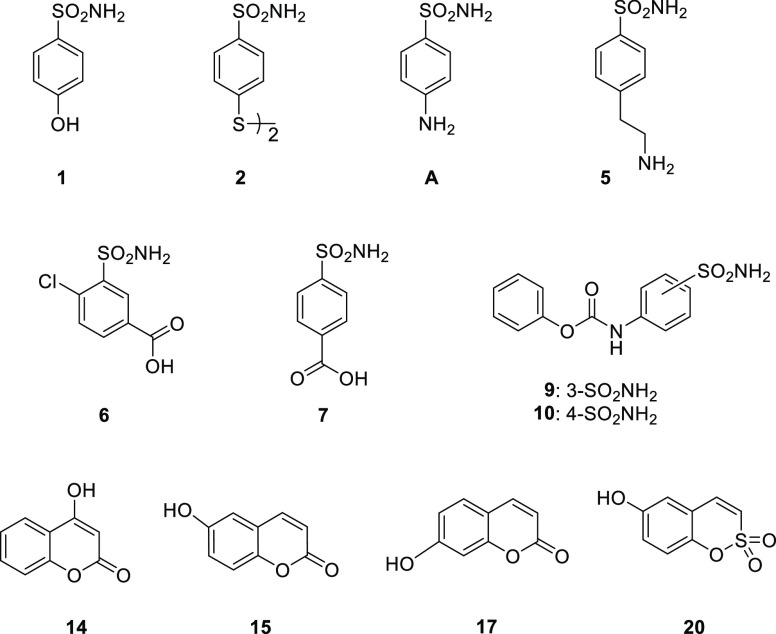
Substrates for the synthesis of alkynes **1b–3b**, **5b–10b**, **14b–20b**, **4d**, and **13e**.

The synthesis and characterization data of the appropriate alkyne precursors **1a–3a**, **5a–10a**, **14a–20a**, and **4c**, reported in Table 1, are descripted within the Experimental Protocols section. Both classical (i.e., sulfonamides) and nonclassical (i.e., coumarins and sulfocoumarins) CAIs have been included in our study. In particular, sulfonamide-based compounds **6a**, **9a**, and **10a** and coumarin-based compounds **14a**, **18a**, and **19a** are new.

**Table 1:**
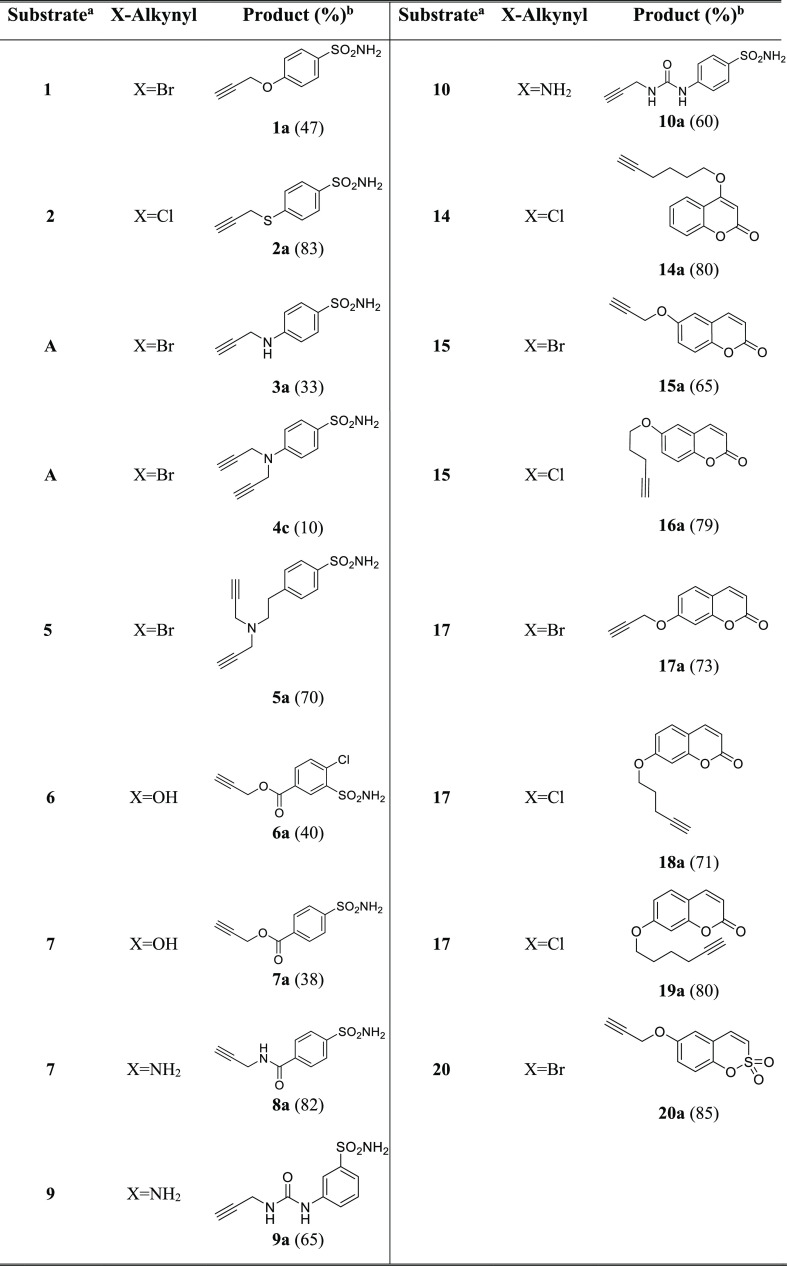
Reagents and Conditions for the Synthesis of Compounds **1a–3a**, **5a–10a**, **14a–20a**, **4c**, and **13d**

a Reported in Figure 2.

b Yields refer to isolated products.

**Scheme 1:**
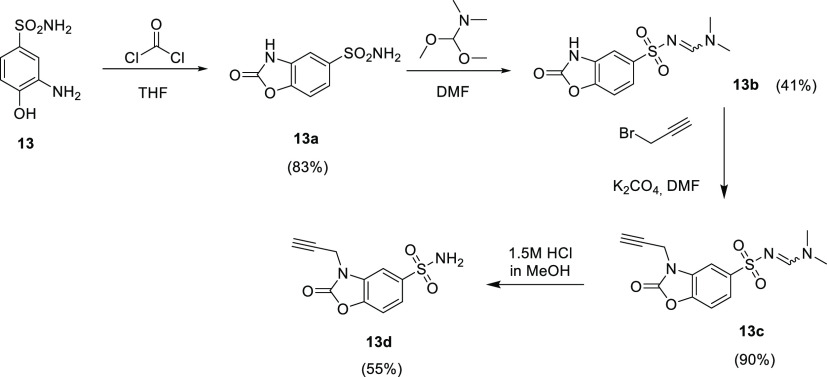
Synthesis of Compounds **1b–3b**, **5b–10b**, **14b–20b**, **4d**, and **13e** Yields are reported in brackets.

Compounds **13d**, here reported for the first time, were obtained through a multistep synthetic approach, reported in Scheme 1.

**Figure 3:**
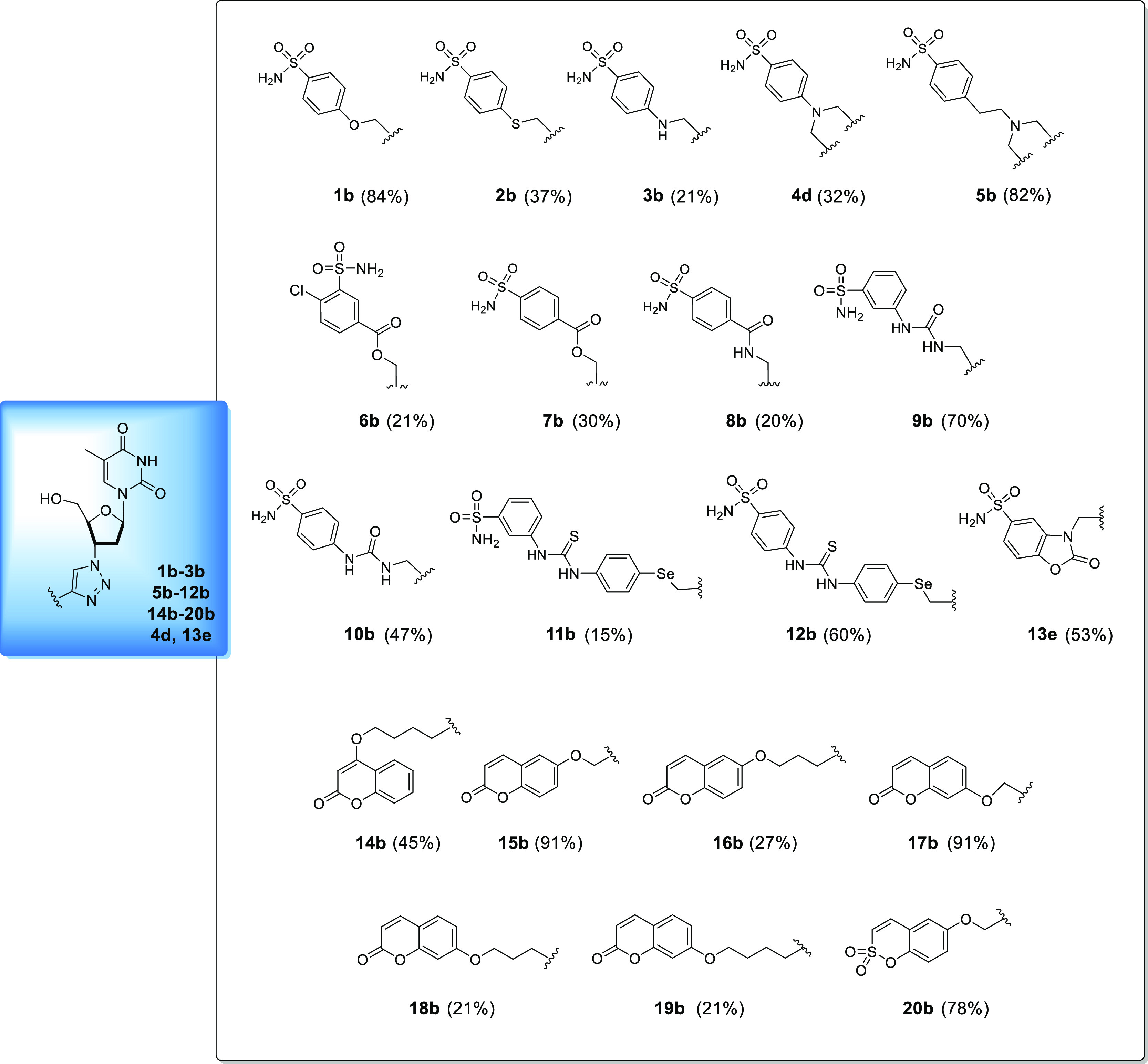
Chemical structures of compounds **1b–3b**, **5b–12b**, **14b–20b**, **4d**, and **13e**. Yields are reported in brackets and are referred to the final coupling reaction.

**Scheme 2:**
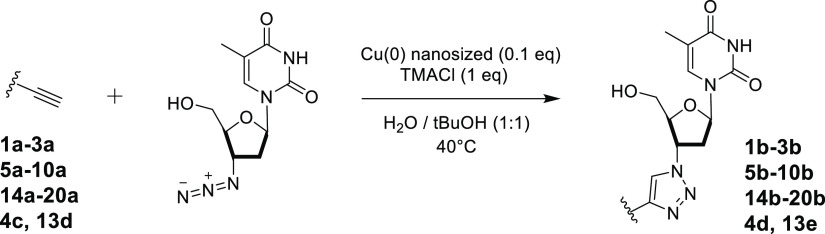
Synthesis of Compounds **1b–3b**, **5b–10b**, **14b–20b**, **4d**, and **13e** Yields are reported in brackets.

The final compounds **1b–3b**, **5b–12b**, **14b–20b**, **4d**, and **13e**, reported in Figure 3, were obtained by performing CuAAC by using Cu (0) nanosized, tetramethyl ammonium chloride (TMACl) as a phase-transfer agent in *t*BuOH/H_2_O 1:1 as a solvent at 40 °C (Scheme 2).

**Scheme 3:**
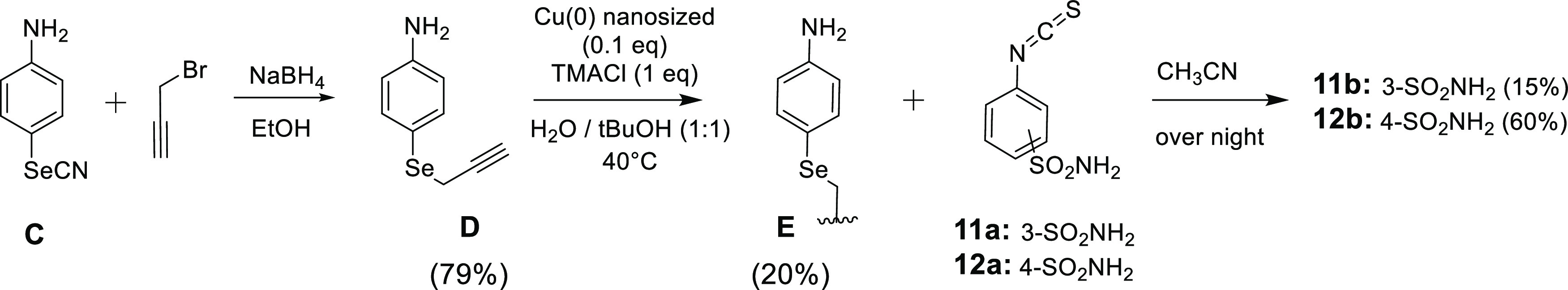
Synthesis of Compounds **11b** and **12b** Yields are reported in brackets.

The syntheses of compounds **11b** and **12b** are reported separately (Scheme 3), as for these compounds, CuAAC was not performed as the last reaction step. The synthesis started with the preparation of compound **D**, bearing the terminal alkyne pendant, obtained by reducing 4-selenocyanatoaniline **C** with NaBH_4_ and treating it in situ with propargyl bromide. The CuAAC reaction between the azide of **AZT** and the terminal alkyne of **D** was then performed to afford the common intermediate **E**, which was subsequently reacted with 3-isothiocyanatobenzenesulfonamide or 4-isothiocyanatobenzenesulfonamide to afford compounds **11b** and **12b**, respectively. All final compounds were obtained in good yields and with high-purity grade (i.e., ≥95%) as determined by high-performance liquid chromatography (HPLC). The structural characterization of both intermediates and final compounds was assessed by means of ^1^H NMR and ^13^C NMR as well as high-resolution mass spectroscopy (HRMS).

To the best of our knowledge, the **AZT**–coumarin derivative **17b** was previously reported in the literature as part of a set of compounds intended to be used for their fluorescent properties. No biological applications were reported in such a study.^51^ In addition, ester-triazole-linked triterpenoid–**AZT** conjugates were also reported.^52^ Cytotoxic analysis of these hybrids and their triterpenoid precursors revealed moderate to good cytotoxic activities against two human tumor cell lines (KB and Hep-G2).^52^ However, no detailed studies on the specific targets responsible for the anticancer effects were conducted.

### CA Inhibition Profiling

The library of compounds obtained, **1b–3b**, **5b–12b**, **14b–20b**, **4d**, and **13e**, was evaluated for the inhibition properties against the human-expressed (h) CAs I, II, VA, VB, VII, IX, and XII isoforms by means of the stopped-flow technique applied to the CO_2_ hydrase assay.^53^ The inhibition data compared to those of the standard sulfonamide inhibitor acetazolamide (**AAZ**) are reported in Table 2.

**Table 2:** Inhibition Data of hCA I, hCA II, hCA VA, hCA VB, hCA VII, hCA IX, and hCA XII with Compounds **1b–3b**, **5b–12b**, **14b–20b**, **4d**, and **13e** and the Standard Sulfonamide Inhibitor **AAZ** by a Stopped-Flow CO_2_ Hydrase Assay^53^

*K*_I_ (nM)^a^							
	hCA I	hCA II	hCA VA	hCA VB	hCA VII	hCA IX	hCA XII
**1b**	4666.7	9.3	59.1	141.3	51.6	6.2	78.9
**2b**	4037.5	7.7	57.3	52.6	31.0	653.3	61.6
**3b**	>10,000	32.9	64.6	52.6	329.8	488.6	74.4
**4d**	>10,000	8.5	57.3	45.9	383.5	6557.1	74.0
**5b**	>10,000	70.7	59.4	42.9	281.1	8047.1	74.0
**6b**	85.5	7.7	3217.2	22.6	688.5	240.1	40.4
**7b**	28.0	1.3	4795.3	54.2	48.5	3.7	7.0
**8b**	483.0	13.4	1469.3	29.0	9.5	85.5	7.8
**9b**	289.7	6.3	3243.0	47.8	9.4	>10,000	8.4
**10b**	93.1	8.2	437.8	37.9	9.4	267.6	38.9
**11b**	92.8	73.2	3972.2	45.0	66.0	373.2	9.0
**12b**	62.3	5.6	6258.9	46.7	21.8	>10,000	7.1
**13e**	8771.0	21.3	3651.5	28.0	38.0	>10,000	8.1
**14b**	>10,000	>10,000	1725.8	44.0	0.7	>10,000	8.7
**15b**	>10,000	>10,000	57.8	161.0	9.3	6557.1	3.6
**16b**	>10,000	>10,000	666.8	40.1	0.7	21.2	9.4
**17b**	>10,000	>10,000	179.4	151.5	9.4	4885.7	3.5
**18b**	>10,000	>10,000	301.8	42.7	0.6	2948.3	40.4
**19b**	>10,000	>10,000	531.2	43.9	0.6	>10,000	8.9
**20b**	>10,000	>10,000	172.4	54.6	10.5	5852.3	2.8
**AAZ**	250.0	12.1	63.0	54.0	2.5	25.8	5.7

a Mean from three different assays by a stopped-flow technique (errors were in the range of ±5–10% of the reported values).

As reported in Table 2, the compound series was investigated on the most relevant hCA isoforms such as the ubiquitous hCAs I and II, the mitochondria-expressed hCAs VA and VB, the abundantly central nervous system (CNS)-expressed hCA VII, and the tumor-associated hCAs IX and XII.

The structure–activity relationships (SARs) for the titled compounds are discussed below:

i) Overall, the compound series screened in vitro against the ubiquitous hCAs I and II showed preferential inhibition in the low nanomolar range for the latter. In both cases, the coumarin- and sulfocoumarin-based derivatives (i.e., **14b–20b**) resulted ineffective (i.e., *K*_I_s > 10,000 nM) in agreement with the previously reported data.^54,55^ The isosteric ethers **1b** and **2b** resulted in low micromolar inhibitors of hCA I with the latter being just a 1.2-fold more potent inhibitor (*K*_I_s 4666.7 and 4037.5 nM, respectively). Interestingly, the same kinetic profile for both compounds was retained for the hCA II isoform, although the kinetic data were in the low nanomolar range (*K*_I_s 9.3 and 7.7 nM, respectively). Further manipulations on the scaffold of the type reported in compounds **3b**, **4d**, and **5b** resulted detrimental for the hCA I (*K*_I_s > 10,000 nM). As for the isoform II, the introduction of a N atom as in **3b** and **5b** determined enhancement of the *K*_I_ values (32.9 and 70.7 nM, respectively), which were realigned to the previous ones when the *N*,*N*-bis-substituted aniline moiety was introduced instead (*K*_I_**4d** 8.5 nM). Compound **7b** was the most potent inhibitor among the series against both the hCAs I and II (*K*_I_s 28.0 and 1.3 nM, respectively). Variations of the sulfonamide position (i.e., **6b**) or of the linker connection (i.e., **8b**) badly affected the potencies (see Table 2). Noteworthily, the switch of the sulfonamide moiety from 3- to 4-position as in **9b** to **10b** and **11b** to **12b** resulted in a decrease of the inhibition values for hCA I. As for hCA II, a similar profile was observed only for **11b** and **12b**, whereas the opposite was obtained for the regioisomers **9b** and **10b** (i.e., K_I_s 6.3 and 8.2 nM, respectively). Finally, compound **13e** showed excellent discrimination between the isoforms tested, being 411.8-fold more potent against hCA II over hCA I.
ii) Despite the high degree of similarity between the mitochondrially expressed hCAs VA and VB, the kinetic profile of the majority of the tested compounds accounted for the preferential inhibition of the latter. The ether derivative **1b** was the only sulfonamide-bearing compound among the series which showed selective inhibition of hCA VA over VB up to 2.4-fold. The substitution of the ethereal oxygen in **1b** with a sulfur or a nitrogen instead, as in compounds **2b** and **3b**, respectively, suppressed any isoform selectivity, which was maintained when *N*,*N*-disubstitution (i.e., **4d**) or elongation (i.e., **5b**) was applied (see Table 2). As for the remaining sulfonamide derivatives **6b–12b** and **13e**, their *K*_I_ values against hCA VA were all in the micromolar range with compound **10b** being the most potent among them (*K*_I_ 437.8 nM). The same compounds were more effective in inhibiting the second mitochondrially expressed hCA as they showed medium nanomolar *K*_I_ values. The derivatives **6b**, **13e**, and **8b** were the most effective against the hCA VB and their *K*_I_ values resulted up to 2.4-fold lower when compared to the reference **AAZ** (see Table 2). Interesting kinetic data were observed for the coumarin-containing CAIs. The 4-alkyl-substituted derivative **14b** resulted quite effective in inhibiting hCA VB with a selectivity index (SI; *K*_I_ hCA VA/hCA VB) of 39.2. Relocation of the chain to 7-position of the coumarin ring as in **19b** did not change the kinetic profile but heavily reduced the SI for the preferential inhibition of the VB isoform (see Table 2). Regioisomeric effects on kinetics were also evident for compounds **15b** and **17b**. As reported in Table 2, the 6-methylenesubstituted coumarin derivative **15b** resulted a 2.8-fold stronger inhibitor of hCA VA over hCA VB. The preferential inhibition for the former was lost when the chain in **15b** was moved to the adjacent 7-position as in **17b** (see Table 2). Interestingly, the same swapping position as in compounds **16b** and **18b** did not alter the SI, which was in favor of the hCA VB for both derivatives, and affected its intensity as it resulted halved. Finally, the sulfocoumarin prodrug **20b** also reported preferential inhibition for the hCA VB isoform with *K*_I_ values of 172.4 and 54.6 nM, respectively.
iii) As for the CNS-expressed hCA VII, the majority of the compounds tested resulted low nanomolar inhibitors. On considering the SARs, it is worth noting that the ethers **1b** and **2b** showed *K*_I_ values within the medium nanomolar range (51.6 and 31.0 nM, respectively). The introduction of a nitrogen atom instead (i.e., compounds **3b** and **4d**) or a tertiary amine with an alkyl spacer (i.e., compound **5b**) spoiled the inhibition potency against the hCA VII and thus raising the inhibition values up to the high nanomolar range (see Table 2). Interestingly, the ester linkage seems to affect the inhibition potency for this isoform as demonstrated by the kinetic data for both compounds **6b** and **7b**. As a matter of fact, the insertion of the amide, as in compound **8b**, or the ureido linker (i.e., **9b** and **10b**) resulted in a sensible enhancement of the hCA VII inhibition potency as reported in Table 2 for the corresponding *K*_I_ values which are all comprised in the low nanomolar range (i.e., 9.5, 9.42, and 9.4 nM for **8b–10b**, respectively). Interesting results were obtained for the seleno-containing compounds **11b** and **12b** as the regioisomer effect on kinetics was clearly observed. As reported in Table 2, the para-substituted benzenesulfonamide derivative **12b** was a 3.0-fold more potent inhibitor of hCA VII when compared to the meta one **11b** (*K*_I_s 21.8 and 66.0 nM, respectively). Finally, among the sulfonamide-containing CAIs is the 2-oxo-2,3-dihydrobenzo[*d*]oxazole derivative **13e** which resulted in a medium hCA VII nanomolar inhibitor with a *K*_I_ value of 38.0 nM. As for the coumarin-containing CAI moieties, the regioisomeric substitution seems to be ineffective on the kinetic profile of such compounds against the hCA VII isoform. As reported in Table 2, compounds **14b–19b** resulted in low nanomolar inhibitors, and among them, the 6- and 7-methylene-substituted derivatives **15b** and **17b** were the less effective when compared to compounds bearing longer alkyne chain between the CAI portion and the **AZT** scaffold (**14b**, **16b**, **18b**, and **19b**).
iv) A very interesting inhibitory profile can be observed for all the synthetized compounds against the tumor-associated isoforms hCA IX and XII. In general, all of them acted as low nanomolar inhibitors of CA XII, with *K*_I_ values ranging from 2.8 to 78.9 nM. As for CA IX, the different CAI moiety inserted within the scaffold (sulfonamide or coumarin) as well as the substitution patterns both turned out to deeply influence the inhibition potency against this isoform. Three main groups can be delineated on the basis of the observed *K*_I_ values against CA IX. The first group has compounds that efficiently inhibit both tumor-associated isoforms, such as compounds **1b**, **7b**, **8b**, and **16b** (*K*_I_ values < 100 nM against CA IX) and compounds **2b**, **3b**, **6b**, **10b**, and **11b** (*K*_I_ values < 1000 nM against CA IX). Except for compound **16b**, which is a 6-substituted coumarin derivative, all the compounds belonging to this group are sulfonamide-based derivatives, in which only one **AZT** moiety is present within the scaffold. In the second group (*K*_I_ values < 10,000 nM against CA IX), we can include disubstituted sulfonamide-based compounds **4d** and **5b**, in which two **AZT** moieties were “clicked” to the dipropargyl aminobenzensulfonamide and ethylaminobenzenesulfonamide, respectively. In particular, the ethylaminobenzenesulfonamide derivative **5b** proved to be 1.23-fold less potent against CA IX then the shorter analogue **4d** (Table 2). In the second group, we can also enumerate coumarin-based compounds **15b**, **17b**, and **18b** and sulfocoumarin compound **20b**, which inhibited CA IX in the micromolar range (*K*_I_ values ranging from 2948.3 to 6557.1 nM). Interestingly, these compounds strongly inhibited CA XII in the low nanomolar range (Table 2). Finally, in the third group (*K*_I_ values > 10,000 nM against CA IX), we can find compounds which selectively inhibited CA XII over CA IX. In particular, 4- and 7-substituted coumarins **14b** and **19b**, both bearing a four methylene alkyne chain between the coumarin scaffold and the **AZT** moiety, showed to be ineffective against CA IX in the concentration range considered, whereas a strong inhibition of CA XII can be observed (*K*_I_ values of 8.7 and 8.9 nM for **14b** and **19b**, respectively). Meta-substituted ureido compound **9b**, para-substituted thioureido compound **12b**, and 2-oxo-2,3-dihydrobenzo[*d*]oxazole-5-sulfonamide compound **13e** proved to be inactive in CA IX inhibition too (*K*_I_ values > 10,000 nM). Again, a strong CA XII inhibition can be observed for all the compounds. Noteworthily, comparing homologous compounds such as meta- and para-substituted ureido compounds **9b** and **10b** and thioureido compounds **11b** and **12b**, the crucial impact of the regioisomer on the inhibition potency against CA IX can be appreciated, one isomer being about 30-fold more potent than the other. In particular, the meta-substituted ureido compound **10b** proved to be more potent than the para-analogue **9b**, whereas for the seleno-containing thioureido compounds **11b** and **12b**, the meta analogue **11b** showed to be the most potent.

### Cocrystallographic Studies

**Figure 4:**
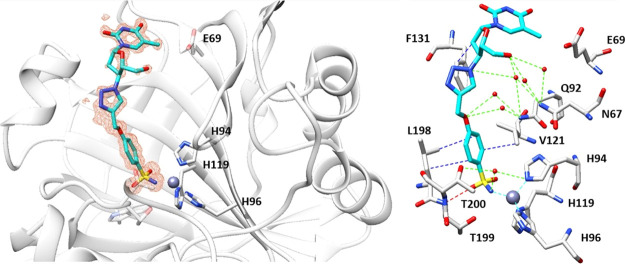
Inhibitor **1b** bound within the active site of hCA II at 1.1 Å resolution and showing the σ*A*-weighted |*F*_o_ – *F*_c_| map contoured at 2.5σ. Ligand **1b** is shown in cyan. Hydrogen bonds, van der Waals interactions, and water bridges are shown and labeled in red, blue, and green, respectively. Residues involved in the binding of inhibitors are also shown. PDB access code 6YPW.

**Figure 5:**
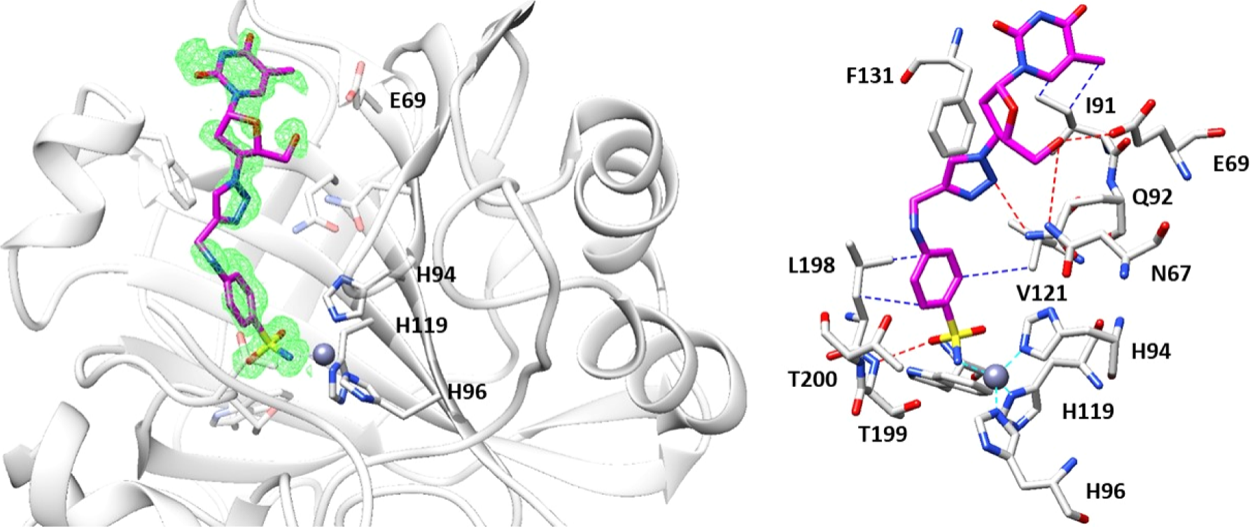
Inhibitor **3b** bound within the active site of hCA II at 1.3 Å resolution and showing the σ*A*-weighted |*F*_o_ – *F*_c_| map contoured at 2.5σ. Ligand **3b** is shown in magenta. Hydrogen bonds and van der Waals interactions are shown and labeled in red and blue. Residues involved in the binding of inhibitors are also shown. PDB access code 6WKA.

In light of the promising *K*_I_ values observed against the tumor-associated isoforms CA IX and XII, the binding modes of compounds **1b** and **3b** within hCA II, used as a model study, were determined by means of X-ray experiments. The electron density maps of both **1b**–hCA II and **3b**–hCA II adducts accounted for both ligands placed well-ordered within the enzymatic cleft with their sulfonamide moieties deep buried up to the bottom of the cavity and coordinated to the zinc(II) ion in the canonical tetrahedral geometry. Again the additional interaction between the sulfonamidic oxygen with the T199 residue was conserved (Figures 4 and 5).^56^

**Figure 6:**
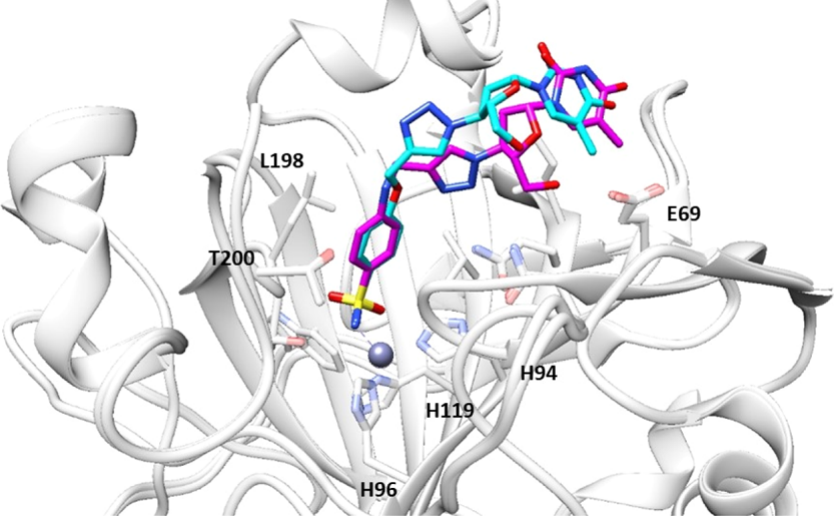
Superposition of inhibitors **1b** and **3b** bound in the active site of hCA II. Ligand **1b** is shown in cyan and **3b** in magenta.

The ligand backbones of **1b** and **3b** are stabilized within the hCA II cavity site by means of a network of hydrogen bonds and van der Waals interactions with substantial differences of the tail orientations as clearly shown when superposition of two structures was performed as shown in Figure 6.

The diverse spatial orientations of the tail sections must be ascribed to the replacement of the ethereal oxygen in **1b** with the nitrogen atom instead as in **3b**, which is the only structural difference among them. The tail in **1b** is located toward the hydrophobic half of the catalytic cleft with the F131 residue acting as the major clipping point. The adduct is further stabilized by a network of hydrogen bonds, which connects the inner face of the inhibitor to the opposite hydrophilic half of the enzymatic cleft by means of bridged water molecules (Figures 4 and 6). As for the compound **3b** tail, it resulted laid toward the hydrophilic section of the enzymatic cavity and directly stabilized by means of hydrogen bonds to the aminoacidic residues N67, E69, and Q92 (Figures 5 and 6). Such results were in agreement with the previously discussed CA kinetic data, which showed the strongly stabilized compound **1b** being a 3.7-fold more potent inhibitor against hCA II when compared to **3b**.

### Telomerase Activity Assay

**Figure 7:**
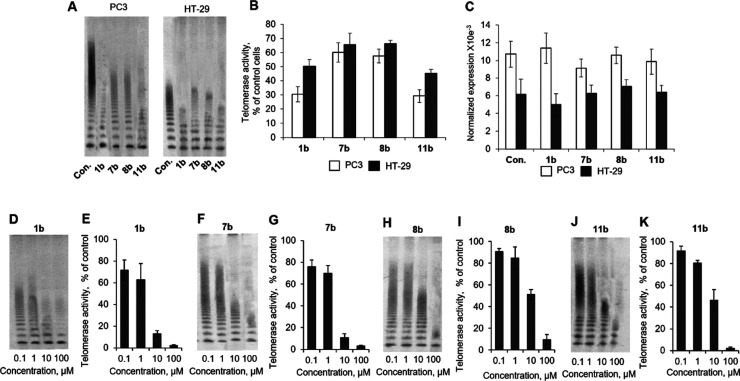
Suppression of telomerase activity by **CAI–TI** compounds. (A) Representative TRAP gel electrophoresis for PC3 or HT-29 cells incubated with 20 μM **CAI–TI** for 48 h. (B) Quantification of TRAP for living cells. (C) hTERT expression in incubated PC3 or HT-29 cells. Levels of hTERT mRNA were normalized relative to the levels of reference 18S RNA. (D,F,H,J) Representative TRAP gel electrophoresis for cell lysates treated with different concentrations of **CAI–TI**. (E,G,I,K) Quantification of TRAP for treated cell lysates. One representative TRAP gel of total four for each of the experiment is shown. The results are presented as the mean ± standard error of the mean. Con., control intact cells.

As mentioned above, **AZT** is known to be a potent telomerase inhibitor.^57,58^ To check whether our compounds can affect telomerase, we incubated PC3 and HT-29 cells with the most potent CA IX and XII **CAI–TI** compounds **1b**, **7b**, **8b**, or **11b** and measured telomerase activity. The results of the telomerase repeated amplification protocol (TRAP) assay showed that all the tested compounds suppressed telomerase in both PC3 and HT-29 cells (Figure 7A,B). The telomerase activity in PC3 cells was higher than in HT-29. Compounds **1b** and **11b** demonstrated the strongest antitelomerase activity, while **7b** and **8b** appeared to be less potent.

Telomerase activity is strongly regulated by the expression of its catalytic subunit hTERT, and inhibition of its expression can be one of the ways of how **CAI–TI** suppresses telomerase in cells.^59^

We investigated hTERT expression in cells incubated with **CAI–TI**. In general, hTERT expression in PC3 cells was higher than in HT-29, which corresponds to increased telomerase activity in such cells (Figure 7C). We found that the compounds have no effect on hTERT gene expression in both types of cells. Another possible way of telomerase inhibition is the binding of substance to hTERT protein subunit.^60^ As it is shown in Figure 7A, PC3 cells have more active telomerase, that is why their lysates were used for telomerase testing in cell-free experiments. All the compounds demonstrated dose-dependent activity to inhibit telomerase within the rage of concentrations 0.1–100 μM (Figure 7D–K). The IC_50_ and IC_90_ values for each compound are shown in Table 3. Compounds **1b** and **11b** had the lowest IC_50_, that is in accordance to telomerase inhibition in living cells.

**Table 3:** Determined IC_50_ and IC_90_ Values for Telomerase Inhibitors (**CAI–TI**)

	IC_50_, μM^a^	IC_90_, μM^a^
**1b**	5.2	40.0
**7b**	6.0	31.8
**8b**	9.1	60.3
**11b**	5.6	42.8

a Mean from four different assays by RTQ-TRAP (errors were in the range of ±5% of the reported values).

## Conclusions

To the best of our knowledge, this work is the proof-of-concept study about the concomitant use of CAIs and TIs merged within the same molecular scaffold and able to act on two validated targets for the management of cancer. Molecular hybridization is a powerful tool in medicinal chemistry with extensive and several successful applications reported so far.^61^ Herein, a series of 20 **CAI–TI** of the **AZT**-type compounds has been synthetized and fully characterized. Then, inhibition potencies against the two designed targets have been assessed. CA inhibition data against seven hCA isoforms revealed that all the titled compounds **1b–3b**, **5b–12b**, **14b–20b**, **4d**, and **13e** strongly inhibit hCA XII, whereas some of them (**1b–3b**, **6b–8b**, **10b**, **11b**, and **16b**) showed medium–high inhibition potency against hCA IX.

The evaluation of telomerase activity in cell lysates or in cells incubated with the CA IX and XII most potent inhibitors **1b**, **7b**, **8b**, and **11b** showed their strong antitelomerase properties, which rely on the ability to suppress processivity of the enzyme rather than the suppression of hTERT expression.

High-resolution X-ray crystallography on compounds **1b** and **3b** in adduct with hCA II as a model study allowed to properly assess their binding mode. In particular, we (i) highlighted the crucial role played by a single heteroatom in determining CA isoform selectivity by means of diverse space orientation of the tail and (ii) first determined the molecular features of the **CAI–TI** molecules, which may be useful to address CA selectivity once proper chemical manipulation is operated.

Overall, the preliminary results obtained in this study fully sustained our strategy and gave us a strong background to further proceed in developing ad hoc designed **CAI–TI** molecules, which will be considered in appropriate tumor cell lines.

## Experimental Protocols

### Chemistry

Anhydrous solvents and all reagents were purchased from Sigma-Aldrich (Milan, Italy), Alfa Aesar (Milan, Italy), and TCI (Milan, Italy). All reactions involving air- or moisture-sensitive compounds were used under a nitrogen atmosphere using dried glassware and syringes to transfer solutions. Nuclear magnetic resonance spectra (^1^H NMR: 400 MHz; ^13^C NMR: 100 MHz) were recorded in DMSO-*d*_6_ using an Avance III 400 MHz spectrometer (Bruker, Milan, Italy). Chemical shifts are reported in parts per million (ppm) and the coupling constants (*J*) are expressed in hertz (Hz). Splitting patterns are designated as follows: s, singlet; d, doublet; t, triplet; q, quadruplet; m, multiplet; br s, broad singlet; and dd, double of doublets. The assignment of exchangeable protons (OH and NH) was confirmed by the addition of D_2_O.

The purity of the final compounds was determined in high-purity grade (i.e., ≥95%) by HPLC using an Agilent 1200 liquid chromatography system composed by an autosampler, binary pumps, a column oven, and a diode-array detector (LC–DAD) operating in UV range (210–400 nm). The operating conditions were reported within the Supporting Information file.

The solvents used in MS measures were acetone and acetonitrile (CHROMASOLV grade), purchased from Sigma-Aldrich, and mQ water 18 MΩ cm, obtained from Millipore’s Simplicity system (Milan, Italy). The HRMS analysis was performed with a Thermo Finnigan LTQ Orbitrap mass spectrometer equipped with an electrospray ionization (ESI) source. The accurate mass measure was carried out by introducing, via a syringe pump at 10 μL min^–1^, the sample solution (1.0 μg mL^–1^ in mQ water/acetonitrile 50:50), and the signal of the positive ions was acquired. The proposed experimental conditions allowed to monitor the protonated molecules of studied compounds ([M + H]^+^ species), such that they were measured with a proper dwell time to achieve 60,000 units of resolution at full width at half-maximum.

### Synthesis of Final Compounds **1b–3b**, **5b–12b**, **14b–20b**, **4d**, and **13e** and Their Intermediates

#### General Procedure A

Proper alkyl halide (1.2 equiv) was added to a suspension of either **1**, **14**, **15**, or **17–20** (0.5 g, 1.0 equiv) and K_2_CO_3_ (2.0 equiv) in dry dimethylformamide (DMF) (4 mL) under a N_2_ atmosphere. The mixture was stirred at room temperature (r.t.) or 60 or 100 °C depending on the alkyl halide until consumption of the starting material [5 h, thin-layer chromatography (TLC) monitoring]. The reaction mixture was cooled at r.t. and quenched with slush. The mixture was extracted with EtOAc (×3), and the combined organic layers were washed with H_2_O and brine solution, dried over Na_2_SO_4_, filtered-off, and then concentrated under vacuum.

#### General Procedure B

The proper carbamate derivative **9** or **10** (0.5 g, 1 equiv) was dissolved in EtOH, and propargylamine (1.2 equiv) was added. The reaction was refluxed for 16 h, then cooled at r.t., and quenched with slush. The mixture was extracted with EtOAc (×3), dried over Na_2_SO_4_, filtered-off, and concentrated under vacuum to afford a solid that was purified by silica gel column chromatography eluting with 60% EtOAc/Hx.

#### General Procedure C

To a suspension of azidonucleoside **AZT** (1.1 equiv or 2.2 equiv) in H_2_O/*t*-BuOH 1:1 (4 mL), the appropriate alkyne (0.12 g, 1.0 equiv) was added at r.t., followed by copper (0) nanosized (0.1 equiv) and TMACl (1.0 equiv). The suspension was stirred at 40 °C until starting materials were consumed (TLC monitoring), then diluted with MeOH (20 mL), and filtered through Celite 521. The solvent was evaporated, affording to a residue that was triturated from EtOAc, to give a white powder.

#### 4-(Prop-2-ynyloxy)benzenesulfonamide (**1a**)

Compound **1a** was synthetized according to the general procedure A using 4-hydroxybenzenesulfonamide **1** and propargyl bromide 80% in toluene at 60 °C. It was purified by silica gel column chromatography eluting with 50% ethyl acetate in *n*-hexane to afford the titled compound **1a** as a white powder. 47% yield; δ_H_ (400 MHz, DMSO-*d*_6_): 3.67 (1H, br s, C*H*), 4.94 (2H, br s, C*H*_2_), 7.17 (2H, d, *J* = 7.2, Ar-*H*), 7.28 (2H, s, exchange with D_2_O, SO_2_N*H*_2_), 8.01 (2H, d, *J* = 7.2, Ar-*H*). Experimental data in agreement with reported data.^62^

#### 4-((1-(2-(Hydroxymethyl)-5-(5-methyl-2,4-dioxo-3,4-dihydropyrimidin-1(2*H*)-yl)tetrahydrofuran-3-yl)-1*H*-1,2,3-triazol-4-yl)methoxy)benzenesulfonamide (**1b**)

Compound **1b** was obtained according to the general procedure A using **1a** as the starting material to afford the title compound 1b as a light yellow solid: 84% yield; δ_H_ (400 MHz, DMSO-*d*_6_): 1.85 (3H, s, C*H*_3_), 2.73 (2H, m, C*H*_2_), 3.70 (2H, m, C*H*_2_), 4.27 (1H, q, *J* = 3.5, C*H*), 5.29 (2H, s, C*H*_2_), 5.34 (1H, br t, exchange with D_2_O, O*H*), 5.45 (1H, m, C*H*), 6.46 (1H, t, *J* = 6.5, C*H*), 7.24 (4H, m, overlapped signals, 2× Ar*H*, exchange with D_2_O, SO_2_N*H*_2_), 7.80 (2H, d, *J* = 8.8, Ar*H*), 7.86 (1H, s, C*H*), 8.50 (1H, s, C*H*), 11.36 (1H, br s, exchange with D_2_O, N*H*); δ_C_ (100 MHz, DMSO-*d*_6_): 13.1, 38.0, 55.2, 60.3, 62.0, 84.5, 85.4, 110.5, 115.6, 125.4, 128.5, 137.1, 137.4, 143.2, 151.3, 161.2, 164.6; ESI-HRMS (*m*/*z*): calcd for [M + H]^+^ ion species C_19_H_23_N_6_O_7_S, 479.1343; found, 479.1336.

#### 4-(Prop-2-ynylthio)benzenesulfonamide (**2a**)

NaBH_4_ (23 mg, 0.60 mmol, 3.0 equiv) was added portionwise to a freshly prepared solution of 4,4′-disulfanediyldibenzenesulfonamide **2** (75 mg, 0.20 mmol, 1.0 equiv) in EtOH (2 mL) at r.t. under a N_2_ atmosphere. After 2 h, propargyl chloride (0.42 mmol, 2.1 equiv) was slowly added, and the reaction mixture was stirred at r.t. for 3 h, until complete consumption of the starting material was observed by TLC. The reaction was quenched by addition of saturated NH_4_Cl aqueous solution (2 mL) and diluted with EtOAc (5 mL). The layers were separated, and the aqueous layer was extracted with EtOAc (2× 5 mL), dried over Na_2_SO_4_, filtered, and concentrated under vacuum. The crude material was purified by silica gel flash chromatography to afford the titled compound **2a** as a white solid. 83% yield; δ_H_ (400 MHz, DMSO-*d*_6_): 3.22 (1H, t, *J* = 2.6, C*H*), 4.02 (2H, d, *J* = 2.6, C*H*_2_), 7.37 (2H, s, exchange with D_2_O, SO_2_N*H*_2_), 7.56 (2H, dd, *J* = 2.0, 6.7, Ar-*H*), 7.79 (2H, dd, *J* = 2.0, 6.7, Ar-*H*). Experimental data in agreement with reported data.^63^

#### 4-(((1-(2-(Hydroxymethyl)-5-(5-methyl-2,4-dioxo-3,4-dihydropyrimidin-1(2*H*)-yl)tetrahydrofuran-3-yl)-1*H*-1,2,3-triazol-4-yl)methyl)thio)benzenesulfonamide (**2b**)

Compound **2b** was obtained according to the general procedure C using **2a** as starting material to afford the title compound **2b** as a white solid: 37% yield; δ_H_ (400 MHz, DMSO-*d*_6_): 1.84 (3H, s, C*H*_3_), 2.69 (2H, m, C*H*_2_), 3.64 (2H, m, C*H*_2_), 4.19 (1H, q, *J* = 3.5, C*H*), 4.45 (2H, s, C*H*_2_), 5.38 (2H, m, overlapped signals, 1× C*H*, exchange with D_2_O, 1× O*H*), 6.43 (1H, t, *J* = 6.5, C*H*), 7.36 (2H, s, exchange with D_2_O, SO_2_N*H*_2_), 7.57 (2H, d, *J* = 8.4, Ar*H*), 7.76 (2H, d, *J* = 8.4, Ar*H*), 7.85 (1H, s, C*H*), 8.29 (1H, s, C*H*), 11.35 (1H, br s, exchange with D_2_O, N*H*); δ_C_ (100 MHz, DMSO-*d*_6_): 13.1, 27.0, 37.9, 60.2, 61.6, 84.8, 85.5, 110.5, 124.2, 127.1, 127.7, 137.1, 141.9, 142.1, 144.1, 151.3, 164.6; ESI-HRMS (*m*/*z*): calcd for [M + H]^+^ ion species C_19_H_23_N_6_O_6_S_2_, 495.1115; found, 495.1118.

#### 4-(Prop-2-ynylamino)benzenesulfonamide (**3a**)

Propargyl bromide 80% in toluene (1.2 equiv) was added to a suspension of sulfanilamide **A** (0.5 g, 1.0 equiv) and pyridine (1.2 equiv) in dry DMF (2 mL) under N_2_ atmosphere and the mixture was stirred at 70 °C (TLC monitoring). The reaction was quenched with H_2_O (10 mL) and extracted with EtOAc (3× 15 mL). The combined organic layers were washed with H_2_O (3× 15 mL) and brine (3× 15 mL), dried over anhydrous Na_2_SO_4_, filtered-off, and concentrated under vacuum to give a solid that was purified by silica gel column chromatography eluting with 50% ethyl acetate in *n*-hexane to afford the desired product **65** as a yellow solid. 33% yield; δ_H_ (400 MHz, DMSO-*d*_6_): 3.13 (1H, t, *J* = 2.4, C*H*), 3.97 (2H, dd, *J* = 2.4, 6.0, C*H*_2_), 6.73 (2H, d, *J* = 8.8, Ar-*H*), 6.76 (1H, br t, exchange with D_2_O, N*H*), 6.98 (2H, br s, exchange with D_2_O, SO_2_N*H*_2_), 7.59 (2H, d, *J* = 8.8, Ar-*H*). Experimental data in agreement with reported data.^64^

#### 4-(((1-(2-(Hydroxymethyl)-5-(5-methyl-2,4-dioxo-3,4-dihydropyrimidin-1(2*H*)-yl)tetrahydrofuran-3-yl)-1*H*-1,2,3-triazol-4-yl)methyl)amino)benzenesulfonamide (**3b**)

Compound **3b** was obtained according to the general procedure C using **3a** as the starting material to afford the title compound **3b** as a white solid. 21% yield; δ_H_ (400 MHz, DMSO-*d*_6_): 1.84 (3H, s, C*H*_3_), 2.69 (2H, m, C*H*_2_), 3.68 (2H, m, C*H*_2_), 4.23 (1H, q, *J* = 3.5, C*H*), 4.40 (2H, d, *J* = 5.7, C*H*_2_), 5.32 (1H, br t, 1H, exchange with D_2_O, O*H*), 5.38 (1H, m, C*H*), 6.45 (1H, t, *J* = 6.5, C*H*), 6.74 (2H, d, *J* = 8.8, 2× Ar-*H*), 6.92 (1H, t, *J* = 5.7, exchange with D_2_O, N*H*), 6.98 (2H, s, exchange with D_2_O, SO_2_N*H*_2_), 7.55 (2H, d, *J* = 8.8, 2× Ar-*H*), 7.84 (1H, s, C*H*), 8.24 (1H, s, C*H*), 11.4 (1H, br s, exchange with D_2_O, N*H*); δ_C_ (100 MHz, DMSO-*d*_6_):12.2, 36.9, 37.9, 59.1, 60.7, 83.8, 84.5, 110.1, 111.2, 122.6, 127.2, 130.4, 136.3, 145.2, 150.4, 150.9, 163.7; ESI-HRMS (*m*/*z*): calcd for [M + H]^+^ ion species C_19_H_24_N_7_O_6_S, 478.1503; found, 478.1508.

#### 4-(Diprop-2-ynylamino)benzenesulfonamide (**4c**)

Sulfanilamide **A** (0,5 g, 1.0 equiv) was dissolved in DMF and the solution was cooled to 0 °C. Then, dimethoxy-*N*,*N*-dimethylmethanamine (1.2 equiv) was added. The solution was stirred at r.t. until consumption of the starting material (2 h). The reaction was quenched with dichloromethane, and the precipitate formed was filtered-off and dried to afford *N*′-((4-aminophenyl)sulfonyl)-*N*,*N*-dimethylformimidamide **4a**, which was used for the next step without further purification. *N*′-((4-Aminophenyl)sulfonyl)-*N*,*N*-dimethylformimidamide **4a** (1.0 equiv) was solubilized in dry DMF and K_2_CO_3_ (3.0 equiv) was added. Then, propargyl bromide 80% in toluene (4.0 equiv) was added, and the mixture was stirred at 80 °C until consumption of the starting material. Then, the reaction was quenched with H_2_O (20 mL) and extracted with EtOAc (3× 15 mL). The combined organic layers were washed with H_2_O (3× 15 mL) and brine(3× 15 mL), dried over Na_2_SO_4_, filtered-off, and concentrated under vacuum to give a residue (**4b**) that was suspended in isopropylamine in a sealed tube and stirred at r.t. The solvent was removed in vacuo, obtaining a residue that was purified by silica gel column chromatography eluting with 50% ethyl acetate in *n*-hexane to afford a sticky residue which was triturated from Et_2_O to afford the titled compound **4c** as a white powder: 10% yield; δ_H_ (400 MHz, DMSO-*d*_6_): 3.21 (2H, t, *J* = 2.4, 2× C*H*), 4.29 (4H, d, *J* = 2.4, 2× C*H*_2_), 7.03 (2H, d, *J* = 8.8, Ar-*H*), 7.08 (2H, s, exchange with D_2_O, SO_2_N*H*_2_), 7.70 (2H, d, *J* = 8.8, Ar-*H*). Experimental data in agreement with reported data.^65^

#### 4-(Bis((1-(2-(hydroxymethyl)-5-(5-methyl-2,4-dioxo-3,4-dihydropyrimidin-1(2*H*)-yl)tetrahydrofuran-3-yl)-1*H*-1,2,3-triazol-4-yl)methyl)amino)benzenesulfonamide (**4d**)

Compound **4d** was obtained according to the general procedure C using **4c** as the starting material to afford the title compound **4d** as a light yellow solid. 32% yield; δ_H_ (400 MHz, DMSO-*d*_6_): 1.84 (6H, s, 2× C*H*_3_), 2.69 (4H, m, 2× C*H*_2_), 3.68 (4H, m, 2× C*H*_2_), 4.21 (2H, q, *J* = 3.8, 2× C*H*), 4.79 (4H, s, 2× C*H*_2_), 5.35 (2H, br t, exchange with D_2_O, 2× O*H*), 5.40 (2H, m, 2× C*H*), 6.45 (2H, t, *J* = 6.4, 2× C*H*), 7.03 (4H, m, overlapped signals, 2× Ar*H*, exchange with D_2_O, SO_2_N*H*_2_), 7.60 (2H, d, *J* = 8.9, 2× Ar*H*), 7.85 (2H, s, 2× C*H*), 8.28 (2H, s, 2× C*H*), 11.38 (2H, br s, exchange with D_2_O, 2× N*H*). δ_C_ (100 MHz, DMSO-*d*_6_): 12.2, 37.9, 45.4, 59.1, 60.7, 83.8, 84.5, 110.1, 111.2, 122.6, 127.2, 130.4, 136.3, 145.2, 150.4, 150.9, 163.7; ESI-HRMS (*m*/*z*): calcd for [M + H]^+^ ion species C_32_H_39_N_12_O_10_S, 783.2627; found, 783.2632.

#### 4-(2-(Di-prop-2-ynylamino)ethyl)benzenesulfonamide (**5a**)

Propargyl bromide (80% in toluene) (2 equiv) and *N*, *N*-diisopropylethylamine (1.7 equiv) were added to a stirred solution of 4-(2-aminoethyl)benzensulfonamide **5** (0.5 g, 1.0 equiv) in CH_3_CN (8 mL) under a N_2_ atmosphere. The mixture was stirred at r.t. until consumption of the starting material (TLC monitoring). The solvent was removed under reduced pressure and the obtained residue was portioned between H_2_O and EtOAc, followed by extraction with EtOAc (3× 15 mL). The combined organic layers were washed with H_2_O (3× 15 mL) and brine (3× 15 mL), then dried over Na_2_SO_4_, filtered-off, and concentrated under vacuum to afford compound **5a** as a dark oil. 70% yield; δ_H_ (400 MHz, DMSO-*d*_6_): 2.75 (2H, t, *J* = 6.8, C*H*_2_), 2.84 (2H, t, *J* = 6.8, C*H*_2_), 3.21 (2H, br t, 2× C*H*), 3.44 (4H, d, *J* = 2.0, 2× C*H*_2_), 7.37 (2H, s, exchange with D_2_O, SO_2_N*H*_2_), 7.45 (2H, d, *J* = 8.4, Ar-*H*), 7.76 (2H, d, *J* = 8.4, Ar-*H*); δ_C_ (100 MHz, DMSO-*d*_6_): 32.6, 41.5, 53.3, 75.8, 79.1, 125.6, 129.1, 141.9, 144.4; ESI-HRMS (*m*/*z*): calcd for [M + H]^+^ ion species C_14_H_17_N_2_O_2_S, 277.1005; found, 277.1009. Experimental data in agreement with reported data.^66^

#### 4-(2-(Bis((1-(2-(hydroxymethyl)-5-(5-methyl-2,4-dioxo-3,4-dihydropyrimidin-1(2*H*)-yl)tetrahydrofuran-3-yl)-1*H*-1,2,3-triazol-4-yl)methyl)amino)ethyl)benzenesulfonamide (**5b**)

Compound **5b** was obtained according to the general procedure C using **5a** as the starting material to afford the title compound **5b** as a white solid. 82% yield; δ_H_ (400 MHz, DMSO-*d*_6_): 1.85 (6H, s, 2× C*H*_3_), 2.72 (6H, m, overlapped signals, 3× C*H*_2_), 2.93 (2H, t, *J* = 7.2, C*H*_2_), 3.70 (4H, m, 2× C*H*_2_), 3.80 (4H, s, 2× C*H*_2_), 4.24 (2H, q, *J* = 3.5, 2× C*H*), 5.40 (4H; m, overlapped signals, 2× C*H*, exchange with D_2_O, 2× O*H*), 6.48 (2H, t, *J* = 6.4, 2× C*H*), 7.32 (2H, br s, exchange with D_2_O, SO_2_N*H*_2_), 7.41 (2H, d, *J* = 8.3, 2× Ar-*H*), 7.75 (2H, d, *J* = 8.3, 2× Ar-*H*), 7.87 (2H, s, 2× C*H*), 8.22 (2H, s, 2× C*H*), 11.40 (2H, br s, exchange with D_2_O, 2× N*H*). δ_C_ (100 MHz, DMSO-*d*_6_): 13.1, 33.4, 37.9, 48.2, 54.7, 60.1, 61.6, 84.8, 85.5, 110.5, 124.5, 126.5, 130.0, 137.2, 142.6, 144.6, 145.7, 151.4, 164.6; ESI-HRMS (*m*/*z*): calcd for [M + H]^+^ ion species C_34_H_43_N_12_O_10_S, 811.2940; found, 811.2951.

#### Prop-2-yn-1-yl 4-Chloro-3-sulfamoylbenzoate (**6a**)

To a stirring solution of 4-chloro-3-sulfamoylbenzoic acid **6** (1 equiv) in dry DMF, 1-ethyl-3-(3-dimethylaminopropyl)carbodiimide (EDC) HCl (1.2 equiv) was added at 0 °C. After 30 min, propargyl alcohol (1.2 equiv) and 4-dimethylaminopyridine (1.2 equiv) were added. The mixture was stirred at r.t. under N_2_ for an additional 3 h until consumption of the starting material. The reaction was quenched with slush and extracted with EtOAc (×3). The organic extract was washed with saturated aqueous NaHCO_3_, water, and brine; dried over Na_2_SO_4_; filtered-off; and concentrated under vacuum. The crude was purified by flash silica chromatography (40% EtOAc/Hx) to afford the title compound **6a** as a white solid. 40% yield; δ_H_ (400 MHz, DMSO-*d*_6_): 3.68 (1H, t, *J* = 2.4, C*H*), 5.05 (2H, d, *J* = 2.5, C*H*_2_), 7.86 (3H, m, 1× Ar-*H*, 2× SO_2_N*H*_2_), 8.16 (1H, dd, *J* = 2.2, 8.2, Ar-*H*), 8.56 (1H, d, *J* = 2.1, Ar-*H*); δ_C_ (100 MHz, DMSO-*d*_6_): 53.6, 79.0, 79.3, 129.0, 130.6, 133.4, 134.5, 136.7, 142.4, 165.5; ESI-HRMS (*m*/*z*): calcd for [M + H]^+^ ion species C_15_H_16_N_3_O_2_, 270.1237; found, 270.1237.

#### (1-((2-(Hydroxymethyl)-5-(5-methyl-2,4-dioxo-3,4-dihydropyrimidin-1(2*H*)-yl)tetrahydrofuran-3-yl)methyl)-1*H*-1,2,3-triazol-4-yl)methyl 4-Chloro-3-sulfamoylbenzoate (**6b**)

Compound **6b** was obtained according to the general procedure C using **6a** as a starting material to afford the title compound **6b** as a white solid. 21% yield; δ_H_ (400 MHz, DMSO-*d*_6_): 1.83 (3H, s, C*H*_3_), 2.69 (2H, m, C*H*_2_), 3.67 (2H, m, C*H*_2_), 4.22 (1H, q, *J* = 3.9, C*H*), 4.76 (2H, s, C*H*_2_), 5.29 (1H, br t, 1H, exchange with D_2_O, O*H*), 5.38 (1H, dt, *J* = 8.3, 5.29, C*H*), 6.43 (1H, t, *J* = 6.5, C*H*), 7.47 (2H, s, exchange with D_2_O, SO_2_N*H*_2_), 7.86 (2H, m, 2× Ar-*H*), 7.98 (1H, s, C*H*), 8.10 (1H, s, Ar-*H*), 8.38 (1H, s, C*H*), 11.3 (1H, br s, exchange with D_2_O, N*H*). δ_C_ (100 MHz, DMSO-*d*_6_):13.1, 27.5, 38.0, 60.4, 61.6, 84.8, 85.3, 110.5, 111.6, 116.9, 123.1, 124.6, 137.1, 142.0, 143.2, 151.3, 153.8, 163.2, 164.6, 167.1; ESI-HRMS (*m*/*z*): calcd for [M + H]^+^ ion species C_20_H_22_ClN_6_O_8_S, 541.0903; found, 541.0899.

#### Prop-2-ynyl 4-Sulfamoylbenzoate (**7a**)

To a stirring solution of 4-sulfamoylbenzoic acid **7** (2.0 g, 9.9 mmol) in dry DMF (40 mL) were successively added propargyl alcohol (1.17 mL, 19.8 mmol, 2.0 equiv), Et_3_N (2.8 mL, 19.9 mmol, 2.0 equiv), and EDC HCl (1.9 g, 9.9 mmol, 1.0 equiv). The solution was stirred at r.t. under N_2_ for an additional 4 h. The mixture was then concentrated under reduced pressure and ethyl acetate (40 mL) was added. The organic extract was washed with saturated aqueous NaHCO_3_ (40 mL) and back-extracted with ethyl acetate (40 mL). The organic layers were combined and washed with brine (40 mL), dried over Na_2_SO_4_, filtered, and evaporated. The crude oil was purified by flash silica chromatography (50% EtOAc/Hx) to afford the title compound **7a** as a white crystalline solid. 38% yield; δ_H_ (400 MHz, DMSO-*d*_6_): 3.63 (1H, t, *J* = 2.4, C*H*), 4.97 (2H, d, *J* = 2.8, C*H*_2_), 7.55 (2H, br s, exchange with D_2_O, SO_2_N*H*_2_), 7.98 (4H, m, 4× Ar*H*); δ_C_ (100 MHz, DMSO-*d*_6_): 53.0, 78.1, 78.3, 126.2, 130.0, 131.7, 148.3, 164.0. Experimental data in agreement with reported data.^67^

#### (1-(2-(Hydroxymethyl)-5-(5-methyl-2,4-dioxo-3,4-dihydropyrimidin-1(2*H*)-yl)tetrahydrofuran-3-yl)-1*H*-1,2,3-triazol-4-yl)methyl 4-Sulfamoylbenzoate (**7b**)

Compound **7b** was obtained according to the general procedure C using **7a** as the starting material to afford the title compound **7b** as a white solid. 30% yield; δ_H_ (400 MHz, DMSO-*d*_6_): 1.84 (3H, s, C*H*_3_), 2.74 (2H, m, C*H*_2_), 3.71 (2H, m, C*H*_2_), 4.28 (1H, m, C*H*), 5.32 (1H, t, *J* = 5.1, exchange with D_2_O, O*H*), 5.42 (1H, m, C*H*), 5.50 (2H, s, C*H*_2_), 6.46 (1H, t, *J* = 6.6, C*H*), 7.60 (2H, s, exchange with D_2_O, SO_2_N*H*_2_), 7.85 (1H, s, C*H*), 8.00 (2H, d, *J* = 8.4, 2× Ar-*H*), 8.18 (2H, d, *J* = 8.5, 2× Ar-*H*), 8.49 (1H, s, C*H*), 11.4 (1H, br s, exchange with D_2_O, N*H*); δ_C_ (100 MHz, DMSO-*d*_6_): 13.3, 38.0, 59.3, 60.5, 61.7, 84.8, 85.3, 110.5, 125.7, 127.3, 131.2, 133.0, 137.1, 142.8, 149.1, 151.3, 164.6, 165.4; ESI-HRMS (*m*/*z*): calcd for [M + H]^+^ ion species C_20_H_23_N_6_O_8_S, 507.1293; found, 507.1294.

#### *N*-(Prop-2-ynyl)-4-sulfamoylbenzamide (**8a**)

To a stirring solution of 4-sulfamoylbenzoic acid **7** (2.0 g, 9.9 mmol) and propargylamine (0.64 mL, 9.9 mmol, 1.0 equiv) in dry DMF (40 mL) were successively added *N*-hydroxybenzotriazole monohydrate (0.94 g, 6.6 mmol, 0.6 equiv), diisopropylethylamine (1.7 mL, 9.9 mmol, 1.0 equiv), and HBTU (3.8 g, 9.9 mmol, 1.0 equiv). The deep yellow solution was stirred at r.t. under N_2_ for 1 h when found complete by TLC. The mixture was concentrated under reduced pressure and ethyl acetate (40 mL) was added. The organic extract was washed with water (40 mL) and back-extracted with EtOAc (×3). The organic extracts were combined and washed with brine (50 mL). The organic layer was dried over Na_2_SO_4_, filtered, and evaporated to a crude white solid. Recrystallization from hot methanol/water (9:1) afforded the title compound **8a** as a white crystalline solid. 82% yield; δ_H_ (400 MHz, DMSO-*d*_6_): 3.12 (1H, t, *J* = 2.4, C*H*), 4.05 (2H, d, *J* = 5.6, 2.8, C*H*_2_), 7.45 (2H, br s, exchange with D_2_O, SO_2_N*H*_2_), 7.92 (4H, m, 4× Ar*H*), 9.09 (1H, t, *J* = 5.6, exchange with D_2_O, N*H*); δ_C_ (100 MHz, DMSO-*d*_6_): 29.0, 73.8, 81.7, 126.4, 128.6, 137.3, 147.1, 164.6. Experimental data in agreement with reported data.^67^

#### *N*-((1-(2-(Hydroxymethyl)-5-(5-methyl-2,4-dioxo-3,4-dihydropyrimidin-1(2*H*)-yl)tetrahydrofuran-3-yl)-1*H*-1,2,3-triazol-4-yl)methyl)-4-sulfamoylbenzamide (**8b**)

Compound **8b** was obtained according to the general procedure C using **8a** as the starting material to afford the title compound **8b** as a white solid. 20% yield; δ_H_ (400 MHz, DMSO-*d*_6_): 1.84 (3H, s, C*H*_3_), 2.71 (2H, m, C*H*_2_), 3.69 (2H, m, C*H*_2_), 4.25 (1H, t, *J* = 4.6, C*H*), 4.59 (2H, s, C*H*_2_), 5.30 (1H, m, exchange with D_2_O, O*H*), 5.38 (1H, m, C*H*), 6.45 (1H, t, *J* = 6.6, C*H*), 7.51 (2H, s, exchange with D_2_O, SO_2_N*H*_2_), 7.84 (1H, s, C*H*), 7.94 (2H, m, 2× Ar-*H*), 8.07 (2H, d, *J* = 8.5, 2× Ar-*H*), 8.24 (1H, s, C*H*), 9.25 (1H, br s, exchange with D_2_O, N*H*), 11.4 (1H, br s, exchange with D_2_O, N*H*); δ_C_ (100 MHz, DMSO-*d*_6_): 13.1, 35.9, 38.0, 60.1, 61.7, 84.8, 85.4, 110.5, 123.6, 126.5, 128.9, 137.1, 137.9, 145.9, 147.2, 151.3, 164.6, 166.0; ESI-HRMS (*m*/*z*): calcd for [M + H]^+^ ion species C_20_H_24_N_7_O_7_S, 506.1452; found, 506.1453.

#### 3-(3-(Prop-2-yn-1-yl)ureido)benzenesulfonamide (**9a**)

Compound **9a** was synthetized according to the general procedure B using phenyl (3-sulfamoylphenyl)carbamate **9** as the starting material. White solid, 65% yield; δ_H_ (400 MHz, DMSO-*d*_6_): 3.14 (1H, t, *J* = 2.4, C*H*), 3.93 (2H, dd, *J* = 2.2, 5.7, C*H*_2_), 6.59, (1H, t, *J* = 5.7, exchange with D_2_O, N*H*), 7.34 (2H, br s, exchange with D_2_O, SO_2_N*H*_2_), 7.40 (1H, dt, *J* = 8.2, 1.9, Ar*H*), 7.45 (1H, d, *J* = 7.8, Ar*H*), 7.57 (1H, dt, *J* = 8.1, 1.6, Ar*H*), 8.02 (1H, t, *J* = 2.0, Ar*H*), 8.98 (1H, br s, N*H*); δ_C_ (100 MHz, DMSO-*d*_6_): 29.7, 73.7, 82.8, 115.6, 119.3, 121.5, 130.2, 141.5, 145.5, 155.5; ESI-HRMS (*m*/*z*): calcd for [M + H]^+^ ion species C_10_H_12_N_3_O_3_S, 254.0594; found, 254.0592.

#### 3-(3-((1-(2-(Hydroxymethyl)-5-(5-methyl-2,4-dioxo-3,4-dihydropyrimidin-1(2*H*)-yl)tetrahydrofuran-3-yl)-1*H*-1,2,3-triazol-4-yl)methyl)ureido)benzenesulfonamide (**9b**)

Compound **9b** was obtained according to the general procedure C using **9a** as the starting material to afford the title compound **9b** as a yellow solid. 70% yield; δ_H_ (400 MHz, DMSO-*d*_6_): 1.84 (3H, s, C*H*_3_), 2.72 (2H, m, C*H*_2_), 3.68 (2H, m, C*H*_2_), 4.24 (1H, q, *J* = 4.1, C*H*), 4.41 (2H, d, *J* = 5.6, C*H*_2_), 5.30 (1H, t, *J* = 5.2, exchange with D_2_O, O*H*), 5.39 (1H, m, C*H*), 6.45 (1H, t, *J* = 6.6, C*H*), 6.75 (1H, t, *J* = 5.7, exchange with D_2_O, N*H*), 7.33 (2H, s, exchange with D_2_O, SO_2_N*H*_2_), 7.42 (2H, m, 2× Ar-*H*), 7.56 (1H, d, *J* = 8.2, Ar-*H*), 7.84 (1H, s, C*H*), 8.03 (1H, d, *J* = 2.0, Ar-*H*), 8.19 (1H, s, C*H*), 8.96 (1H, br s, exchange with D_2_O, N*H*), 11.4 (1H, br s, exchange with D_2_O, N*H*); δ_C_ (100 MHz, DMSO-*d*_6_): 13.1, 35.7, 38.0, 60.0, 61.6, 84.7, 85.3, 110.5, 115.5, 119.1, 121.4, 123.3, 130.1, 137.1, 141.7, 145.4, 146.6, 151.3, 155.7, 164.6; ESI-HRMS (*m*/*z*): calcd for [M + H]^+^ ion species C_20_H_25_N_8_O_7_S, 521.1561; found, 521.1556.

#### 4-(3-(Prop-2-yn-1-yl)ureido)benzenesulfonamide (**10a**)

Compound **10a** was synthetized according to the general procedure B using phenyl (4-sulfamoylphenyl)carbamate **10** as the starting material. White solid, 60% yield; δ_H_ (400 MHz, DMSO-*d*_6_): 3.14 (1H, t, *J* = 2.4, C*H*), 3.37 (2H, d, *J* = 2.4, C*H*_2_), 6.67, (1H, br t, exchange with D_2_O, N*H*), 7.19 (2H, br s, exchange with D_2_O, SO_2_N*H*_2_), 7.58 (2H, d, *J* = 8.2, 2× Ar*H*), 7.69 (2H, d, *J* = 8.2, 2× Ar*H*), 9.04 (1H, br s, exchange with D_2_O, N*H*); δ_C_ (100 MHz, DMSO-*d*_6_): 29.7, 73.8, 82.8, 118.0, 127.7, 137.3, 144.2, 155.5; ESI-HRMS (*m*/*z*): calcd for [M + H]^+^ ion species C_10_H_12_N_3_O_3_S, 254.0594; found, 254.0593.

#### 4-(3-((1-(2-(Hydroxymethyl)-5-(5-methyl-2,4-dioxo-3,4-dihydropyrimidin-1(2*H*)-yl)tetrahydrofuran-3-yl)-1*H*-1,2,3-triazol-4-yl)methyl)ureido)benzenesulfonamide (**10b**)

Compound **10b** was obtained according to the general procedure C using **10a** as the starting material to afford the title compound **10b** as a yellow solid. 47% yield; δ_H_ (400 MHz, DMSO-*d*_6_): 1.84 (3H, s, C*H*_3_), 2.71 (2H, m, C*H*_2_), 3.68 (2H, m, C*H*_2_), 4.24 (1H, q, *J* = 3.9, C*H*), 4.41 (2H, d, *J* = 5.6, C*H*_2_), 5.30 (1H, t, *J* = 5.2, exchange with D_2_O, O*H*), 5.39 (1H, dt, *J* = 8.3, 5.4, C*H*), 6.45 (1H, t, *J* = 6.6, C*H*), 6.84 (1H, t, *J* = 5.7, exchange with D_2_O, N*H*), 7.18 (2H, s, exchange with D_2_O, SO_2_N*H*_2_), 7.59 (2H, d, *J* = 8.9, 2× Ar-*H*), 7.71 (2H, d, *J* = 8.8, Ar-*H*), 7.84 (1H, s, C*H*), 8.20 (1H, s, C*H*), 9.04 (1H, br s, exchange with D_2_O, N*H*), 11.4 (1H, br s, exchange with D_2_O, N*H*); δ_C_ (100 MHz, DMSO-*d*_6_): 13.1, 35.7, 38.0, 60.0, 61.6, 84.7, 85.3, 110.5, 117.8, 123.2, 127.6, 137.8, 141.5, 144.3, 146.4, 151.3, 155.6, 164.6; ESI-HRMS (*m*/*z*): calcd for [M + H]^+^ ion species C_20_H_25_N_8_O_7_S, 521.1561; found, 521.1554.

#### 4-(Prop-2-yn-1-ylselanyl)aniline (**D**)

4-Selenocyanatoaniline **C** (1 equiv) was dissolved in EtOH, and NaBH_4_ (4 equiv) was added. The reaction was stirred for 20 min. Then, propargyl bromide (1.2 equiv) was added, and the reaction was stirred until consumption of the starting material. The reaction was quenched with NH_4_Cl saturated solution, extracted with EtOAc (×3), dried over Na_2_SO_4_, filtered, and concentrated in reduced pressure to give the desired product **D** as a yellow solid. 79% yield; δ_H_ (400 MHz, DMSO-*d*_6_): 2.26 (1H, s, C*H*), 3.34 (2H, d, *J* = 2.4, C*H*_2_), 3.76 (2H, br s, exchange with D_2_O, N*H*_2_), 6.59 (2H, d, *J* = 8.3, 2× Ar*H*), 7.44 (2H, d, *J* = 8.3, 2× Ar*H*); δ_C_ (100 MHz, DMSO-*d*_6_): 13.7, 71.9, 81.7, 115.8, 116.2, 136.8, 147.2; ESI-HRMS (*m*/*z*): calcd for [M + H]^+^ ion species C_9_H_10_NSe, 211.9973; found, 211.9969.

#### 1-(4-(4-(((4-Aminophenyl)selanyl)methyl)-1*H*-1,2,3-triazol-1-yl)-5-(hydroxymethyl)tetrahydrofuran-2-yl)-5-methylpyrimidine-2,4(1*H*,3*H*)-dione (**E**)

Synthetized according to the general procedure C using 4-(prop-2-yn-1-ylselanyl)aniline **D** as the starting material. Yellow solid. 20% yield; δ_H_ (400 MHz, DMSO-*d*_6_): 1.84 (3H, s, C*H*_3_), 2.69 (2H, m, C*H*_2_), 3.67 (2H, m, C*H*_2_), 4.04 (2H, s, C*H*_2_), 4.16 (2H, m, 2× C*H*), 5.34 (3H, br s, exchange with D_2_O, 1× O*H*, 2× *NH*_2_), 6.43 (1H, t, *J* = 6.6, C*H*), 6.49 (2H, d, *J* = 7.9, 2× Ar*H*), 7.13 (2H, d, *J* = 7.9, 2× Ar*H*), 7.84 (1H, s, C*H*), 7.96 (1H, s, C*H*), 11.4 (1H, br s, exchange with D_2_O, N*H*); δ_C_ (100 MHz, DMSO-*d*_6_): 13.2, 22.5, 38.0, 60.0, 61.5, 84.7, 85.3, 110.5, 113.5, 115.4, 123.6, 136.9, 137.1, 146.1, 149.2, 151.3, 164.6; ESI-HRMS (*m*/*z*): calcd for [M + H]^+^ ion species C_19_H_23_N_6_O_4_Se, 479.0942; found, 479.0932.

#### 3-(3-(4-(((1-(2-(Hydroxymethyl)-5-(5-methyl-2,4-dioxo-3,4-dihydropyrimidin-1(2*H*)-yl)tetrahydrofuran-3-yl)-1*H*-1,2,3-triazol-4-yl)methyl)selanyl)phenyl)thioureido)benzenesulfonamide (**11b**)

Compound **11b** was obtained by reacting compound **E** (1 equiv) dissolved in CH_3_CN with 3-isothiocyanatobenzenesulfonamide **11a** (1.1 equiv). The reaction was stirred and then quenched with H_2_O, assisting in the formation of a yellow precipitate that was filtered to afford the crude product. It was purified by silica gel column chromatography, eluting with 8% MeOH/DCM, to obtain the title compound **11b** as a white solid. 15% yield; δ_H_ (400 MHz, DMSO-*d*_6_): 1.84 (3H, s, C*H*_3_), 2.72 (2H, m, C*H*_2_), 3.62 (2H, m, C*H*_2_), 4.14 (1H, s, C*H*), 4.23 (2H, s, C*H*_2_), 5.31 (1H, m, exchange with D_2_O, O*H*), 5.38 (1H, m, C*H*), 6.38 (1H, t, *J* = 6.3, C*H*), 7.49 (8H, m, exchange with D_2_O, 2× SO_2_N*H*_2_, 4× Ar-*H*, 2× Ar-*H*), 7.75 (1H, d, *J* = 7.9, Ar-*H*), 7.84 (1H, s, C*H*), 8.00 (1H, s, Ar-*H*), 8.11 (1H, s, C*H*), 10.16 (2H, br s, exchange with D_2_O, 2× N*H*), 11.37 (1H, br s, exchange with D_2_O, N*H*); δ_C_ (100 MHz, DMSO-*d*_6_): 13.1, 20.9, 37.9, 60.0, 61.6, 84.7, 85.3, 110.5, 121.4, 122.3, 123.6, 125.1, 126.2, 127.7, 129.8, 133.4, 137.1, 139.2, 140.9, 145.1, 145.5, 151.3, 164.6, 180.6; ESI-HRMS (*m*/*z*): calcd for [M + H]^+^ ion species C_26_H_29_N_8_O_6_S_2_Se, 693.0812; found, 693.0822.

#### 4-(3-(4-(((1-(2-(Hydroxymethyl)-5-(5-methyl-2,4-dioxo-3,4-dihydropyrimidin-1(2*H*)-yl)tetrahydrofuran-3-yl)-1*H*-1,2,3-triazol-4-yl)methyl)selanyl)phenyl)thioureido)benzenesulfonamide (**12b**)

Compound **12b** was obtained by reacting compound **E** (1 equiv) dissolved in CH_3_CN with 4-isothiocyanatobenzenesulfonamide **12a** (1.1 equiv). The reaction was stirred and then quenched with H_2_O, assisting in the formation of a yellow precipitate that was filtered to afford the crude product. It was purified by silica gel column chromatography, eluting with 8% MeOH/DCM, to obtain the title compound **12b** as a white solid. 60% yield; δ_H_ (400 MHz, DMSO-*d*_6_): 1.84 (3H, s, C*H*_3_), 2.69 (2H, m, C*H*_2_), 3.67 (2H, m, C*H*_2_), 4.18 (1H, dd, *J* = 3.2, 5.7, C*H*), 4.28 (2H, s, C*H*_2_), 5.31 (1H, t, *J* = 5.3, exchange with D_2_O, O*H*), 5.34 (1H, m, C*H*), 6.43 (1H, t, *J* = 6.6, C*H*), 7.33 (2H, s, exchange with D_2_O, SO_2_N*H*_2_), 7.50 (4H, m, 4× Ar-*H*), 7.72 (2H, d, *J* = 8.7, 2× Ar-*H*), 7.80 (2H, d, *J* = 8.7, 2× Ar-*H*), 7.84 (1H, s, C*H*), 8.11 (1H, s, C*H*), 10.10 (1H, br s, exchange with D_2_O, N*H*), 10.15 (1H, br s, exchange with D_2_O, N*H*), 11.39 (1H, br s, exchange with D_2_O, N*H*); δ_C_ (100 MHz, DMSO-*d*_6_): 13.1, 20.9, 38.0, 60.0, 61.5, 84.7, 85.3, 110.5, 123.5, 125.1, 126.3, 127.1, 128.3, 133.3, 137.1, 139.2, 140.3, 143.5, 145.5, 151.3, 164.6, 180.3; ESI-HRMS (*m*/*z*): calcd for [M + H]^+^ ion species C_26_H_29_N_8_O_6_S_2_Se, 693.0812; found, 693.0819.

#### 2-Oxo-2,3-dihydrobenzo[*d*]oxazole-5-sulfonamide (**13a**)

A solution of 3-amino-4-hydroxybenzenesulfonamide **13** (2.76 g, 1.0 equiv) in dry tetrahydrofuran (90 mL) was treated with dropwise phosgene solution (∼20% in toluene, 1.2 equiv) at 0 °C, and then, the reaction was warmed to r.t. and stirred overnight. After the consumption of the starting material (TLC monitoring), the reaction was quenched with slush and acidified with 1 M aqueous solution of HCl, extracted with EtOAc (3× 20 mL), and the combined organic layers were washed with H_2_O (3× 20 mL), dried over Na_2_SO_4_, filtered, and concentrated in reduced pressure to give the desired product as a brown solid. 83% yield; δ_H_ (400 MHz, DMSO-*d*_6_): 7.41 (2H, s, exchange with D_2_O, SO_2_N*H*_2_), 7.49 (1H, d, *J* = 8.4, Ar-*H*), 7.52 (1H, d, *J* = 1.9, Ar-*H*), 7.60 (1H, dd, *J* = 1.9, 8.4, Ar-*H*), 12.03 (1H, s, exchange with D_2_O, N*H*); δ_C_ (100 MHz, DMSO-*d*_6_): 108.2, 110.5, 121.0, 131.6, 140.8, 146.3, 155.1; ESI-HRMS (*m*/*z*): calcd for [M + H]^+^ ion species C_7_H_7_N_2_O_4_S, 215.0121; found, 215.0122.

#### *N*,*N*-Dimethyl-*N*′-((2-oxo-2,3-dihydrobenzo[*d*]oxazol-5-yl)sulfonyl)formimidamide (**13b**)

A solution of **13a** (6.27 g, 1.0 equiv) in DMF (5 mL) was cooled to 0 °C and then treated with *N*,*N*-dimethylformamide dimethyl acetal (1.2 equiv). The reaction continued until the consumption of the starting material (TLC monitoring). The reaction was quenched with slush to obtain a precipitate that was filtered and washed with water (3× 5 mL) and dried under vacuum to afford **13b** as a white solid. 41% yield; δ_H_ (400 MHz, DMSO-*d*_6_): 2.93 (3H, t, *J* = 0.6, C*H*_3_), 3.17 (3H, t, *J* = 0.6, C*H*_3_), 7.43 (2H, m, 2× Ar-*H*), 7.55 (1H, dd, *J* = 1.8, 8.4, Ar-*H*), 8.25 (1H, s, C*H*), 12.01 (1H, s, exchange with D_2_O, N*H*); δ_C_ (100 MHz, DMSO-*d*_6_): 35.9, 41.8, 108.3, 110.5, 121.3, 131.6, 139.6, 146.3, 155.1, 160.7; ESI-HRMS (*m*/*z*): calcd for [M + H]^+^ ion species C_10_H_12_N_3_O_4_S, 270.0543; found, 270.0538.

#### *N*-((Dimethylamino)methyl)-2-oxo-3-(prop-2-yn-1-yl)-2,3-dihydrobenzo[*d*]oxazole-5-sulfonamide (**13c**)

Compound **13b** (2.0 g, 1.0 equiv) was treated with potassium carbonate (1.0 equiv) in dry DMF (5 mL) and the suspension was stirred at r.t. for 20 min. Then, propargyl bromide (1.2 equiv) was added and the reaction was stirred at r.t. until the starting material was consumed (TLC monitoring). The reaction was quenched with slush, and the precipitate formed was collected by filtration, washed with Et_2_O (3× 5 mL), and dried under vacuum to obtain the desired compound as a brown solid. 90% yield; δ_H_ (400 MHz, DMSO-*d*_6_): 2.94 (3H, t, *J* = 0.7, C*H*_3_), 3.19 (3H, t, *J* = 0.6, C*H*_3_), 3.53 (1H, t, *J* = 2.5, C*H*), 4.84 (2H, d, *J* = 2.5, C*H*_2_), 7.54 (1H, d, *J* = 8.4, Ar-*H*), 7.65 (1H, dd, *J* = 1.8, 8.4, Ar-*H*), 7.80 (1H, d, *J* = 1.8, Ar-*H*), 8.27 (1H, s, C*H*); δ_C_ (100 MHz, DMSO-*d*_6_): 32.6, 36.0, 41.8, 77.0, 77.7, 108.5, 111.0, 122.1, 131.3, 140.1, 144.8, 153.8, 160.7; ESI-HRMS (*m*/*z*): calcd for [M + H]^+^ ion species C_13_H_14_N_3_O_4_S, 308.0700; found, 308.0698.

#### 2-Oxo-3-(prop-2-yn-1-yl)-2,3-dihydrobenzo[*d*]oxazole-5-sulfonamide (**13d**)

Compound **13c** (3.4 g, 1.0 equiv) was dissolved in a 1.5 M HCl in MeOH solution (30 mL), and the reaction was stirred at 60 °C in a sealed tube for 4 h, concentrated under vacuum to give a precipitate that was washed water (3× 5 mL) and then with Et_2_O (3× 5 mL), and dried under vacuum to afford the desired product as a brown solid. 55% yield; δ_H_ (400 MHz, DMSO-*d*_6_): 3.53 (1H, t, *J* = 2.5, C*H*), 4.81 (2H, d, *J* = 2.5, C*H*_2_), 7.49 (2H, s, exchange with D_2_O, SO_2_NH_2_), 7.61 (1H, d, *J* = 8.4, Ar-*H*), 7.72 (1H, dd, *J* = 1.9, 8.4, Ar-*H*), 7.83 (1H, d, *J* = 1.9, Ar-*H*); δ_C_ (100 MHz, DMSO-*d*_6_): 32.7, 35.0, 77.3, 108.2, 111.1, 122.0, 131.1, 141.3, 144.8, 153.9; ESI-HRMS (*m*/*z*): calcd for [M + H]^+^ ion species C_10_H_9_N_2_O_4_S, 253.0278; found, 253.0280.

#### 3-((1-(2-(Hydroxymethyl)-5-(5-methyl-2,4-dioxo-3,4-dihydropyrimidin-1(2*H*)-yl)tetrahydrofuran-3-yl)-1*H*-1,2,3-triazol-4-yl)methyl)-2-oxo-2,3-dihydrobenzo[*d*]oxazole-5-sulfonamide (**13e**)

Compound **13e** was obtained according to the general procedure C using **13d** as the starting material to afford the title compound **13e** as a white solid. 53% yield; δ_H_ (400 MHz, DMSO-*d*_6_): 1.84 (3H, s, C*H*_3_), 2.70 (2H, m, C*H*_2_), 3.68 (2H, m, C*H*_2_), 4.07 (1H, m, C*H*), 4.22 (1H, d, *J* = 5.4, C*H*), 5.22 (2H, s, C*H*_2_), 5.30 (1H, br t, exchange with D_2_O, O*H*), 6.44 (1H, t, *J* = 6.6, C*H*), 7.46 (2H, s, exchange with D_2_O, SO_2_N*H*_2_), 7.59 (1H, d, *J* = 8.4, Ar-*H*), 7.67 (1H, m, Ar-*H*), 7.78 (1H, s, Ar-*H*), 7.84 (1H, s, C*H*), 8.44 (1H, s, C*H*), 11.39 (1H, br s, exchange with D_2_O, N*H*); δ_C_ (100 MHz, DMSO-*d*_6_): 13.1, 37.9, 38.3, 60.3, 61.6, 84.8, 85.3, 108.1, 110.5, 110.8, 121.6, 124.3, 131.8, 137.0, 141.1, 142.1, 144.8, 151.3, 154.4, 164.6; ESI-HRMS (*m*/*z*): calcd for [M + H]^+^ ion species C_20_H_22_N_7_O_8_S, 520.1245; found, 520.1236.

#### 4-(Hex-5-yn-1-yloxy)-2*H*-chromen-2-one (**14a**)

Compound **14a** was synthetized according to the general procedure A using 4-hydroxy-2*H*-chromen-2-one **14** as the starting material and 6-chlorohex-1-yne as the alkyl halide at 100 °C. Compound **14a** was obtained as a white powder. 80% yield; δ_H_ (400 MHz, DMSO-*d*_6_): 1.70 (2H, m, C*H*_2_), 1.95 (2H, m, C*H*_2_), 2.31 (2H, m, C*H*_2_), 2.84 (1H, t, *J* = 2.5, C*H*), 4.28 (2H, t, *J* = 6.2, C*H*_2_), 5.92 (1H, s, Ar-*H*), 7.39 (1H, d, *J* = 7.8, Ar-*H*), 7.43 (1H, d, *J* = 8.1, Ar-*H*), 7.69 (1H, t, *J* = 8.1, Ar-*H*), 7.85 (1H, d, *J* = 7.8, Ar-*H*); δ_C_ (100 MHz, DMSO-*d*_6_): 18.4, 25.6, 28.1, 70.0, 72.5, 85.2, 91.5, 116.3, 117.5, 123.8, 125.2, 133.7, 153.8, 162.7, 165.9; ESI-HRMS (*m*/*z*): calcd for [M + H]^+^ ion species C_15_H_15_O_3_, 243.1016; found, 243.1016.

#### 1-(5-(Hydroxymethyl)-4-(4-(4-((2-oxo-2*H*-chromen-4-yl)oxy)butyl)-1*H*-1,2,3-triazol-1-yl)tetrahydrofuran-2-yl)-5-methylpyrimidine-2,4(1*H*,3*H*)-dione (**14b**)

Compound **14b** was obtained according to the general procedure C using **14a** as the starting material to afford the title compound **14b** as a white solid. 45% yield; δ_H_ (400 MHz, DMSO-*d*_6_): 1.84 (3H, s, C*H*_3_), 1.89 (2H, m, 2× C*H*_2_), 2.70 (2H, m, C*H*_2_), 2.78 (2H, m, C*H*_2_), 3.68 (2H, m, C*H*_2_), 4.22 (1H, m, C*H*), 4.29 (2H, m, C*H*_2_), 5.34 (2H, m, 1× C*H*, exchange with D_2_O, 1× O*H*), 5.93 (1H, br s, Ar-*H*), 6.45 (1H, br t, C*H*), 7.41 (2H, m, 1× Ar-*H*, 1× C*H*), 7.70 (1H, t, *J* = 7.9, Ar-*H*), 7.86 (2H, m, 1× Ar-*H*, 1× C*H*), 8.13 (1H, s, Ar-*H*), 11.4 (1H, br s, exchange with D_2_O, N*H*); δ_C_ (100 MHz, DMSO-*d*_6_):13.2, 25.5, 26.2, 28.4, 38.0, 59.9, 61.7, 70.1, 84.8, 85.4, 91.4, 110.5, 116.2, 117.4, 122.5, 123.7, 125.1, 133.6, 137.1, 147.8, 151.3, 153.7, 162.6, 164.6, 165.9; ESI-HRMS (*m*/*z*): calcd for [M + H]^+^ ion species C_25_H_28_N_5_O_7_, 510.1983; found, 510.1989.

#### 6-(Prop-2-ynyloxy)-2*H*-chromen-2-one (**15a**)

Compound **15a** was synthetized according to the general procedure A using 6-hydroxy-2*H*-chromen-2-one **15** as the starting material and propargyl bromide 80% in toluene as the alkyl halide. The reaction was performed at r.t. Compound **15a** was obtained as a white powder: 65% yield; δ_H_ (400 MHz, DMSO-*d*_6_): 3.64 (1H, br t, C*H*), 4.90 (2H, d, *J* = 2.1, C*H*_2_), 6.54 (1H, d, *J* = 9.6, Ar-*H*), 7.30 (1H, dd, *J* = 2.9, 9.0, Ar-*H*), 7.38 (1H, d, *J* = 2.9, Ar-*H*), 7.41 (1H, d, *J* = 9.0, Ar-*H*), 8.06 (1H, d, *J* = 9.6, Ar-*H*); δ_C_ (100 MHz, DMSO-*d*_6_): 56.0, 78.6, 78.9, 112.3, 116.8, 117.4, 119.2, 120.0, 144.0, 148.3, 153.4, 160.1; ESI-HRMS (*m*/*z*): calcd for [M + H]^+^ ion species C_12_H_9_O_3_, 201.0546; found, 201.0543. Experimental data in agreement with reported data.^68^

#### 1-(5-(Hydroxymethyl)-4-(4-(((2-oxo-2*H*-chromen-6-yl)oxy)methyl)-1*H*-1,2,3-triazol-1-yl)tetrahydrofuran-2-yl)-5-methylpyrimidine-2,4(1*H*,3*H*)-dione (**15b**)

Compound **15b** was obtained according to the general procedure C using **15a** as the starting material to afford the title compound **15b** as a white solid. 91% yield; δ_H_ (400 MHz, DMSO-*d*_6_): 1.85 (3H, s, CH_3_), 2.74 (2H, m, C*H*_2_), 3.70 (2H, m, C*H*_2_), 4.26 (1H, q, *J* = 3.5, C*H*), 5.25 (2H, s, C*H*_2_), 5.45 (2H, m, overlapped signals, 1× C*H*, exchange with D_2_O, 1× O*H*), 6.47 (1 H, t, *J* = 6.5, C*H*), 6.53 (1H, d, *J* = 9.6, Ar*H*), 7.33 (1H, dd, *J* = 2.8, 9.0, Ar*H*), 7.39 (1H, d, *J* = 9.0, Ar*H*), 7.48 (1H, d, *J* = 2.8, Ar*H*), 7.88 (1H, s, C*H*), 8.07 (1H, d, *J* = 9.6, Ar*H*), 8.54 (1H, s, C*H*), 11.37 (1H, br s, exchange with D_2_O, N*H*). δ_C_ (100 MHz, DMSO-*d*_6_):13.1, 38.0, 60.3, 61.6, 62.6, 84.8, 85.4, 110.5, 112.9, 117.5, 118.3, 120.1, 120.9, 125.4, 137.1, 143.4, 144.9, 148.9, 151.3, 155.2, 161.0, 164.6; ESI-HRMS (*m*/*z*): calcd for [M + H]^+^ ion species C_22_H_22_N_5_O_7_, 468.1514; found, 468.1508.^69^

#### 6-(Pent-4-yn-1-yloxy)-2*H*-chromen-2-one (**16a**)

Compound **16a** was synthetized according to the general procedure A using 6-hydroxy-2*H*-chromen-2-one **15** as the starting material and 5-chloropent-1-yne as the alkyl halide at 100 °C. Compound **16a** was obtained as a white powder. 79% yield; δ_H_ (400 MHz, DMSO-*d*_6_): 1.94 (2H, m, C*H*_2_), 2.38 (2H, dd, *J* = 4.9, 6.8, C*H*_2_), 2.87 (1H, s, C*H*), 4.11 (2H, t, *J* = 6.1, C*H*_2_), 6.53 (1H, d, *J* = 9.6, Ar*-H*), 7.24 (1H, dd, *J* = 2.6, 9.0, Ar*-H*), 7.34 (1H, d, *J* = 2.4, Ar*-H*), 7.37 (1H, d, *J* = 9.0, Ar*-H*), 8.04 (1H, d, *J* = 9.6, Ar*-H*); δ_C_ (100 MHz, DMSO-*d*_6_):15.6, 28.7, 67.7, 72.7, 84.6, 112.5, 117.6, 118.4, 120.3, 120.9, 145.1, 148.9, 155.9, 161.2; ESI-HRMS (*m*/*z*): calcd for [M + H]^+^ ion species C_14_H_13_O_3_, 229.0859; found, 229.0861. Experimental data in agreement with reported data.^70^

#### 1-(5-(Hydroxymethyl)-4-(4-(3-((2-oxo-2*H*-chromen-6-yl)oxy)propyl)-1*H*-1,2,3-triazol-1-yl)tetrahydrofuran-2-yl)-5-methylpyrimidine-2,4(1*H*,3*H*)-dione (**16b**)

Compound **16b** was obtained according to the general procedure C using **16a** as the starting material to afford the title compound **16b** as a white solid. 27% yield; δ_H_ (400 MHz, DMSO-*d*_6_): 1.84 (3H, s, C*H*_3_), 2.14 (2H, q, *J* = 6.8, C*H*_2_), 2.71 (2H, m, C*H*_2_), 2.85 (2H, t, *J* = 7.5, C*H*_2_), 3.69 (2H, m, C*H*_2_), 4.12 (2H, t, *J* = 6.3, C*H*_2_), 4.23 (1H, q, *J* = 4.1, C*H*), 5.34 (2H, m, 1× C*H*, exchange with D_2_O, 1× O*H*), 6.45 (1H, t, *J* = 6.6, C*H*), 6.52 (1H, d, *J* = 9.5, 1× Ar-*H*), 7.25 (1H, dd, *J* = 2.9, 9.0, 1× Ar-*H*), 7.32 (1H, d, *J* = 2.9, 1× Ar-*H*), 7.38 (1H, d, *J* = 8.9, 1× Ar-*H*), 7.85 (1H, s, C*H*), 8.04 (1H, d, *J* = 9.5, 1× Ar-*H*), 8.15 (1H, s, C*H*), 11.4 (1H, br s, exchange with D_2_O, N*H*); δ_C_ (100 MHz, DMSO-*d*_6_):13.1, 22.5, 29.2, 38.0, 59.9, 61.6, 68.3, 84.8, 85.4, 110.5, 112.3, 117.4, 118.2, 120.1, 120.8, 122.5, 137.1, 144.9, 147.3, 148.7, 151.3, 155.8, 161.0, 164.1; ESI-HRMS (*m*/*z*): calcd for [M + H]^+^ ion species C_24_H_26_N_5_O_7_, 496.1827; found, 496.1830.

#### 7-(Prop-2-ynyloxy)-2*H*-chromen-2-one (**17a**)

Compound **17a** was synthetized according to the general procedure A using 7-hydroxy-2*H*-chromen-2-one **17** as the starting material and propargyl bromide 80% in toluene as the alkyl halide. The reaction was performed at r.t. Compound **17a** was obtained as a white powder. 73% yield; δ_H_ (400 MHz, DMSO-*d*_6_): 3.69 (1H, t, *J* = 2.4, C*H*), 4.97 (2H, d, *J* = 2.4, C*H*_2_), 6.36 (1H, d, *J* = 9.6, Ar-*H*), 7.03 (1H, dd, *J* = 2.4, 8.6, Ar-*H*), 7.09 (1H, d, *J* = 2.4, Ar-*H*), 7.70 (1H, d, *J* = 8.6, Ar-*H*), 8.04 (1H, d, *J* = 9.6, Ar-*H*). Experimental data in agreement with reported data.^70^

#### 1-(5-(Hydroxymethyl)-4-(4-(((2-oxo-2*H*-chromen-7-yl)oxy)methyl)-1*H*-1,2,3-triazol-1-yl)tetrahydrofuran-2-yl)-5-methylpyrimidine-2,4(1*H*,3*H*)-dione (**17b**)

Compound **17b** was obtained according to the general procedure C using **17a** as the starting material to afford the title compound **17b** as a white solid. 91% yield; δ_H_ (400 MHz, DMSO-*d*_6_): 1.85 (3H, s, C*H*_3_), 2.73 (2H, m, C*H*_2_), 3.69 (2H, m, C*H*_2_), 4.27 (1H, q, *J* = 3.5, C*H*), 5.32 (2H, s, C*H*_2_), 5.36 (1H, t, *J* = 5.0, exchange with D_2_O, O*H*), 5.46 (1 H, m, C*H*), 6.34 (1H, d, *J* = 9.5, Ar*H*), 6.47 (1 H, t, *J* = 6.5, C*H*), 7.07 (1H, dd, *J* = 2.4, 8.6, Ar*H*), 7.21 (1H, d, *J* = 2.4, Ar*H*), 7.69 (1H, d, *J* = 8.6, Ar*H*), 7.86 (1H, s, C*H*), 8.04 (1H, d, *J* = 9.5, Ar*H*), 8.53 (1H, s, C*H*), 11.38 (1H, br s, exchange with D_2_O, N*H*). δ_C_ (100 MHz, DMSO-*d*_6_): 13.1, 38.1, 60.4, 61.7, 62.6, 84.9, 85.5, 102.5, 110.6, 113.5, 113.6, 113.8, 125.7, 130.5, 137.2, 143.1, 145.2, 151.4, 156.2, 161.2, 162.0, 164.0; ESI-HRMS (*m*/*z*): calcd for [M + H]^+^ ion species C_22_H_22_N_5_O_7_, 468.1514; found, 468.1520.^51^

#### 7-(Pent-4-yn-1-yloxy)-2*H*-chromen-2-one (**18a**)

Compound **18a** was synthetized according to the general procedure A using 7-hydroxy-2*H*-chromen-2-one **17** as the starting material and 5-chloropent-1-yne as the alkyl halide at 100 °C. Compound **18a** was obtained as a white powder. 71% yield; δ_H_ (400 MHz, DMSO-*d*_6_): 1.95 (2H, m, C*H*_2_), 2.38 (2H, m, C*H*_2_), 2.88 (1H, d, *J* = 1.8, C*H*), 4.18 (2H, t, *J* = 6.0, C*H*_2_), 6.33 (1H, d, *J* = 9.4, Ar-*H*), 6.99 (1H, d, *J* = 8.6, Ar-*H*), 7.04 (1H, s, Ar-*H*), 7.67 (1H, d, *J* = 8.5, Ar-*H*), 8.03 (1H, d, *J* = 9.5, Ar-*H*); δ_C_ (100 MHz, DMSO-*d*_6_): 15.5, 28.5, 67.8, 72.7, 84.5, 102.2, 113.4, 113.5, 113.7, 130.6, 145.3, 156.4, 161.3, 162.7; ESI-HRMS (*m*/*z*): calcd for [M + H]^+^ ion species C_14_H_13_O_3_, 229.0859; found, 229.0857.

#### 1-(5-(Hydroxymethyl)-4-(4-(3-((2-oxo-2*H*-chromen-7-yl)oxy)propyl)-1*H*-1,2,3-triazol-1-yl)tetrahydrofuran-2-yl)-5-methylpyrimidine-2,4(1*H*,3*H*)-dione (**18b**)

Compound **18b** was obtained according to the general procedure C using **18a** as the starting material to afford the title compound **18b** as a white solid. 21% yield; δ_H_ (400 MHz, DMSO-*d*_6_): 1.84 (3H, s, C*H*_3_), 2.13 (2H, m, C*H*_2_), 2.70 (2H, m, C*H*_2_), 2.86 (2H, m, C*H*_2_), 3.69 (2H, m, C*H*_2_), 4.21 (3H, m, 1× C*H*_2_, 1× C*H*), 5.34 (2H, m, 1× C*H*, exchange with D_2_O, 1× O*H*), 6.32 (1H, d, *J* = 9.47, 1× Ar-*H*), 6.45 (1H, m, C*H*), 7.01 (2H, m, 1× C*H*, 1× Ar-*H*), 7.66 (1H, m, Ar-*H*), 7.85 (1H, s, C*H*), 8.03 (1H, m, Ar-*H*), 8.15 (1H, s, C*H*), 11.4 (1H, br s, exchange with D_2_O, N*H*); δ_C_ (100 MHz, DMSO-*d*_6_):13.2, 22.4, 29.1, 38.0, 59.9, 61.6, 68.4, 84.8, 85.4, 102.1, 110.5, 113.2, 113.3, 113.6, 122.6, 130.4, 137.1, 145.2, 147.3, 151.3, 156.3, 161.2, 162.7, 164.1; ESI-HRMS (*m*/*z*): calcd for [M + H]^+^ ion species C_24_H_26_N_5_O_7_, 496.1827; found, 496.2000.

#### 7-(Hex-5-yn-1-yloxy)-2*H*-chromen-2-one (**19a**)

Compound **19a** was synthetized according to the general procedure A using 7-hydroxy-2*H*-chromen-2-one **17** as the starting material and 6-chlorohex-1-yne as the alkyl halide at 100 °C. Compound **19a** was obtained as a white powder. 80% yield; δ_H_ (400 MHz, DMSO-*d*_6_): 1.64 (2H, m, C*H*_2_), 1.86 (2H, m, C*H*_2_), 2.28 (2H, m, C*H*_2_), 2.82 (1H, t, *J* = 2.5, C*H*), 4.13 (2H, t, *J* = 6.4, C*H*_2_), 6.31 (1H, d, *J* = 9.5, Ar-*H*), 6.97 (1H, dd, *J* = 2.1, 8.6, Ar*-H*), 7.01 (1H, d, *J* = 2.1, Ar*-H*), 7.65 (1H, d, *J* = 8.6, Ar*-H*), 8.02 (1H, d, *J* = 9.5, Ar*-H*); δ_C_ (100 MHz, DMSO-*d*_6_):18.7, 25.9, 28.8, 68.8, 72.4, 85.3, 102.3, 113.3, 113.4, 113.7, 130.5, 145.3, 156.5, 161.3, 162.9; ESI-HRMS (*m*/*z*): calcd for [M + H]^+^ ion species C_15_H_15_O_3_, 243.1016; found, 243.1012.

#### 1-(5-(Hydroxymethyl)-4-(4-(4-((2-oxo-2*H*-chromen-7-yl)oxy)butyl)-1*H*-1,2,3-triazol-1-yl)tetrahydrofuran-2-yl)-5-methylpyrimidine-2,4(1*H*,3*H*)-dione (**19b**)

Compound **19b** was obtained according to the general procedure C using **19a** as the starting material to afford the title compound **19b** as a white solid. 21% yield; δ_H_ (400 MHz, DMSO-*d*_6_): 1.83 (7H, m, 2× C*H*_2_, 1× C*H*_3_), 2.70 (2H, m, C*H*_2_), 2.76 (2H, m, C*H*_2_), 3.71 (2H, m, C*H*_2_), 4.15 (2H, m, C*H*_2_), 4.23 (1H, m, C*H*), 5.34 (2H, m, 1× C*H*, exchange with D_2_O, 1× O*H*), 6.31 (1H, d, *J* = 9.4, Ar-*H*), 6.45 (1H, t, *J* = 6.5, C*H*), 6.96 (1H, m, Ar-*H*), 7.0 (1H, m, Ar-*H*), 7.64 (1H, d, *J* = 8.6, 1× Ar-*H*), 7.85 (1H, s, C*H*), 8.01 (1H, d, *J* = 9.4, 1× Ar-*H*), 8.11 (1H, s, C*H*), 11.4 (1H, br s, exchange with D_2_O, N*H*). δ_C_ (100 MHz, DMSO-*d*_6_):13.2, 25.6, 26.3, 28.9, 38.0, 59.9, 61.7, 68.9, 84.8, 85.4, 102.0, 110.5, 113.2, 113.4, 113.6, 122.4, 130.4, 137.1, 145.2, 147.8, 151.3, 156.3, 161.2, 162.7, 164.6; ESI-HRMS (*m*/*z*): calcd for [M + H]^+^ ion species C_25_H_28_N_5_O_7_, 510.1983; found, 510.1989.

#### 6-Prop-2-ynyloxy-benzo-[*e*][1,2]-oxathiine 2,2-dioxide (**20a**)

Compound **20a** was synthetized according to the general procedure A using 6-hydroxybenzo[e][1,2]oxathiine 2,2-dioxide **20** as the starting material and propargyl bromide 80% in toluene as the alkyl halide. Reaction performed at r.t. Compound **20a** was obtained as a white powder, pure: 85% yield; δ_H_ (400 MHz, DMSO-*d*_6_): 3.66 (1H, t, *J* = 2.4, C*H*), 4.90 (2H, d, *J* = 2.4, C*H*_2_), 7.24 (1H, dd, *J* = 3.0, 9.0, Ar-*H*), 7.38 (1H, d, *J* = 3.0, Ar-*H*), 7.45 (1H, d, *J* = 9.0, Ar-*H*), 7.55 (1H, d, *J* = 10.3, Ar-*H*), 7.68 (1H, d, *J* = 10.3, Ar-*H*); δ_C_ (100 MHz, DMSO-*d*_6_): 56.1, 78.8, 78.9, 115.2, 119.1, 119.6, 119.7, 123.3, 136.4, 145.0, 154.6; ESI-HRMS (*m*/*z*): calcd for [M + H]^+^ ion species C_11_H_9_O_4_S, 237.0216; found, 237.0212. Experimental data in agreement with reported data.^68^

#### 1-(4-(4-(((2,2-Dioxidobenzo[*e*][1,2]oxathiin-6-yl)oxy)methyl)-1*H*-1,2,3-triazol-1-yl)-5-(hydroxymethyl)tetrahydrofuran-2-yl)-5-methylpyrimidine-2,4(1*H*,3*H*)-dione (**20b**)

Compound **20b** was obtained according to the general procedure C using **20a** as the starting material to afford the title compound **20b** as a white solid. 78% yield; δ_H_ (400 MHz, DMSO-*d*_6_): 1.85 (3H, s, C*H*_3_), 2.74 (2H, m, C*H*_2_), 3.70 (2H, m, C*H*_2_), 4.26 (1H, q, *J* = 3.5, C*H*), 5.26 (2H, s, C*H*_2_), 5.37 (1H, br t, exchange with D_2_O, O*H*), 5.45 (1H, m, C*H*), 6.47 (1H, t, *J* = 6.5, C*H*), 7.29 (1H, dd, *J* = 3.0, 9.0, Ar*H*), 7.44 (1H, d, *J* = 9.0, Ar*H*), 7.47 (1H, d, *J* = 3.0, Ar*H*), 7.54 (1H, d, *J* = 10.3, Ar*H*), 7.68 (1H, d, *J* = 10.3, Ar*H*), 7.87 (1H, s, C*H*), 8.51 (1H, s, C*H*), 11.38 (1H, br s, exchange with D_2_O, N*H*). δ_C_ (100 MHz, DMSO-*d*_6_):13.1, 38.0, 60.3, 61.6, 62.7, 84.8, 85.4, 110.5, 115.7, 119.9, 120.4, 120.5, 124.0, 125.5, 137.1, 137.3, 143.3, 145.6, 151.4, 156.4, 164.9; ESI-HRMS (*m*/*z*): calcd for [M + H]^+^ ion species C_21_H_22_N_5_O_8_S, 504.1184; found, 504.1183.

### CA Inhibition

An Applied Photophysics stopped-flow instrument has been used for assaying the CA-catalyzed CO_2_ hydration activity.^53^ Phenol red (at a concentration of 0.2 mM) has been used as an indicator, working at the absorbance maximum of 557 nm, with 20 mM Hepes (pH 7.5) as a buffer, and 20 mM Na_2_SO_4_ (for maintaining the ionic strength constant), following the initial rates of the CA-catalyzed CO_2_ hydration reaction for a period of 10–100 s. The CO_2_ concentrations ranged from 1.7 to 17 mM for the determination of the kinetic parameters and inhibition constants. For each inhibitor, at least six traces of the initial 5–10% of the reaction have been used for determining the initial velocity. The uncatalyzed rates were determined in the same manner and subtracted from the total observed rates. Stock solutions of the inhibitor (0.1 mM) were prepared in distilled deionized water, and dilutions up to 0.01 nM were done thereafter with the assay buffer. The inhibitor and enzyme solutions were preincubated together for 15 min for sulfonamide derivatives and 6 h for coumarin and sulfocoumarin derivatives at r.t. prior to the assay, in order to allow for the formation of the **E**–**I** complex. The inhibition constants were obtained by nonlinear least-squares methods using PRISM 3 and the Cheng–Prusoff equation, as reported earlier, and represent the mean from at least three different determinations. All CA isoforms were recombinant ones obtained in-house as reported earlier.^53^

### Cocrystallization and X-ray Data Collection

The crystals of hCA II were obtained using the hanging drop vapor diffusion method using a 24-well Linbro plate. Two microliters of 10 mg/mL solution of hCA II in Tris-HCl 20 mM pH 8.0 was mixed with 2 μL of a solution of 1.5 M sodium citrate and 0.1 M Tris pH 8.0 and was equilibrated against the same solution at 296 K. The crystals of the protein grew in 1 week. Afterward, hCA II crystals were soaked in 5 mM inhibitor solution for 3 days. The crystals were flash-frozen at 100 K using a solution obtained by adding 15% (v/v) glycerol to the mother liquor solution as a cryoprotectant. Data on the crystal of the complex with **1b** was collected using synchrotron radiation at the ID-11.2C beamline at Elettra (Trieste, Italy) with a wavelength of 1.000 Å and a Pilatus3_6M Dectris CCD detector. Data on the crystal of the complex with **3b** was collected using synchrotron radiation at the MX1 beamline of the Australian Synchrotron. Data were integrated and scaled using the program XDS.^71^

### Structure Determination

The crystal structure of hCA II (PDB accession code: 3P58) without solvent molecules and other heteroatoms was used to obtain the initial phases of the structures using Refmac5.^72^ Unique reflections (5%) were selected randomly and excluded from the refinement data set for the purpose of *R*_free_ calculations. The initial |*F*_o_ – *F*_c_| difference electron density maps unambiguously showed the inhibitor molecules. Atomic models for inhibitors were calculated and energy-minimized using the program JLigand 1.0.40.^73^ Refinements were proceeded using normal protocols of positional, isotropic atomic displacement parameters alternating with manual building of the models using COOT.^74^ Solvent molecules were introduced automatically using the program ARP.^75^ The quality of the final models was assessed with COOT and RAMPAGE.^76^ Atomic coordinates were deposited in the Protein Data Bank (PDB accession code: 6YPW, 6WKA). Graphical representations were generated with Chimera.^77^

### In Vitro Telomerase Activity Assay

Human prostate cancer PC3 and human colorectal adenocarcinoma HT-29 cell lines (both from ATCC, Manassas, VA) were cultivated in RPMI-1640 cell media supplemented with 10% fetal bovine serum (Hyclone Laboratories, Logan, UK) at 37 °C in the presence of 5% CO_2_ and 95% humidity. Cell lines have been tested for mycoplasma contamination before the experiment using the Mycoplasma Detection Kit PlasmoTest (InvivoGen, San Diego, CA). The most potent CA IX and XII inhibitors **1b**, **7b**, **8b**, or **11b** were diluted to a final concentration of 20 μM and incubated with cells for 48 h. Telomerase activity was determined using the TRAP assay^15^ with modifications previously described by us.^78,79^ Briefly, cells were lysed in 10 mM Tris-HCl, pH 7.5, 1 mM MgCl_2_, 1 mM ethylene glycol-bis(2-aminoethylether)-*N*,*N*,*N*′,*N*′-tetracetic acid (EGTA), 0.1 mM phenylmethylsulfonylfluoride, 5 mM 2-mercaptoethanol, 0.5% 3-[(3-cholamidopropyl)dimethylammonium]-1-propanesulfonate hydrate, and 10% glycerol (all from Sigma-Aldrich, St. Louis, MO) and centrifuged for 30 min at 12,000*g*. Supernatants were stored at −80 °C. The protein concentration of cell extracts was determined using the BCA-1 protein assay kit (Sigma-Aldrich, St. Louis, MO). For elongation reaction, 5 μg of total protein and **CAI–TI** within the range of concentrations 0–100 μM were added to 30 μL of the reaction mixture containing 67 mM Tris-HCl, pH 8.8, 16.6 mM (NH_4_)_2_SO_4_, 0.01% Tween-20, 1.5 mM MgCl_2_, 1 mM EGTA (all from Sigma-Aldrich, St. Louis, MO), 0.25 mM each dNTPs (Evrogen, Moscow, Russia), and the telomerase substrate primer (TS-primer—AATCCGTCGAGCAGAGTT). Elongation was performed for 30 min at 37 °C and 10 min at 96 °C to inactivate the telomerase. Copy-extended primer 0.1 μL (CX-primer—CCCTTACCCTTACCCTTACCCTAA) and 2.5 units of Taq-polymerase were added to the elongation mixture, followed by the following PCR reaction: 94 °C—5 min; 30 cycles of 94 °C—30 s, 50 °C—30 s, and 72 °C—40 s; and 72 °C—5 min. PCR product visualization was performed using 12% nondenaturing PAAG electrophoresis and TBE buffer. Each sample (10 μL) was added to each well of the gel comb. Gels were stained with SYBR Green I (Invitrogen, Grand Island, NY), photographed under UV light in a ChemiDoc XRS imaging system, and analyzed using a GelAnalyzer 2010a. Statistical analysis involving the Student’s *t*-test was implemented with the Statistica 6.0 software (StatSoft, Tulsa, OK). To determine the IC_50_ and IC_90_ values (inhibitor concentration where the response is reduced by 50 and 90%, respectively), 1 μL of the reaction mixture was subjected to the real-time quantitative TRAP assay (RTQ-TRAP) as described by Hou and co-authors.^80^

### RNA Isolation and Real-Time RT-PCR

A previously described protocol was followed.^81^ Briefly, total RNA from cells was extracted using a PureLink RNA Mini kit (Life Technologies, Carlsbad, CA). Five micrograms of total RNA were reverse-transcribed using the RevertAid RT Kit (Invitrogen, Grand Island, NY) in a 25 μL reaction mixture, followed by real-time RT-PCR using DTprime5 (DNA Technology, Protvino, Russia). The reaction mix was prepared using Platinum SYBR Green qPCR Supermix-UDG (Invitrogen, Grand Island, NY) according to the manufacturer’s recommendations using the following primers (5′–3′). hTERT sense: GTCCGAGGTGTCCCTGAGTA; hTERT antisense: CAGGGCCTCGTCTTCTACAG; 18S sense: GGATCCATTGGAGGGCAAGT; 18S antisense: ACGAGCTTTTTAACTGCAGCAA (all primers were from Evrogen, Moscow, Russia). Two temperature cycles for annealing/extension were used. The fluorescence was measured at the end of each annealing step, and the melting curve analysis was performed at the end of the reaction (after the 35th cycle), between 60 and 95 °C, to assess the quality of the final PCR products. The standard curves indicating reaction effectiveness were generated using four serial dilutions (1:40, 1:80, 1:160, and 1:320) of total cDNAs. The relative level of hTERT mRNA was calculated using DTprime5 software. The levels of mRNA were normalized relative to the expression of the reference gene 18S. The data are presented as normalized mRNA levels of the studied genes using the averaged expression values of the reference gene.

### Statistical Analysis

Telomerase activity assay and measurement of hTERT gene expression were performed in quadruplicate. Statistical analysis using Student’s *t*-test was completed using Statistica 9.0 software (StatSoft, Tulsa, OK). Differences described as *p* ≤ 0.05 were considered significant. The values of IC_50_ and IC_90_ were calculated using Prism 6 software (GraphPad, San Diego, CA) according to the recommendations by Sebaugh.^82^

## Supporting Information Available

SMILES representation for compounds (CSV), data collection and atomic model refinement statistics, HPLC–DAD method for purity analysis, and HPLC traces (PDF)

## Abbreviations

CA: carbonic anhydrase
CAI(s): carbonic anhydrase inhibitor(s)
**AAZ**: acetazolamide
**AZT**: azidothymidine
TERT: telomerase reverse transcriptase
TERC: telomerase RNA component
RTQ-TRAP: real-time quantitative telomeric repeat amplification protocol
RT: reverse transcriptase
EGTA: ethylene glycol-bis(2-aminoethylether)-*N*,*N*,*N*′,*N*′-tetracetic acid
PMSF: phenylmethylsulfonylfluoride
CHAPS: 3-[(3-cholamidopropyl)dimethylammonium]-1-propanesulfonate hydrate
