# Supplementary material for: Azidothymidine “Clicked” into 1,2,3-Triazoles: First Report on Carbonic Anhydrase–Telomerase Dual-Hybrid Inhibitors
Source: J Med Chem. 2020 May 28;63(13):7392–409. doi: 10.1021/acs.jmedchem.0c00636 (PMC8154556; doi:10.1021/acs.jmedchem.0c00636)
Supplement: Supplementary file 1 — jm0c00636_si_001.pdf [file jm0c00636_si_001.pdf]

Supporting Information for

Azidothymidine (AZT) “clicked” into 1,2,3-Triazoles: First Report on Carbonic Anhydrase-Telomerase Dual Hybrid Inhibitors

Emanuela Berrino[1], Andrea Angeli[1], Dmitry D. Zhdanov[2, 3], Anna P. Kiryukhina[2], Andrea Milaneschi[1], Alessandro De Luca[1], Murat Bozdag[1], Simone Carradori[4], Silvia Selleri[1], Gianluca Bartolucci[1], Thomas S. Peat[5], Marta Ferraroni[6], Claudiu T. Supuran[1]* and Fabrizio Carta[1]*

[1] Università degli Studi di Firenze, NEUROFARBA Dept., Sezione di Scienze Farmaceutiche e Nutraceutiche, Via Ugo Schiff 6, 50019, Sesto Fiorentino (Florence), Italy

[2] Institute of Biomedical Chemistry, Pogodinskaya st. 10/8, 119121, Moscow, Russia

[3] Peoples Friendship University of Russia (RUDN University), Miklukho-Maklaya st. 6, 117198, Moscow, Russia.

[4] Department of Pharmacy "G. d'Annunzio" University of Chieti-Pescara Via dei Vestini 31, 66100, Chieti. Italy.

[5] CSIRO, 343 Royal Parade, Parkville, Victoria, 3052, Australia.

[6] Università di Firenze, Dipartimento di Chimica “Ugo Schiff”, Via della Lastruccia 3-13, 50019, Sesto Fiorentino (Florence), Italy.

*corresponding authors

Table of Contents

Summary of Data Collection and Atomic Model Refinement Statistics **S2**

HPLC-DAD method for purity analysis **S3**

HPLC traces **S4-S23**

Table S1. Summary of Data Collection and Atomic Model Refinement Statistics

	HCAII + 1b	HCAII + 3b
PDB ID	6YPW	6WKA
Wavelength (Å)	1.00000	1.00000
Space Group	$P2_1$	$P2_1$
Unit cell (a, b, c, α, β, γ) (Å,°)	42.387, 41.358, 72.075, 90.000, 104.385, 90.000	42.2, 41.5, 72.0, 90.0, 104.4, 90.0
Limiting resolution (Å)	41.36-1.10 (1.11-1.10)	41.5-1.34 (1.36-1.34)
Unique reflections	88794 (2400)	49061
Rmerge (%)	4.8 (43.7)	7.8 (47.2)
Rmeas (%)	5.5 (45.9)	-
Rpim (%)	3.1 (31.5)	3.1 (20.8)
Redundancy	6.3 (4.6)	7.1 (5.7)
Completeness overall (%)	90.7 (49.3)	95.0 (74.6)
<I/σ(I)>	19.5 (3.1)	14.3 (2.9)
CC (1/2)	0.999 (0.831)	0.999 (0.848)
**Refinement statistics**		
Resolution range (Å)	41.092-1.1	39.9-1.34
Unique reflections, working/free	84333/81550	1660/1270
Rfactor (%)	11.43	15.77
Rfree(%)	13.20	18.32
r.m.s.d. bonds(Å)	0.0123	0.010
r.m.s.d. angles (°)	1.8022	1.636
**Ramachandran statistics** (%)		
Most favored	97.7	96.9
additionally allowed	2.3	3.1
outlier regions	0.0	0.0
**Average B factor (Å^2)**		
All atoms	12.279	11.0
inhibitors	15.837	21.8
solvent	25.095	25.4

HPLC-DAD method for purity analysis

The employed chromatographic parameters to check the purity of studied compounds were reported as follows:

- column, Luna PFP length = 50 mm, internal diameter= 2 mm; particle size = 3 μm purchased from Phenomenex (Bologna, Italy);
- the eluents used were 5 mM ammonium formate in water solution (solvent A) and 10 mM ammonium formate in methanol (solvent B);
- flow rate and the injection volume were 0.35 mL min^{-1} and 5 μL, respectively.

The elution gradient is shown in **Table S2**.

Table S2: Elution gradient of mobile phase used for LC-DAD analysis

Time (min)	A (%)
0.00	90
8.00	10
14.00	10
14.01	90
20.00	90

.

HPLC traces

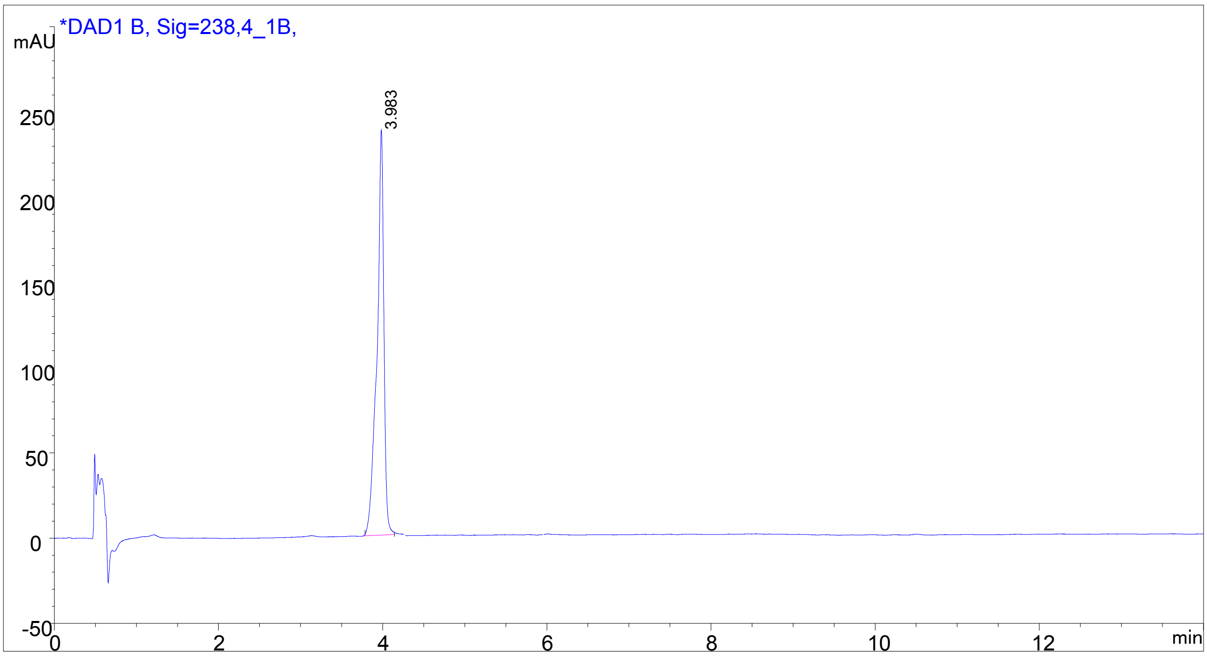

Figure S1. Chromatographic profile of **1b** monitored at λ=238 nm.

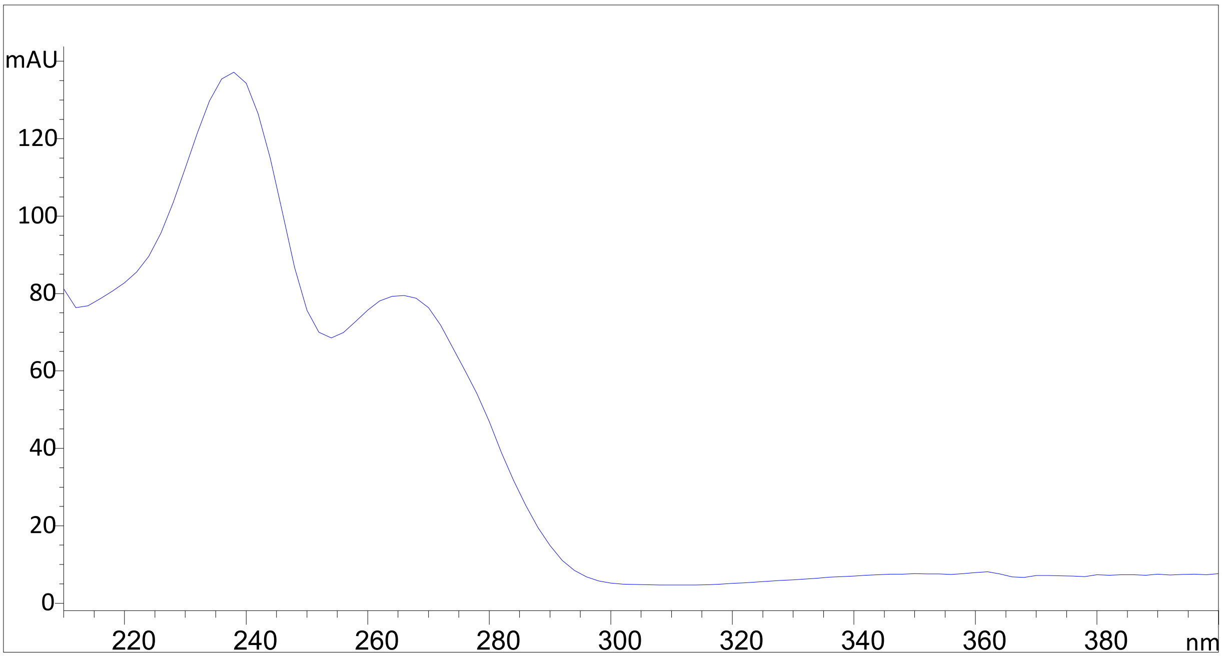

Figure S2. UV spectrum of the compound **1b**.

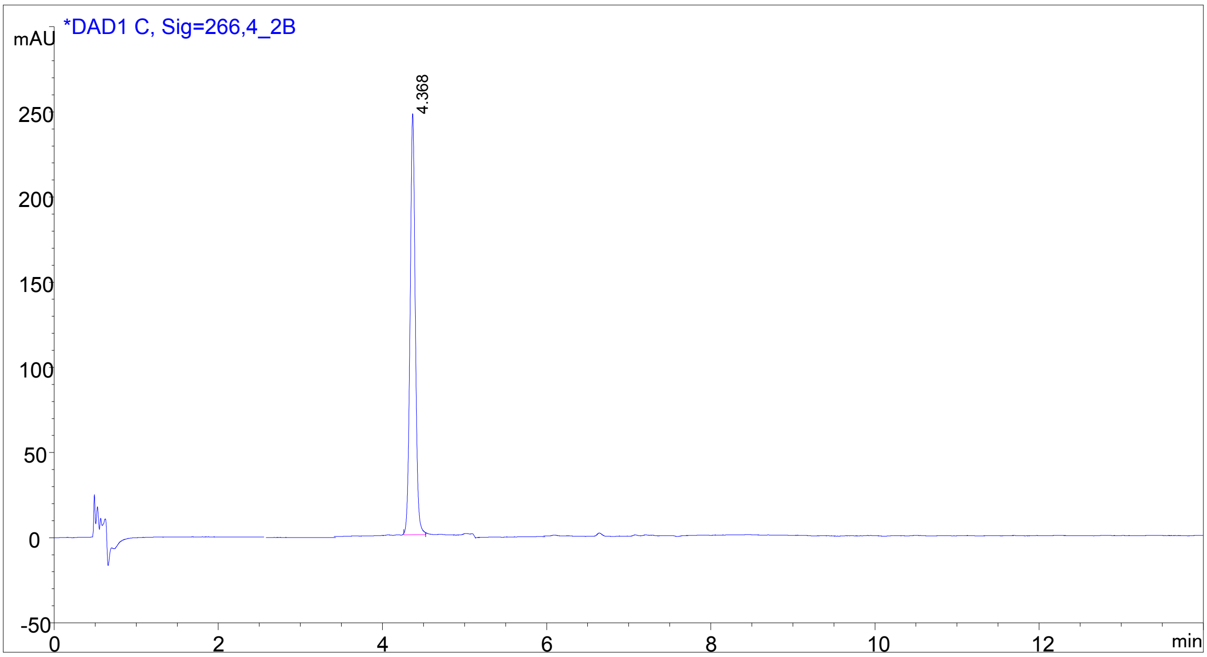

Figure S3. Chromatographic profile of **2b** monitored at λ=266 nm.

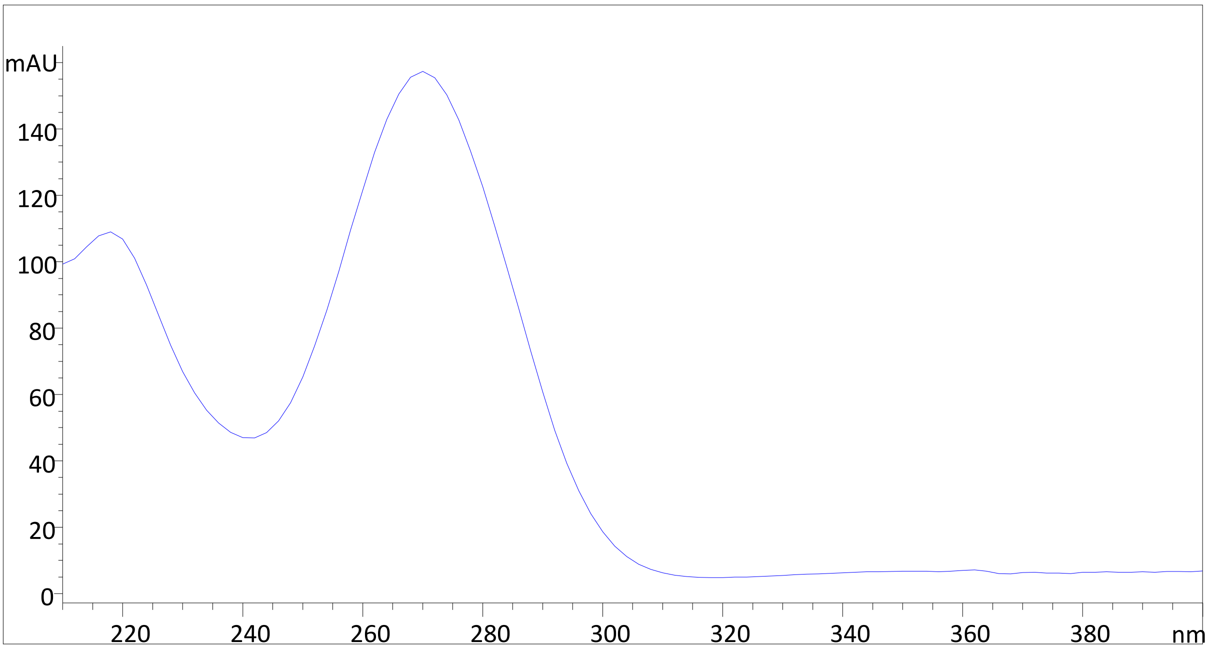

Figure S4. UV spectrum of the compound **2b**.

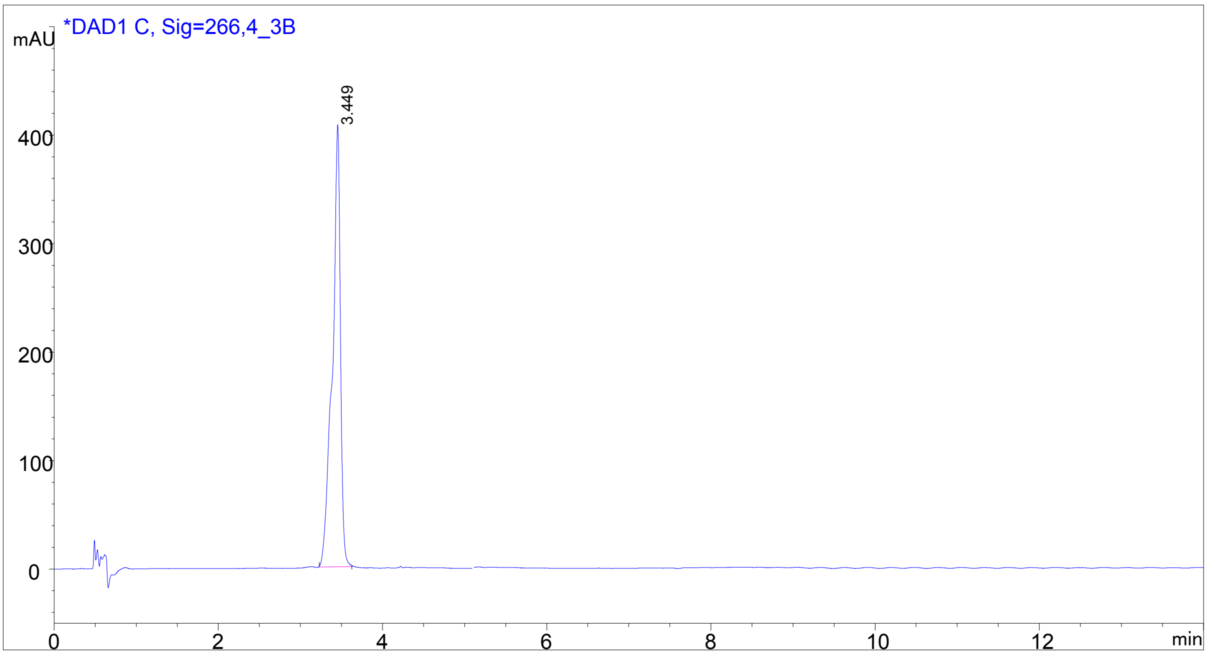

Figure S5. Chromatographic profile of **3b** monitored at λ=266 nm.

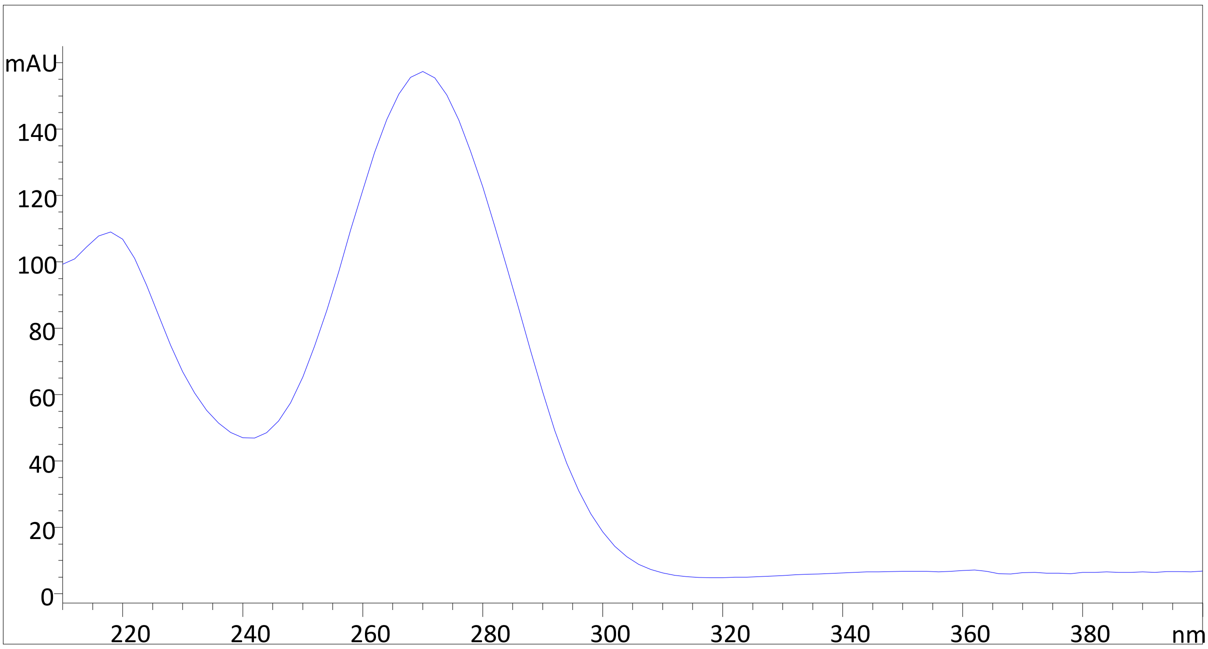

Figure S6. UV spectrum of the compound **3b**.

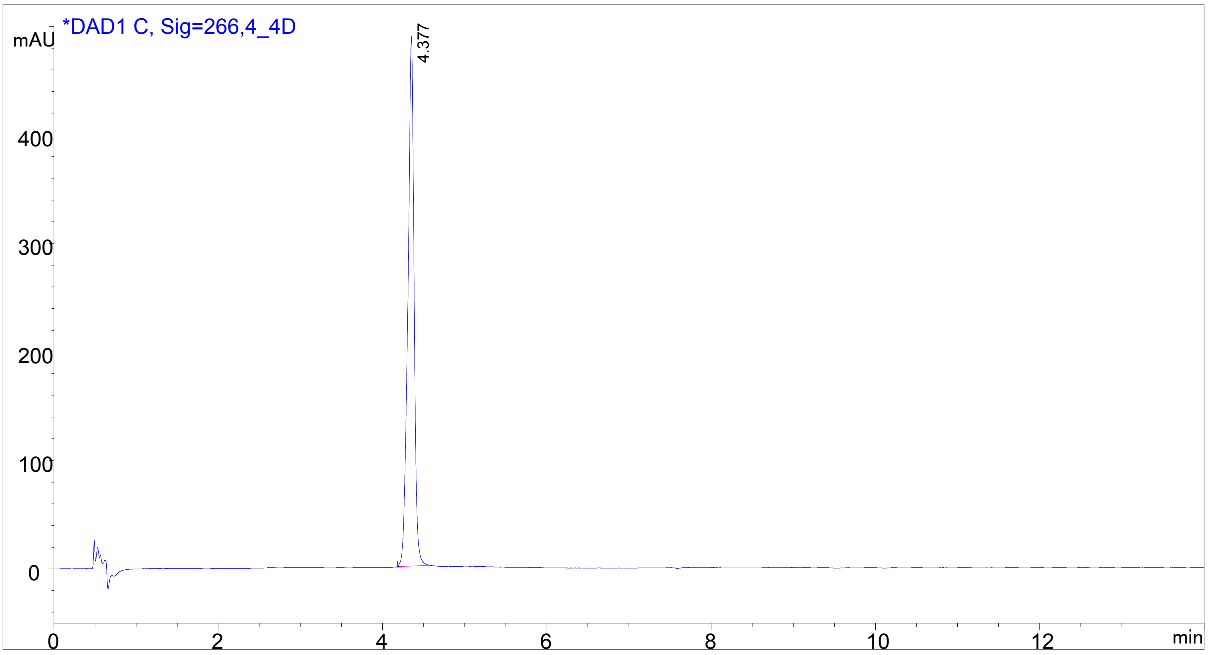

Figure S7. Chromatographic profile of **4d** monitored at λ=266 nm.

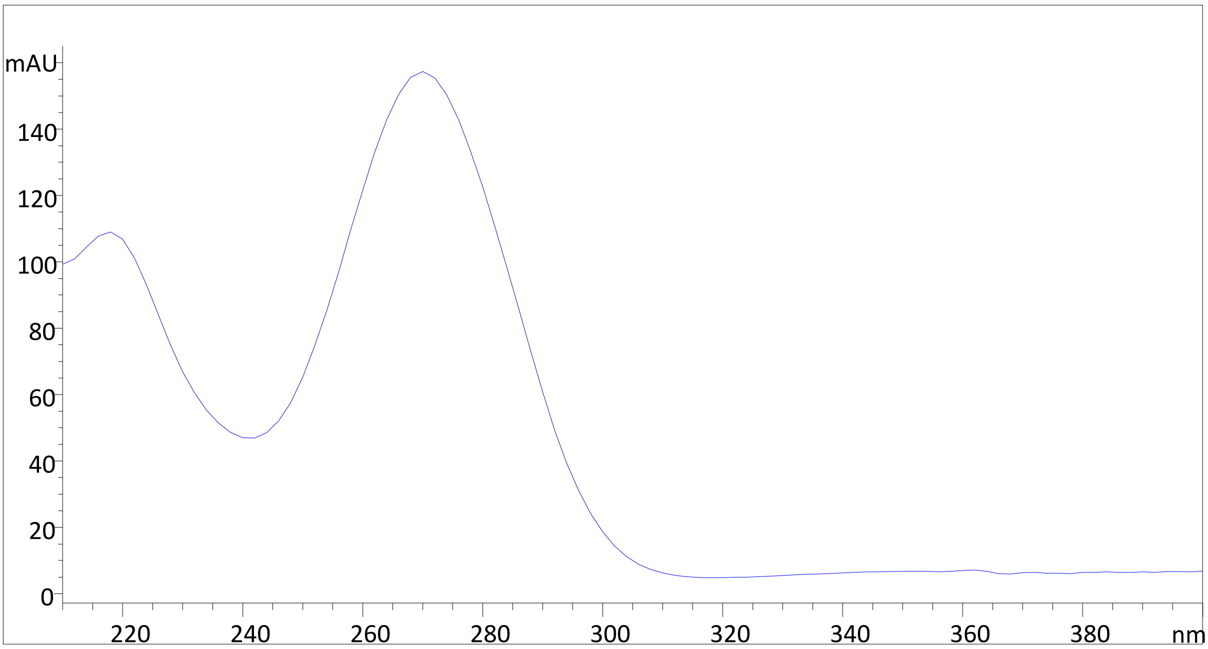

Figure S8. UV spectrum of the compound **4d**.

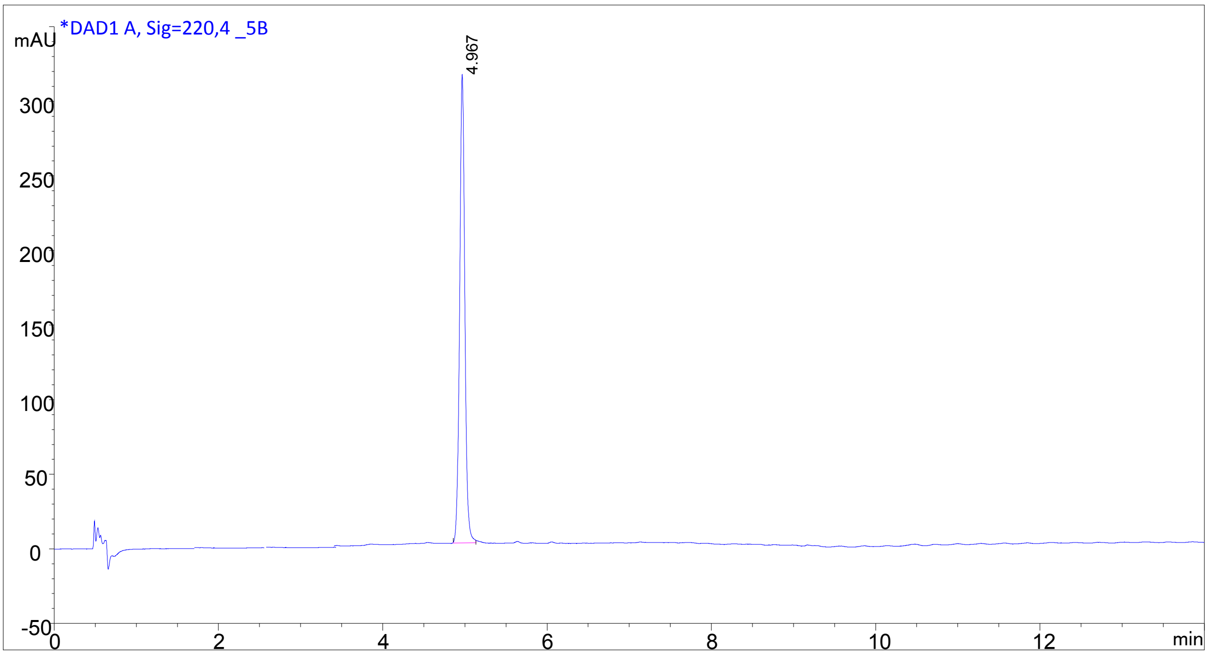

Figure S9. Chromatographic profile of **5b** monitored at λ=220 nm.

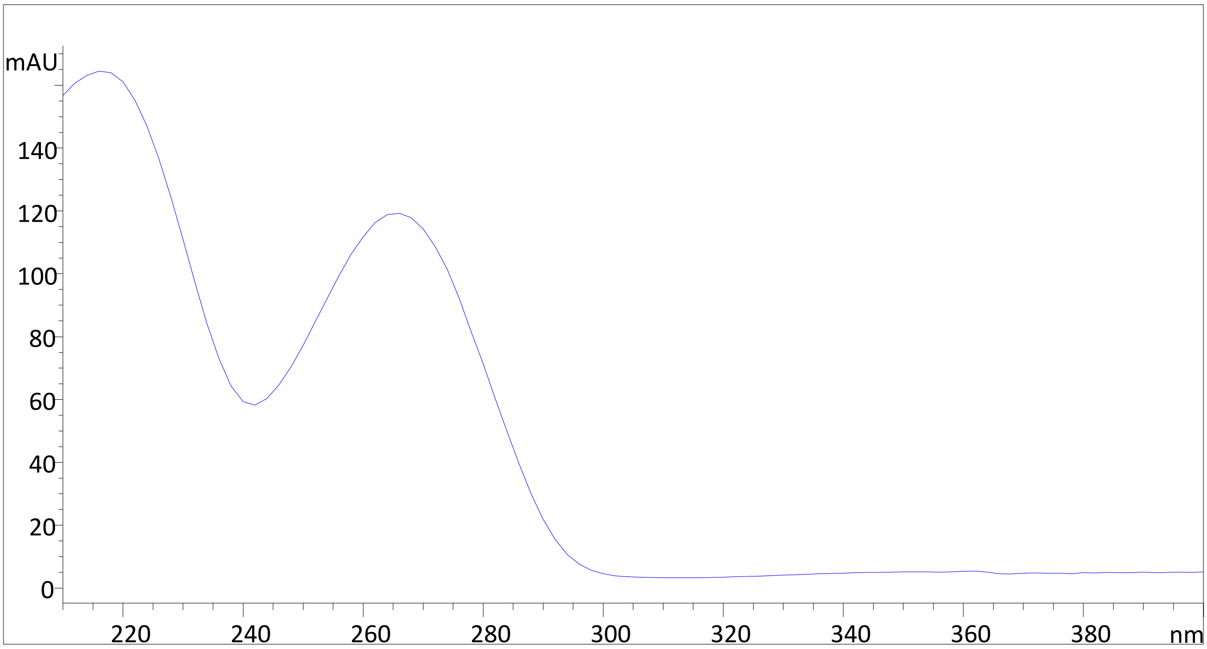

Figure S10. UV spectrum of the compound **5b**.

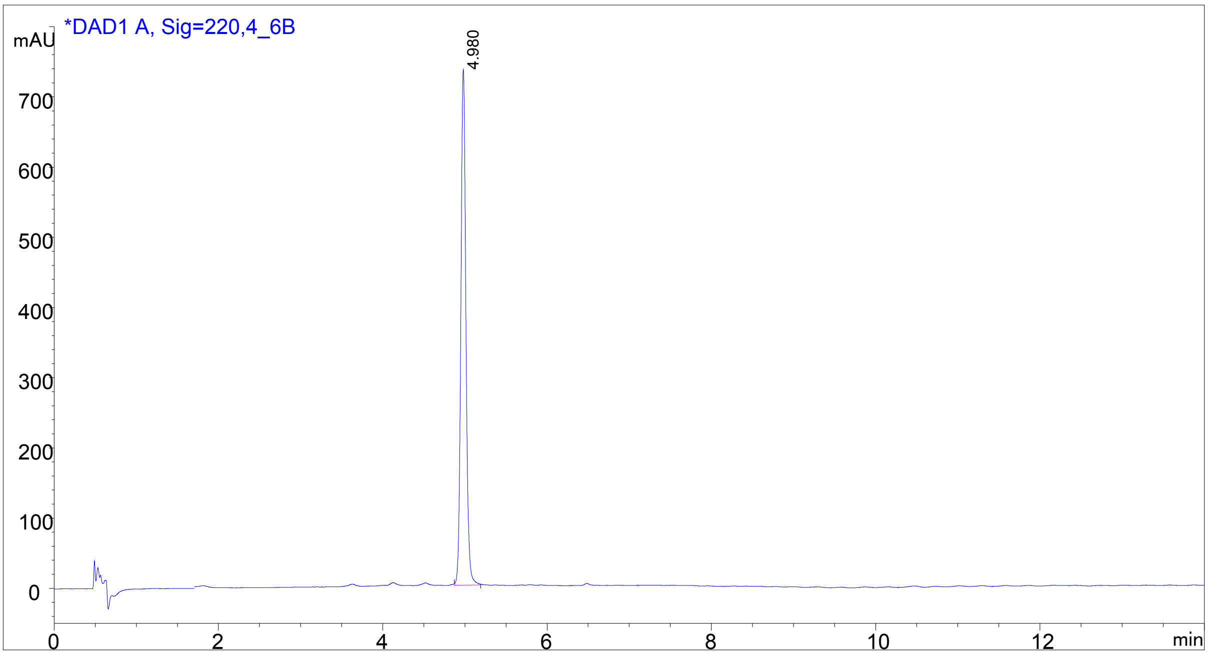

Figure S11. Chromatographic profile of **6b** monitored at λ=220 nm.

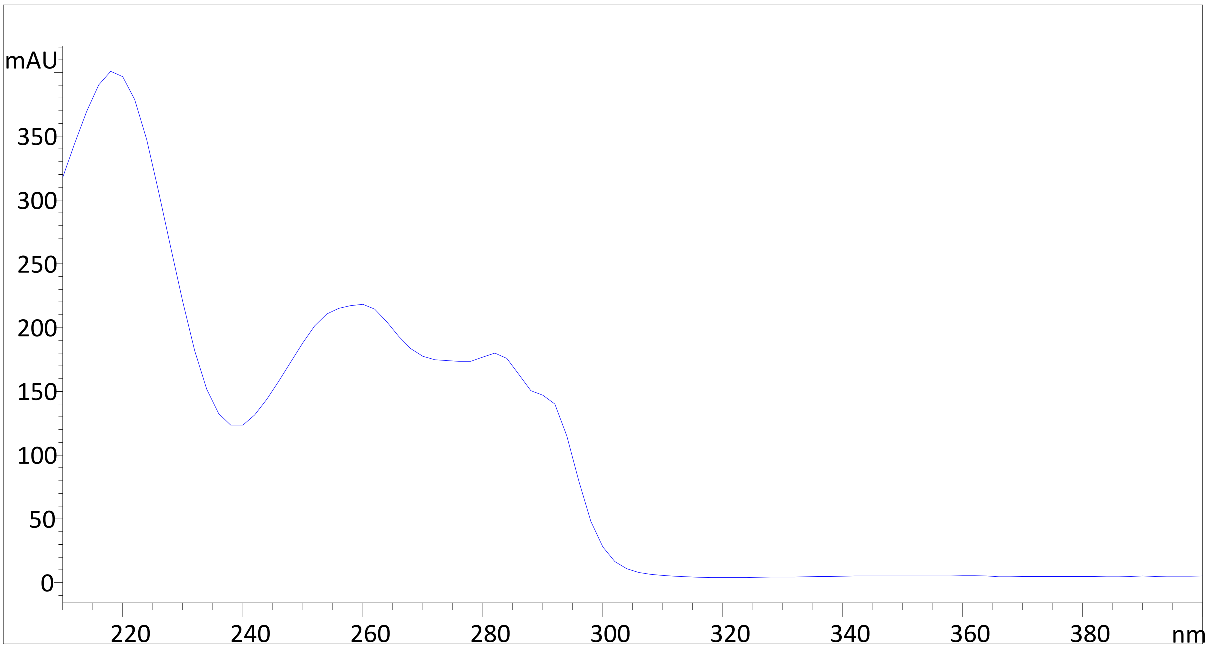

Figure S12. UV spectrum of the compound **6b**.

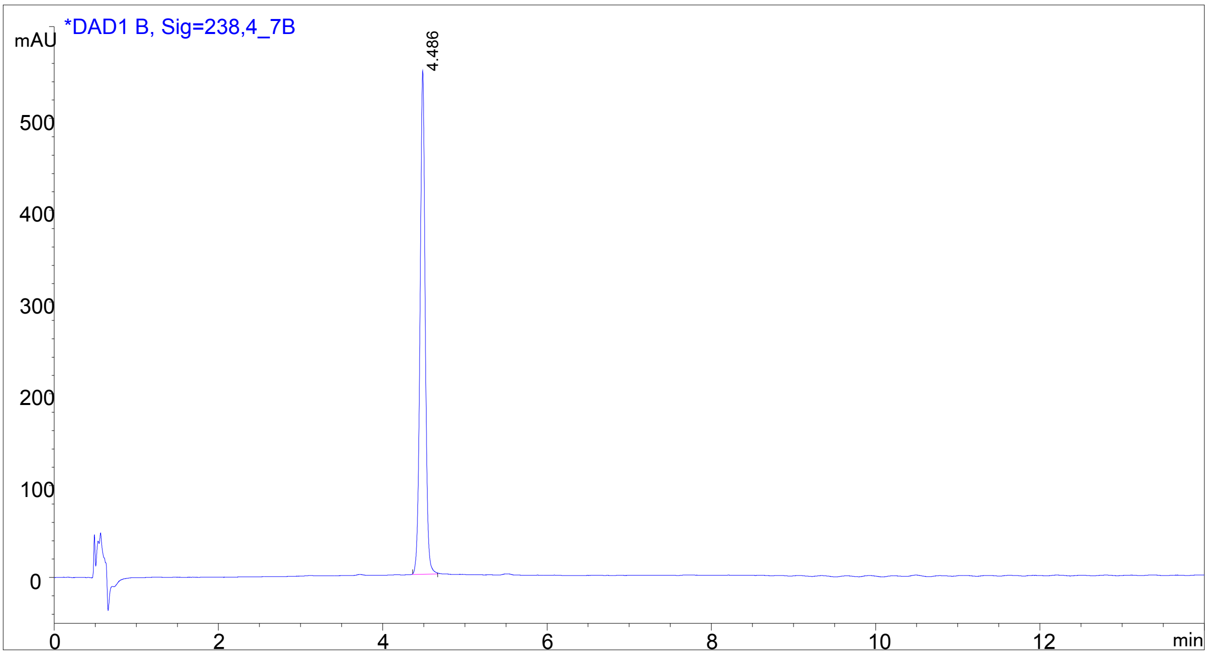

Figure S13. Chromatographic profile of **7b** monitored at λ=238 nm.

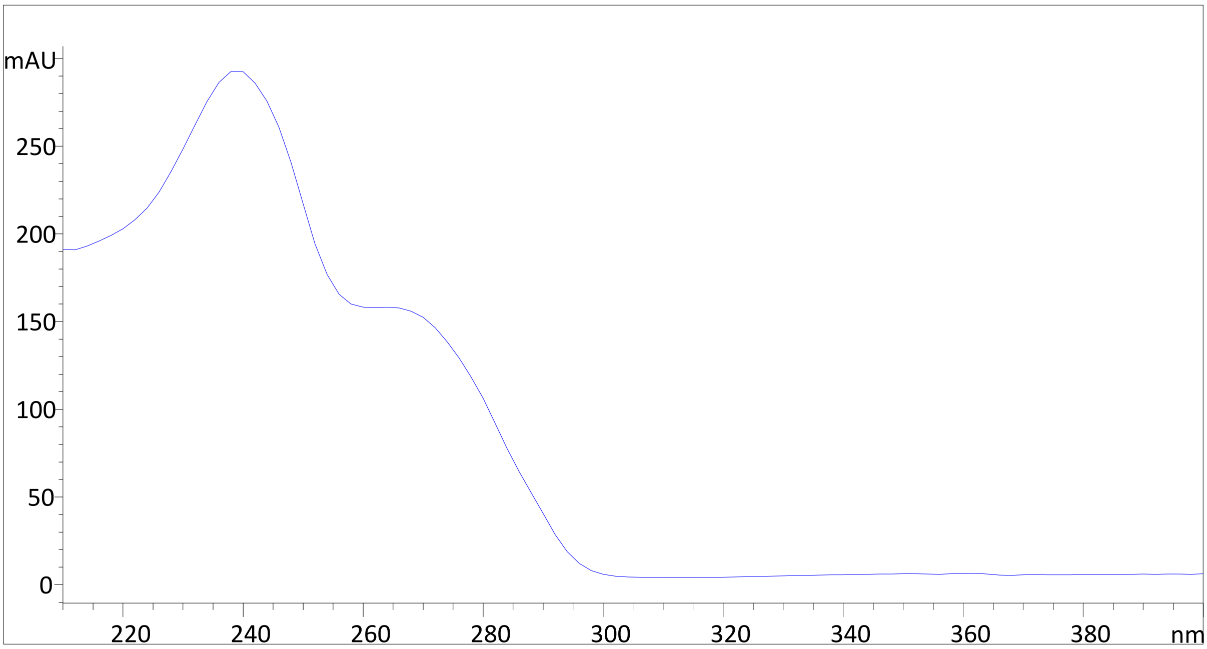

Figure S14. UV spectrum of the compound **7b**.

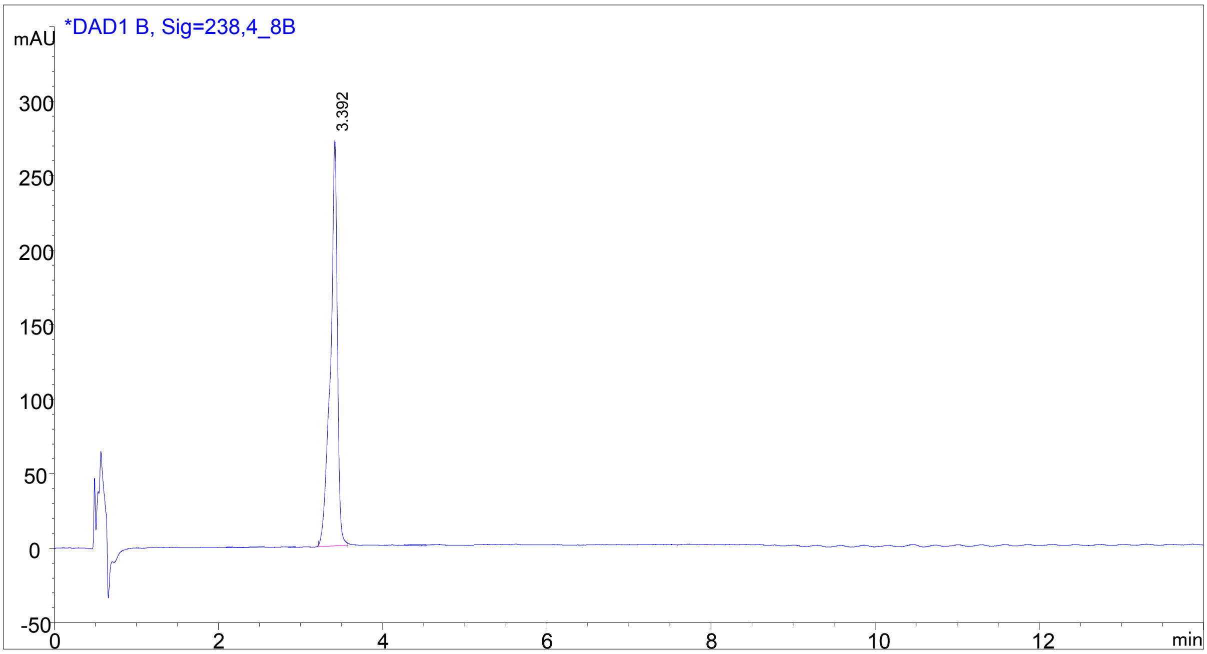

Figure S15. Chromatographic profile of **8b** monitored at λ=238 nm.

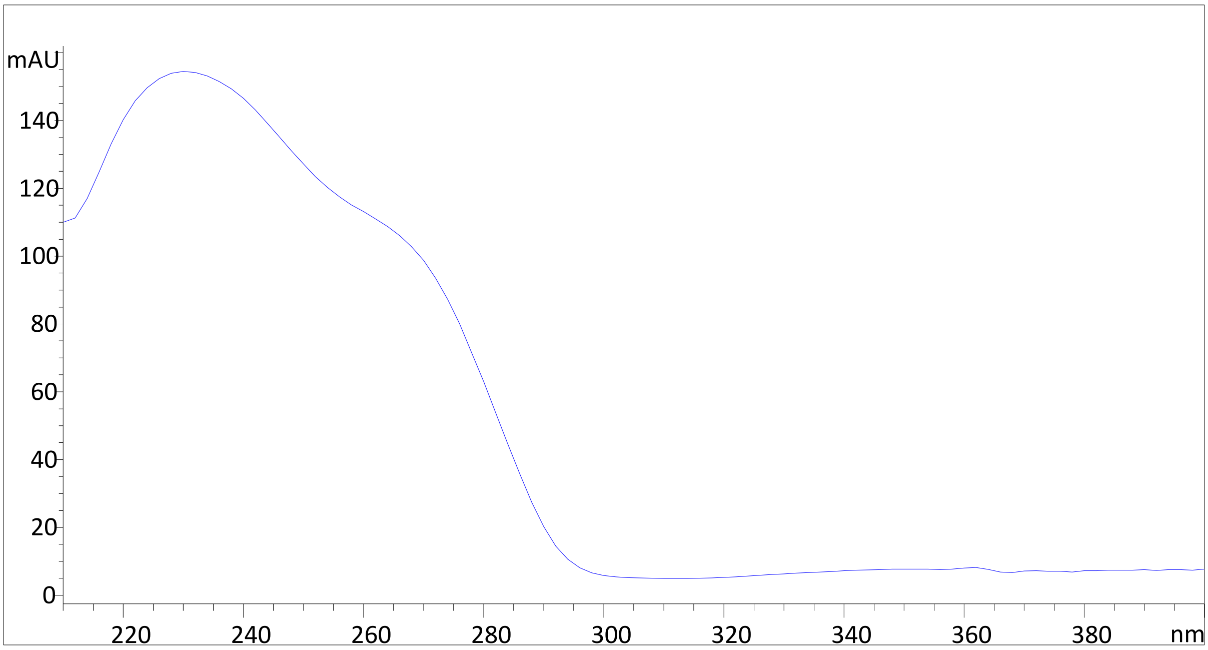

Figure S16. UV spectrum of the compound **8b**.

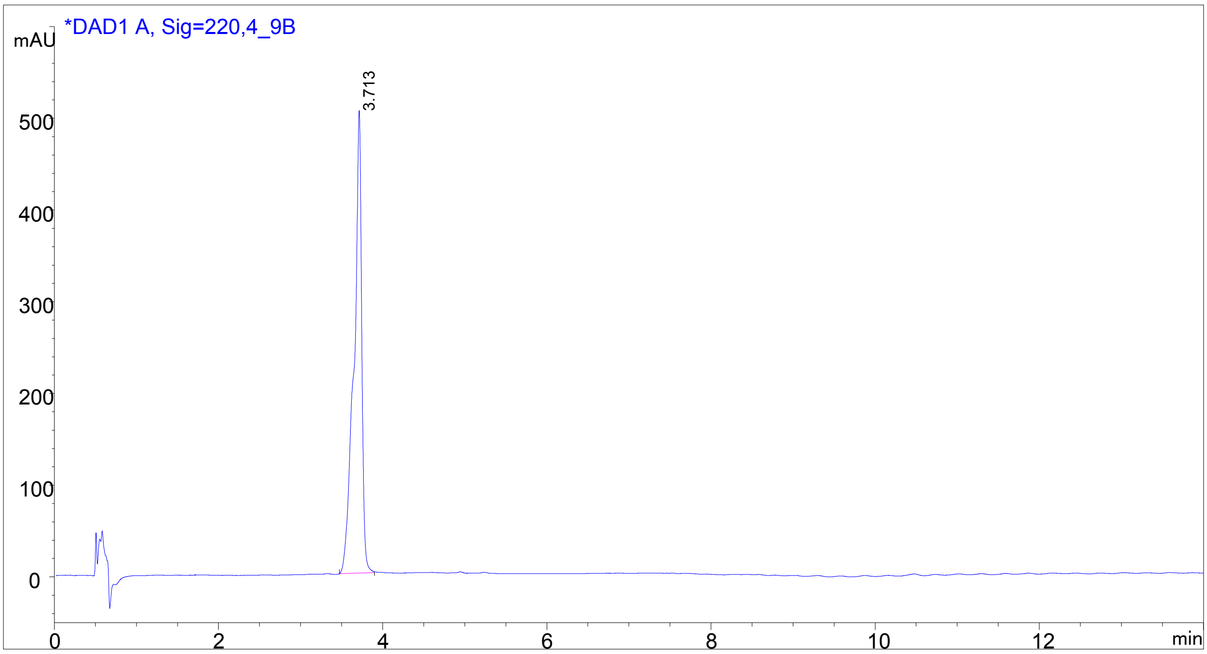

Figure S17. Chromatographic profile of **9b** monitored at λ=220 nm.

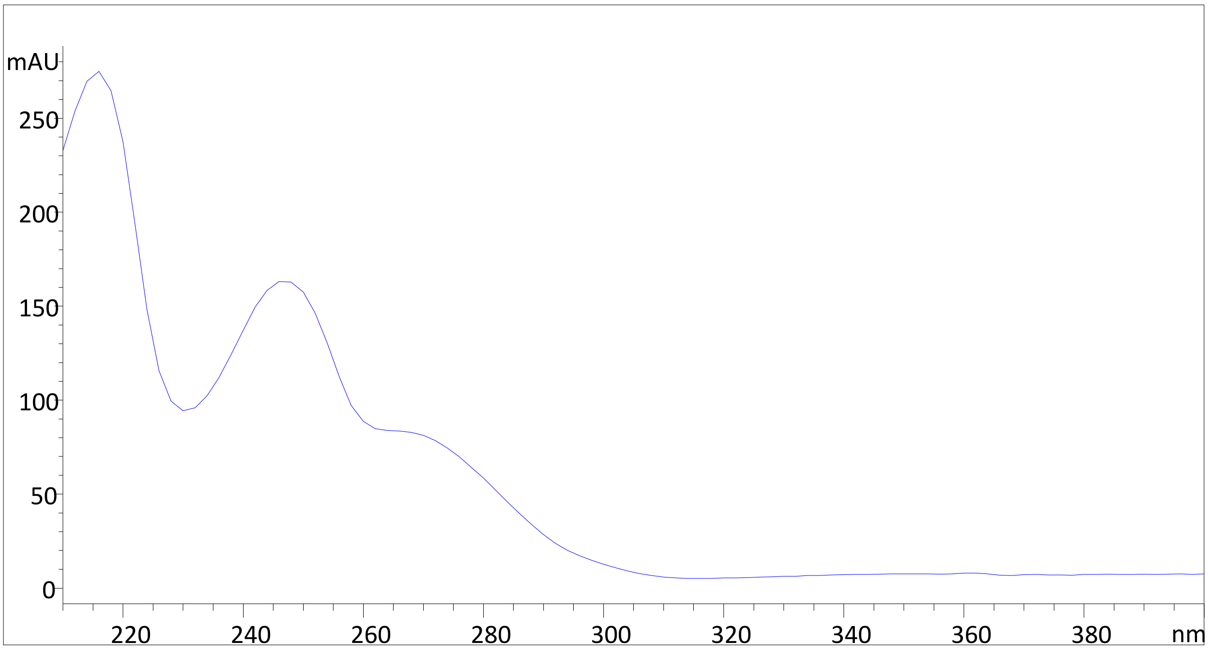

Figure S18. UV spectrum of the compound **9b**.

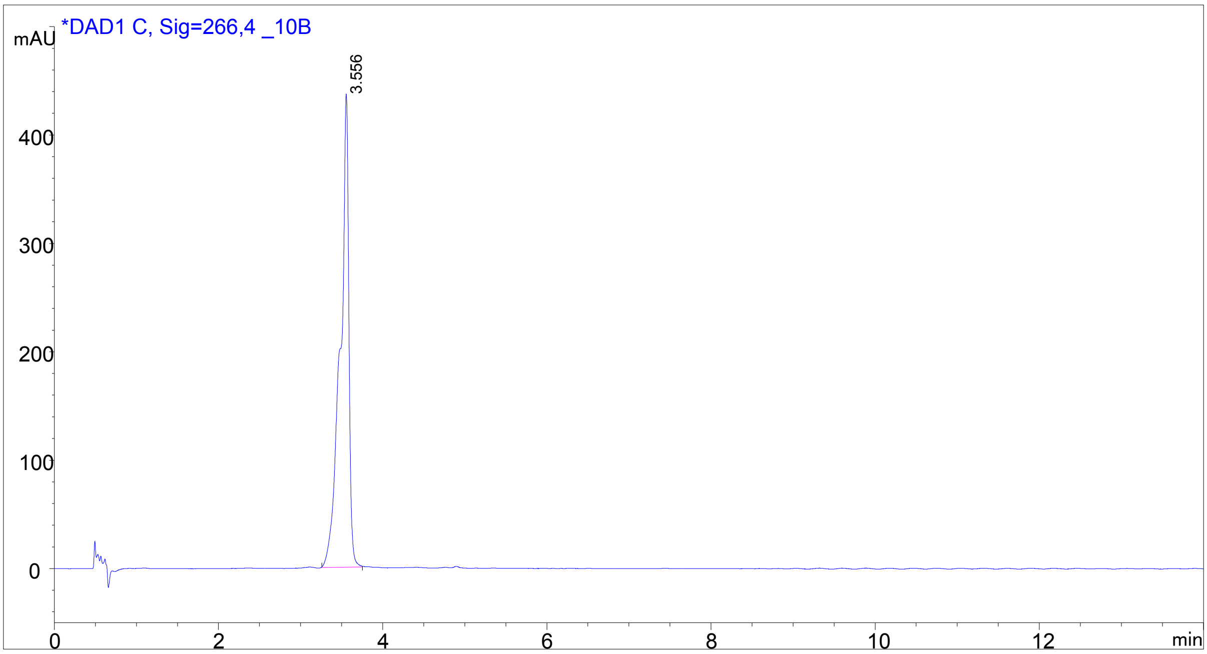

Figure S19. Chromatographic profile of **10b** monitored at λ=266 nm.

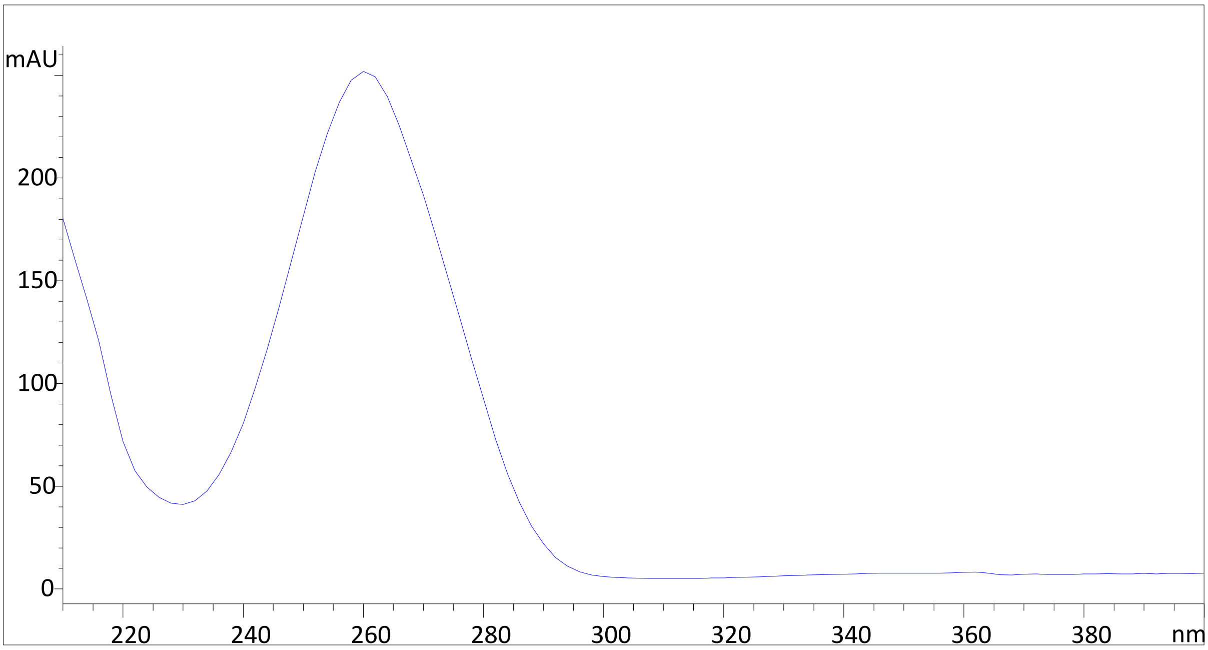

Figure S20. UV spectrum of the compound **10b**.

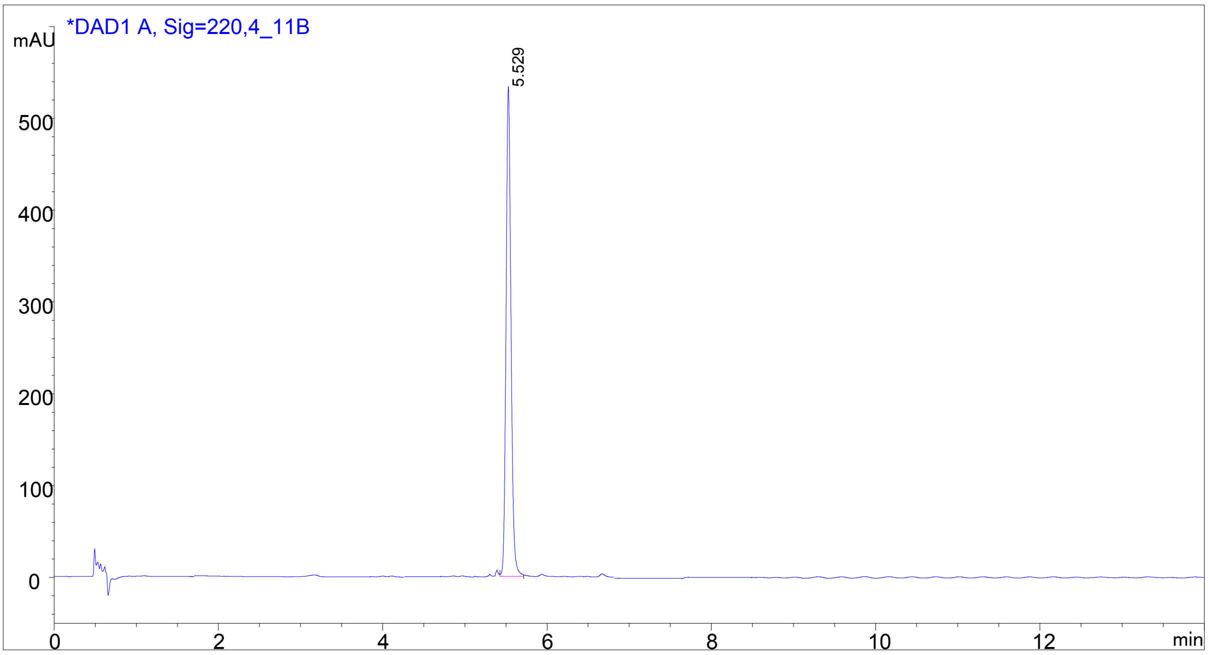

Figure S21. Chromatographic profile of **11b** monitored at λ=220 nm.

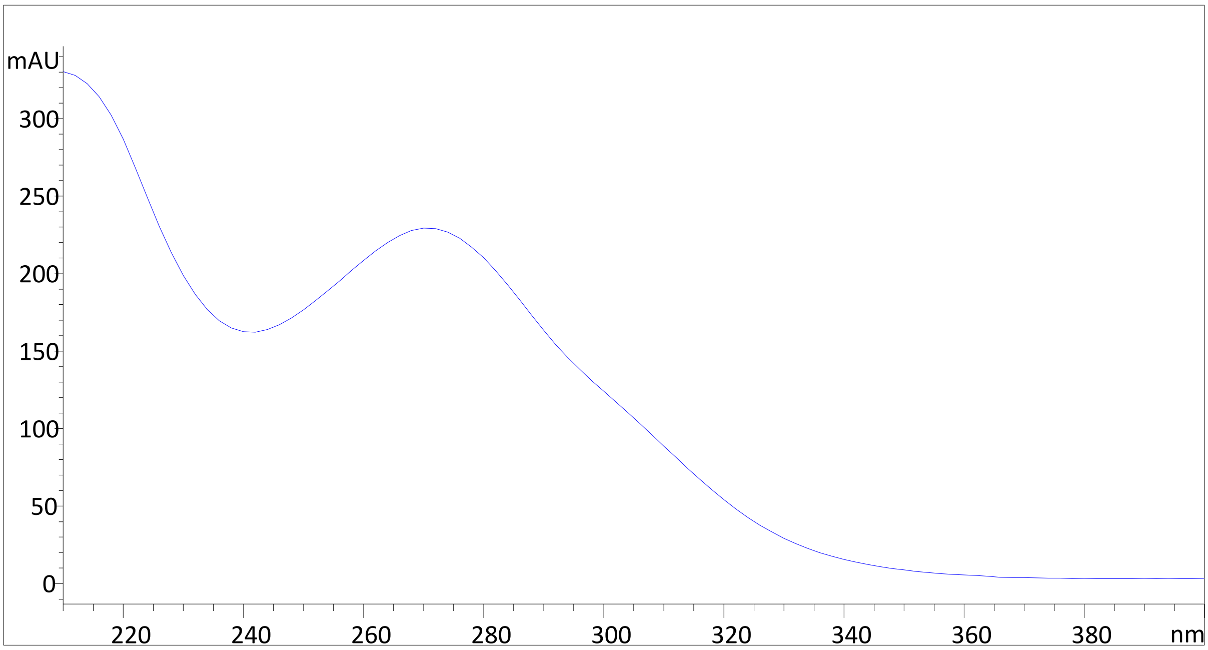

Figure S22. UV spectrum of the compound **11b**.

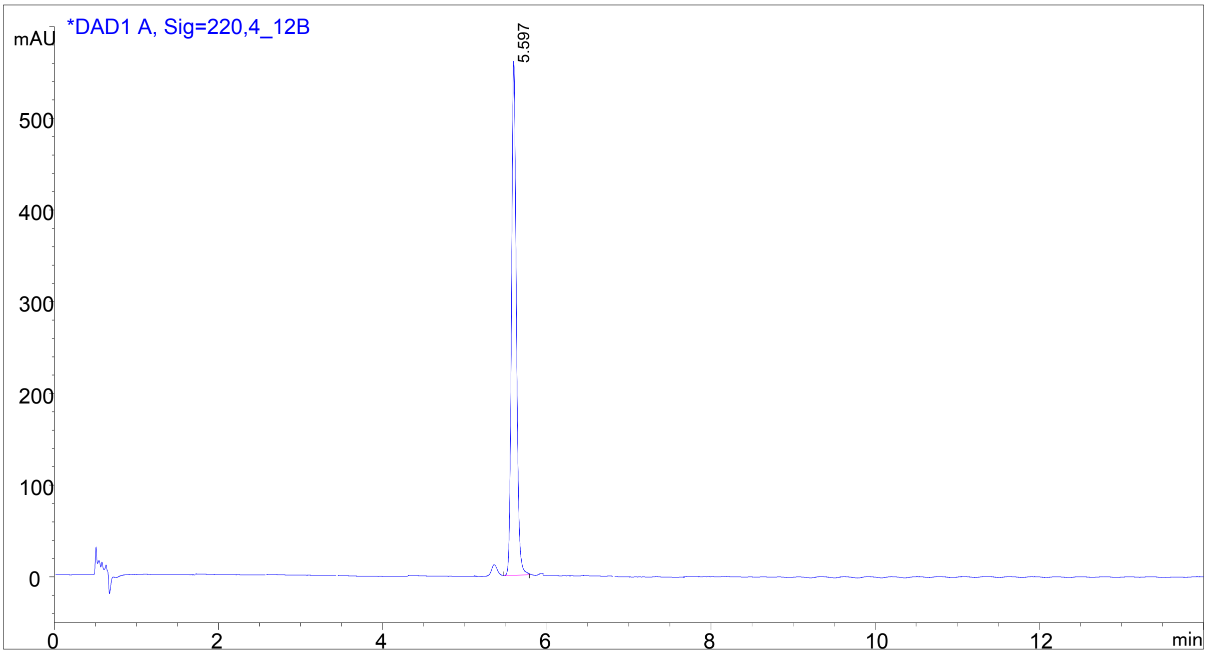

Figure S23. Chromatographic profile of **12b** monitored at λ=220 nm.

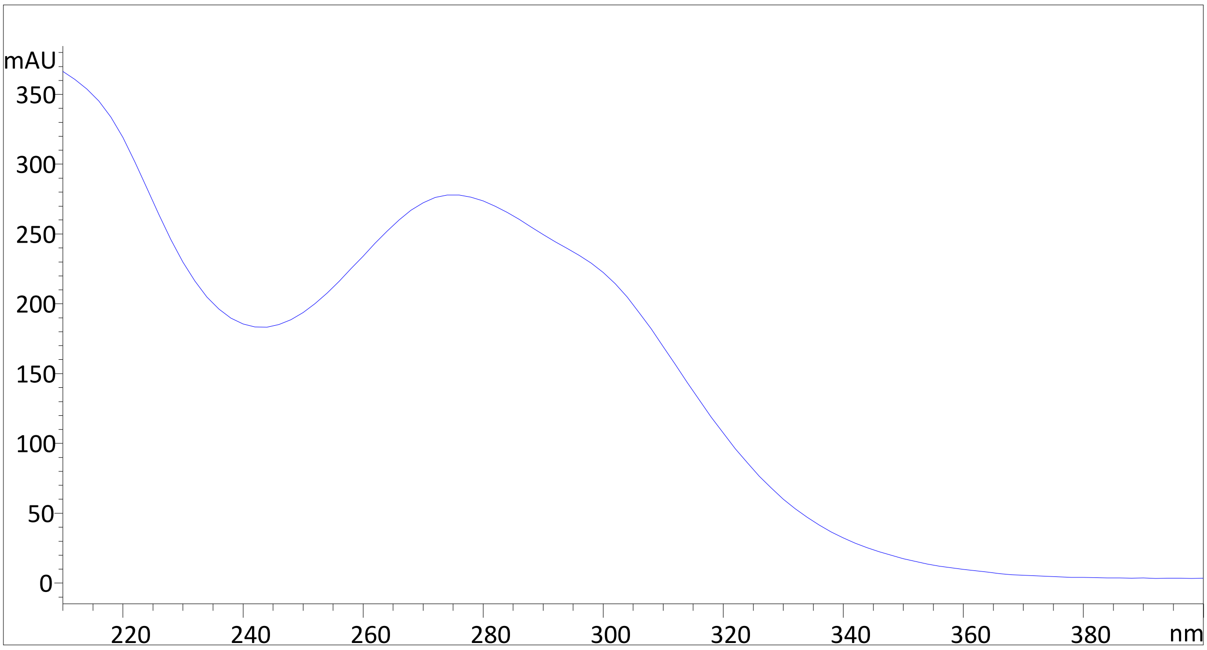

Figure S24. UV spectrum of the compound **12b**.

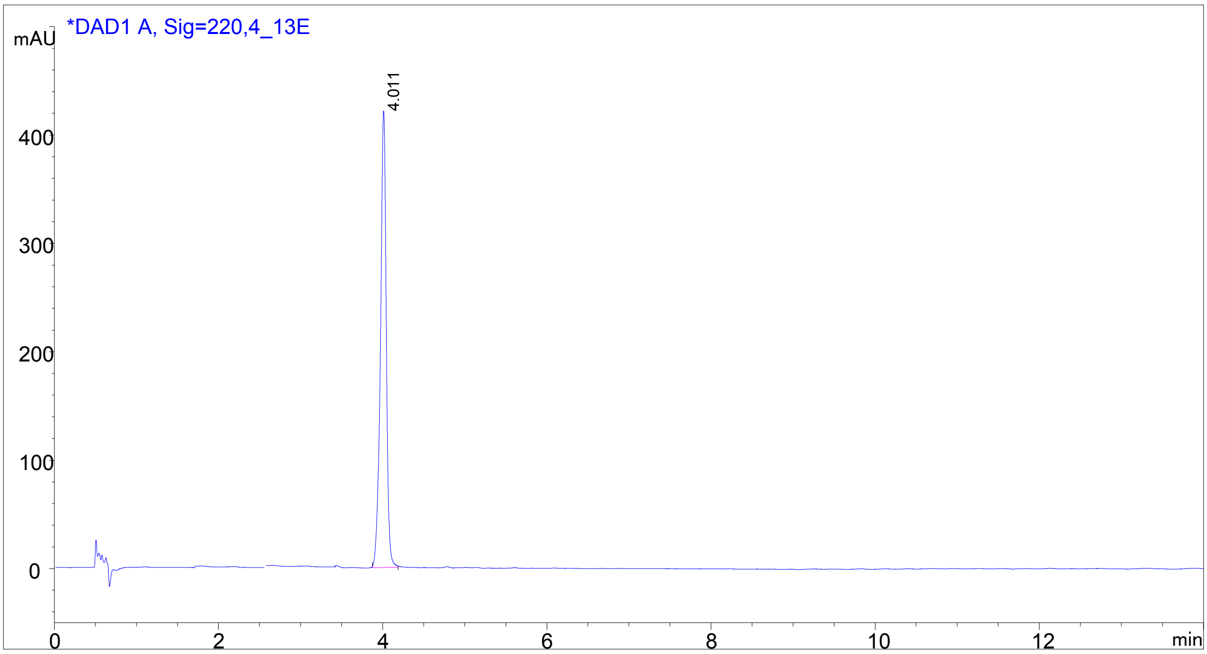

Figure S25. Chromatographic profile of **13e** monitored at λ=220 nm.

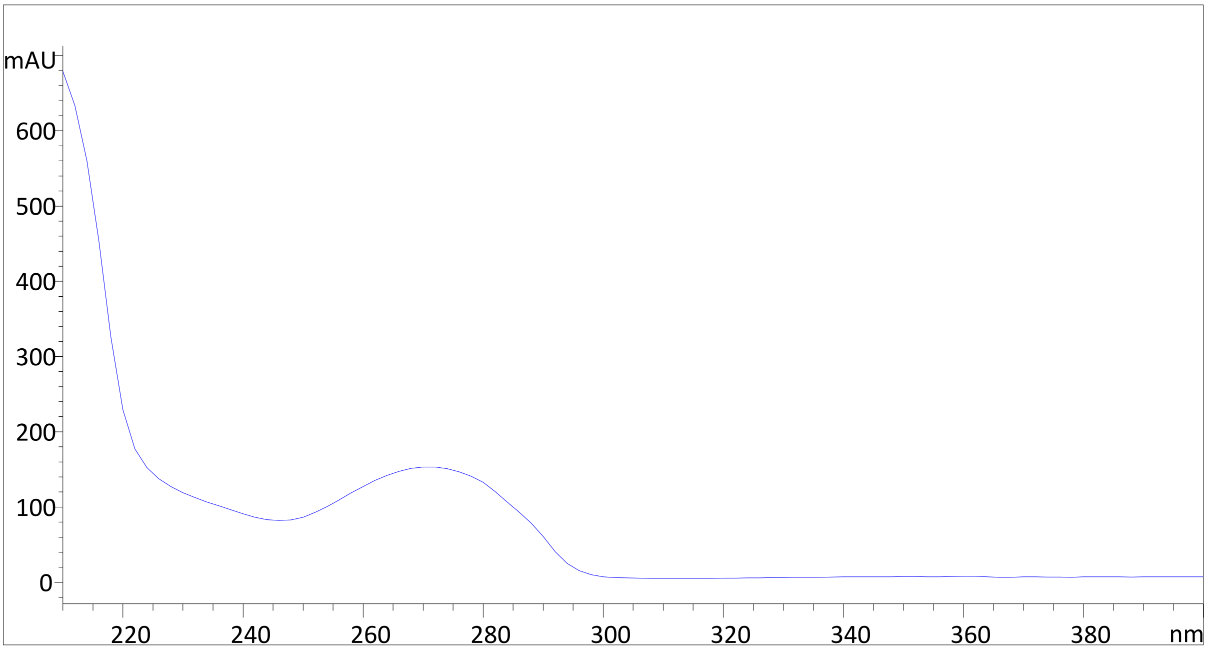

Figure S26. UV spectrum of the compound **13e**.

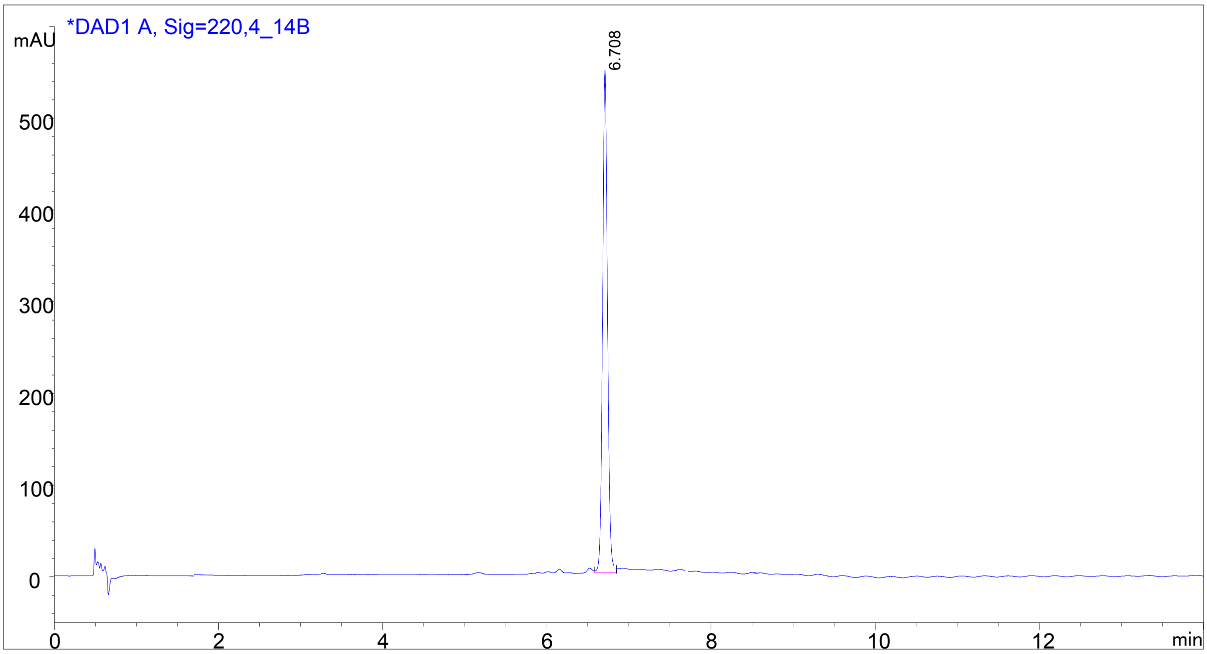

Figure S27. Chromatographic profile of **14b** monitored at λ=220 nm.

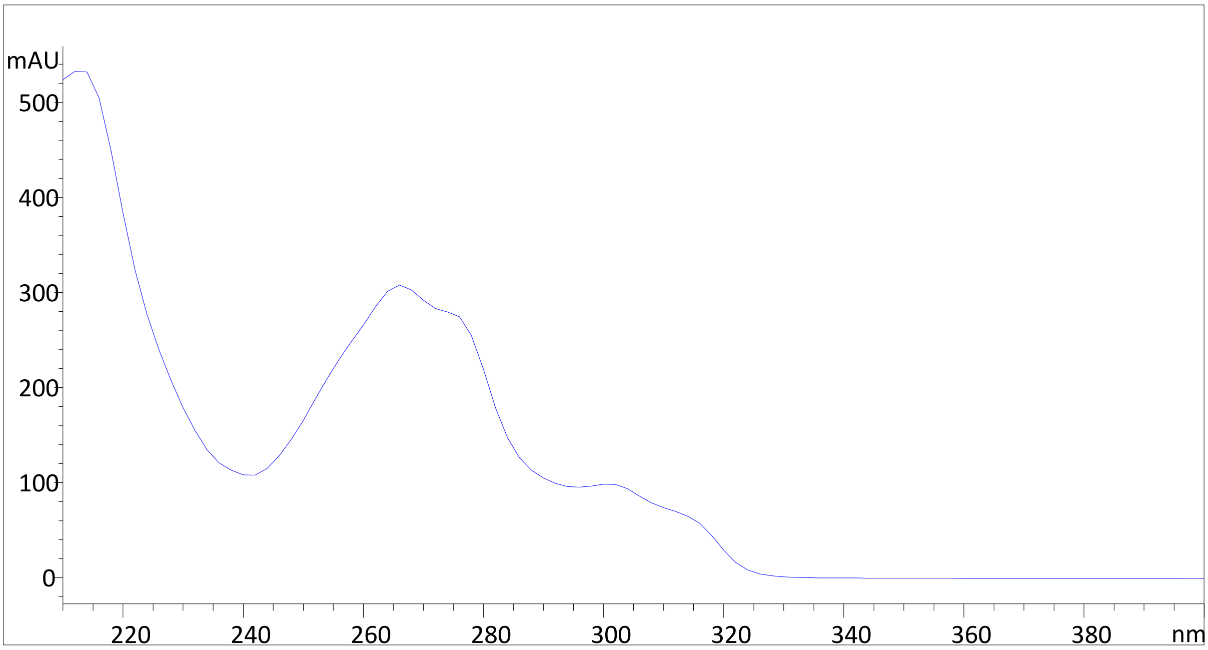

Figure S28. UV spectrum of the compound **14b**.

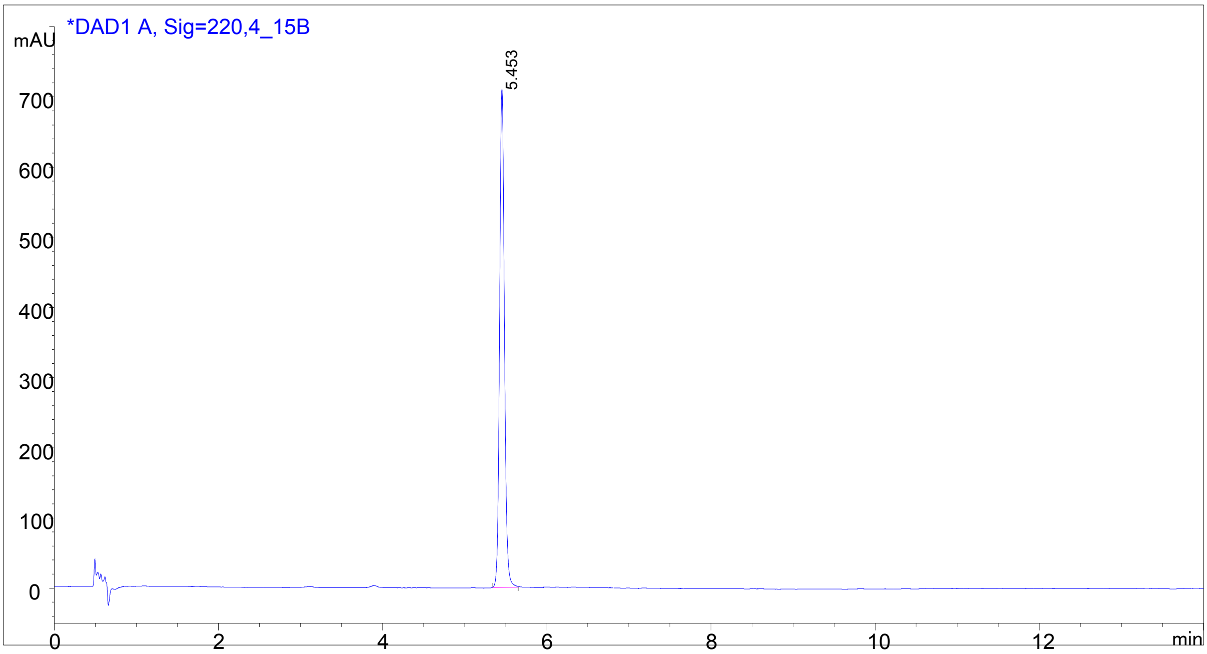

Figure S29. Chromatographic profile of **15b** monitored at λ=220 nm.

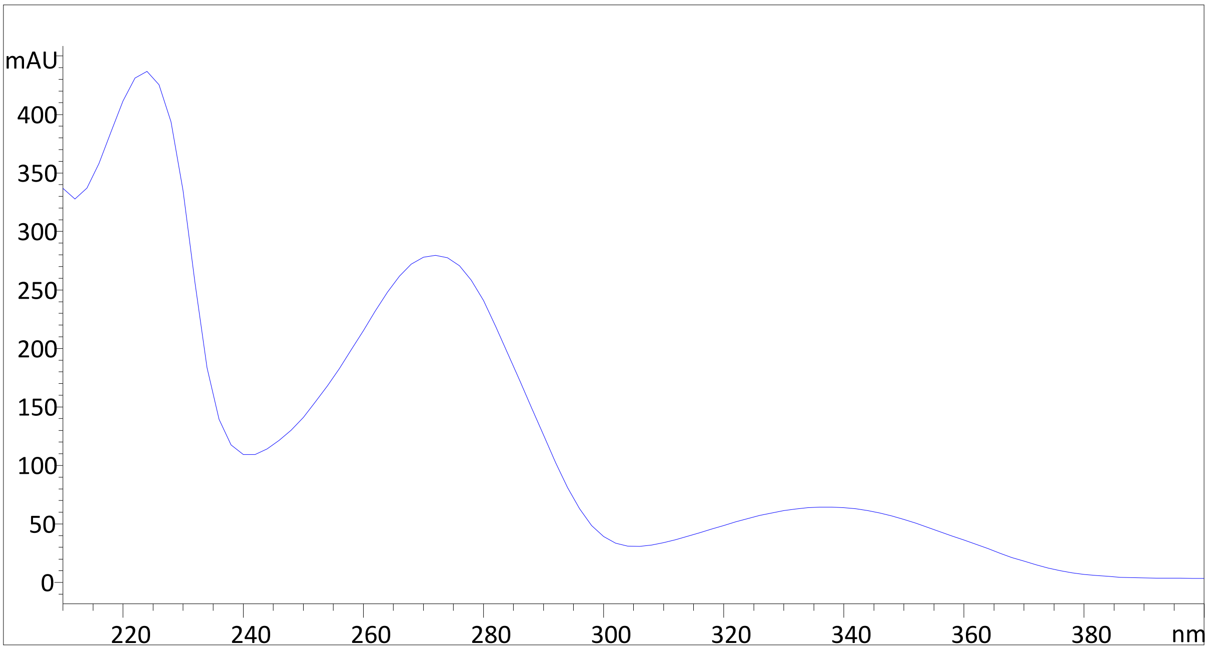

Figure S30. UV spectrum of the compound **15b**.

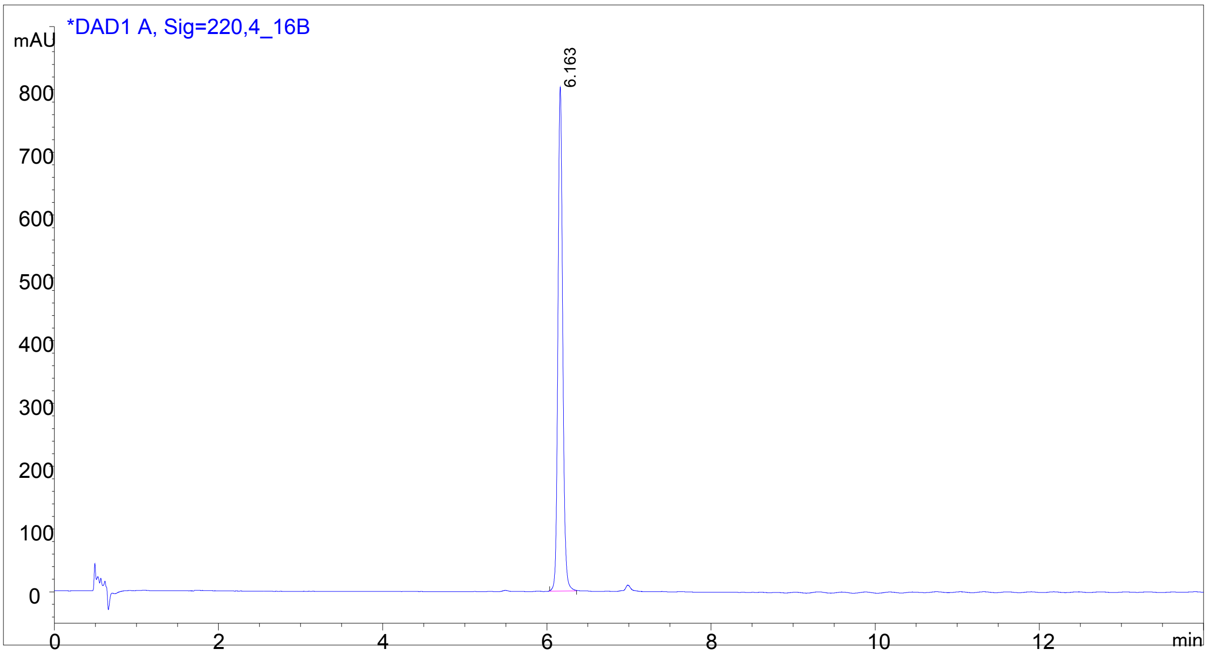

Figure S31. Chromatographic profile of **16b** monitored at λ=220 nm.

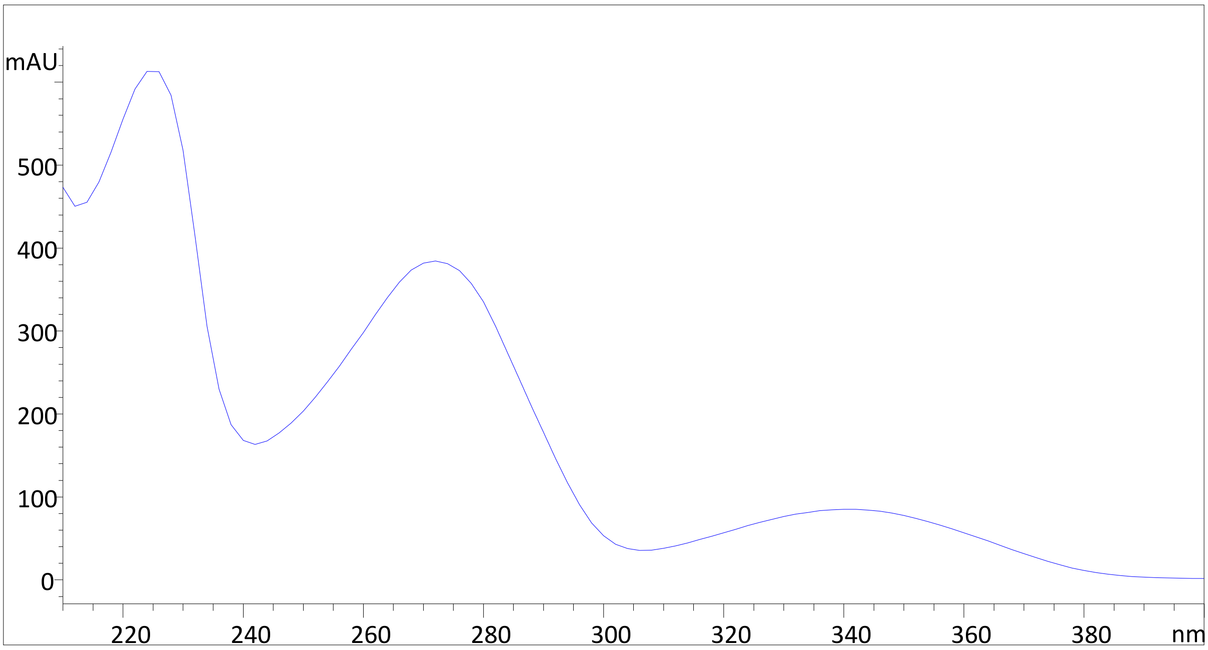

Figure S32. UV spectrum of the compound **16b**.

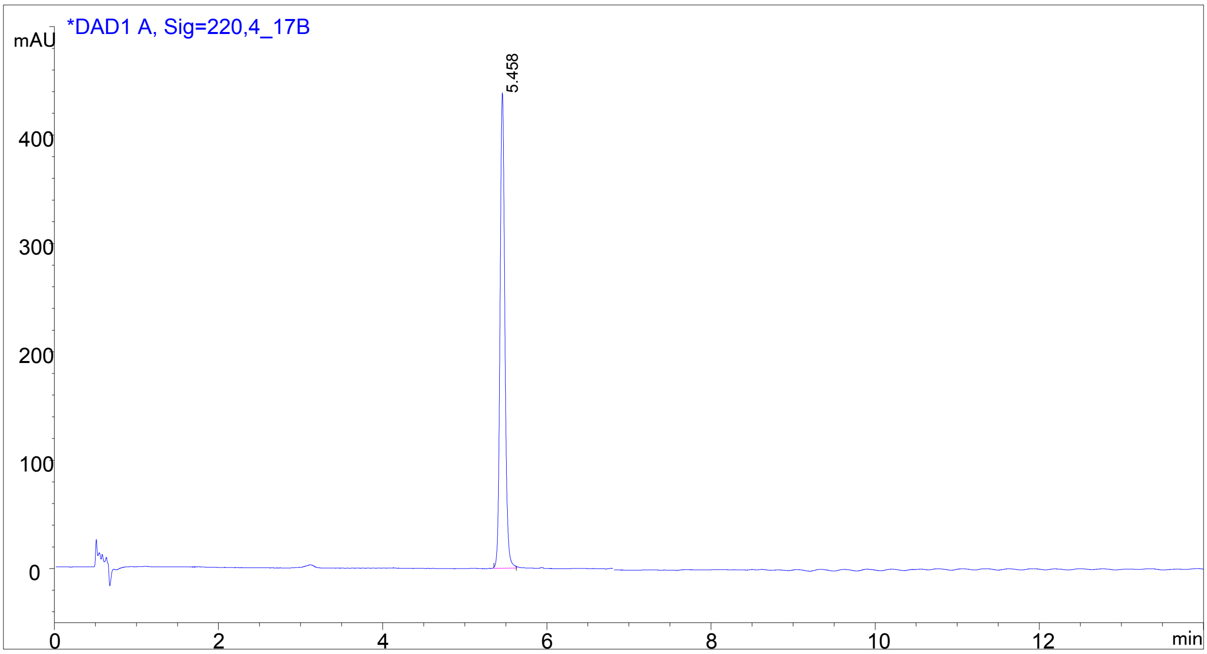

Figure S33. Chromatographic profile of **17b** monitored at λ=220 nm.

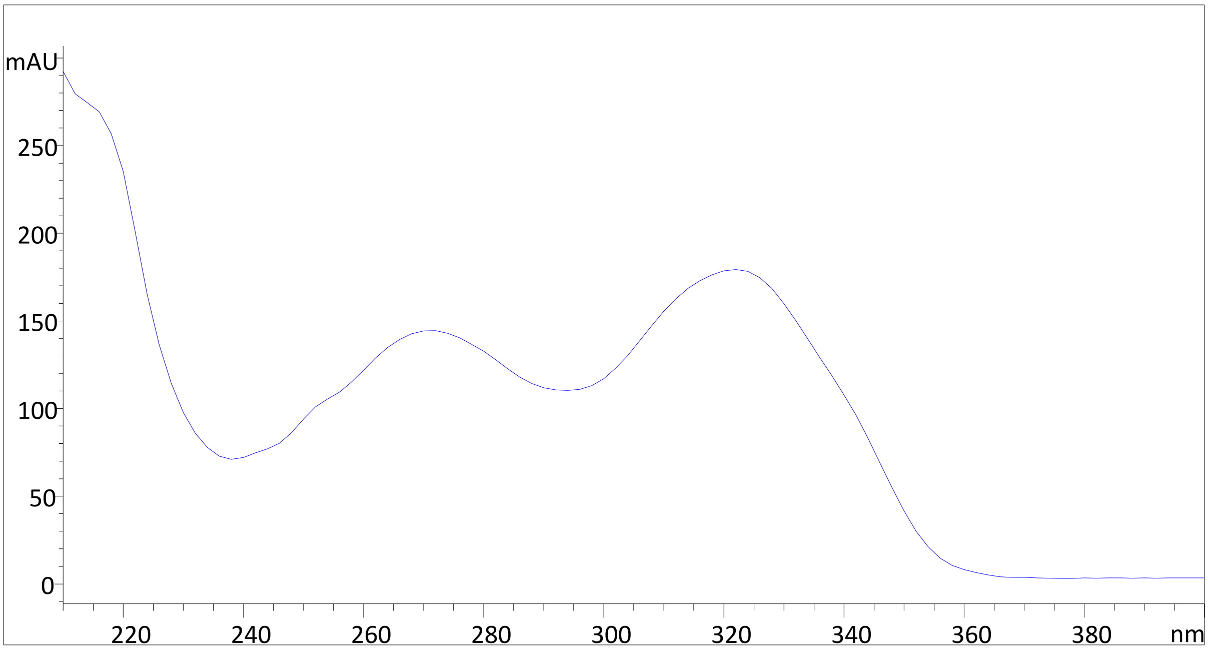

Figure S34. UV spectrum of the compound **17b**.

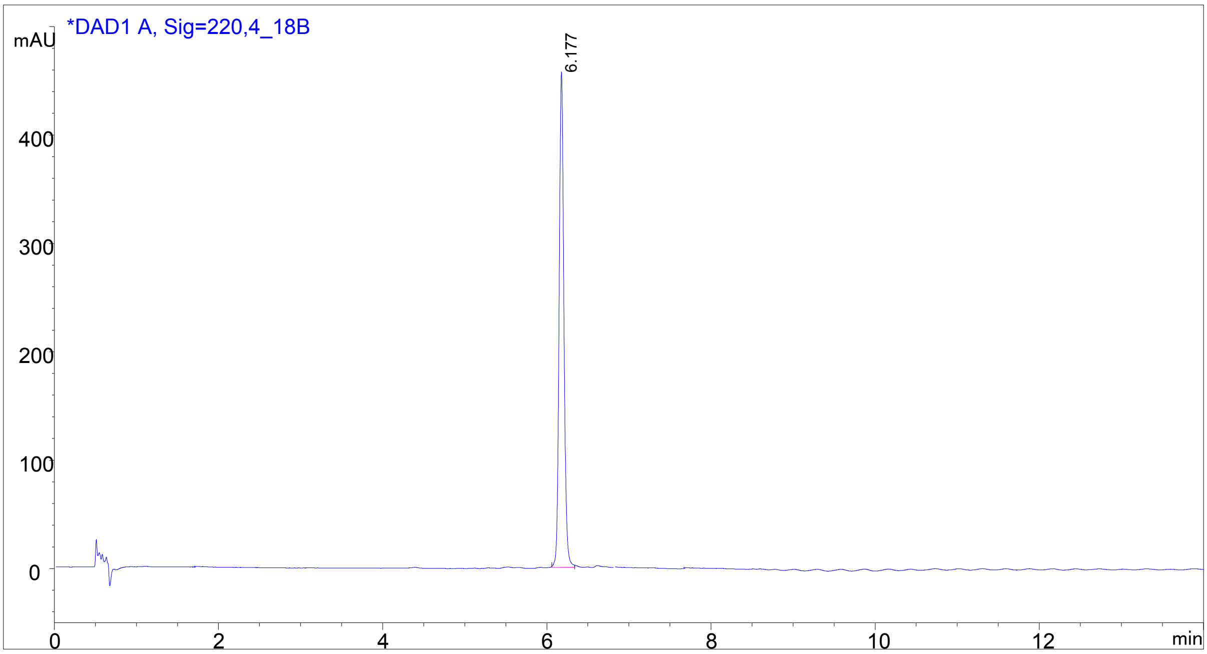

Figure S35. Chromatographic profile of **18b** monitored at λ=220 nm.

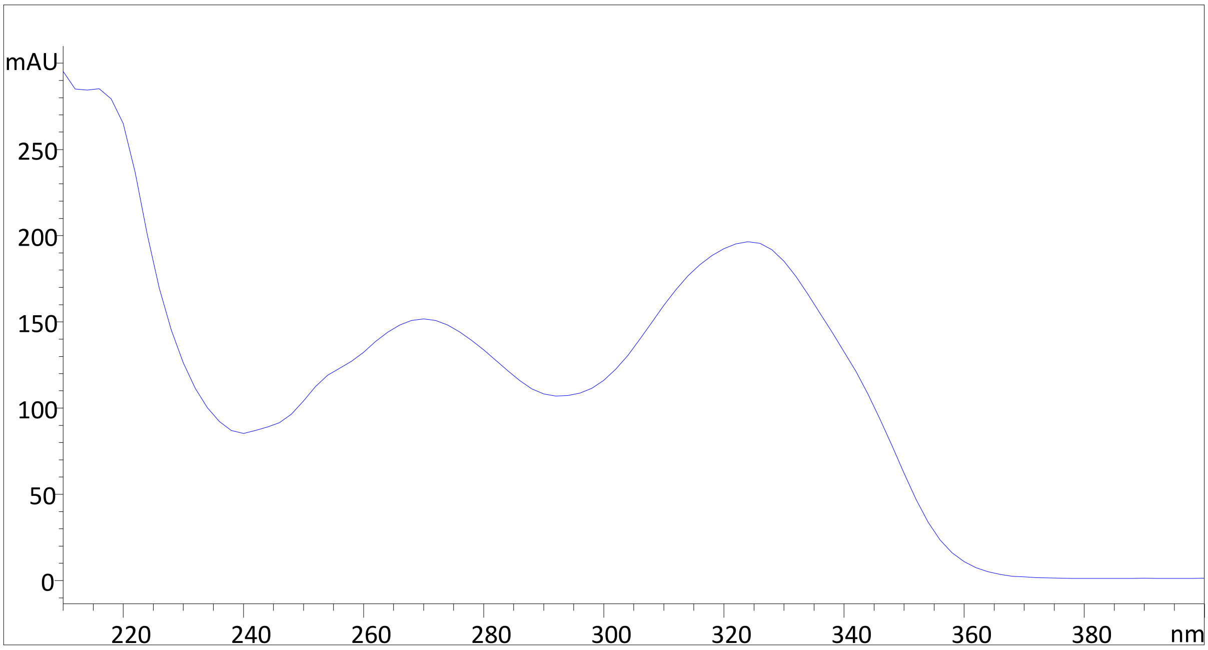

Figure S36. UV spectrum of the compound **18b**.

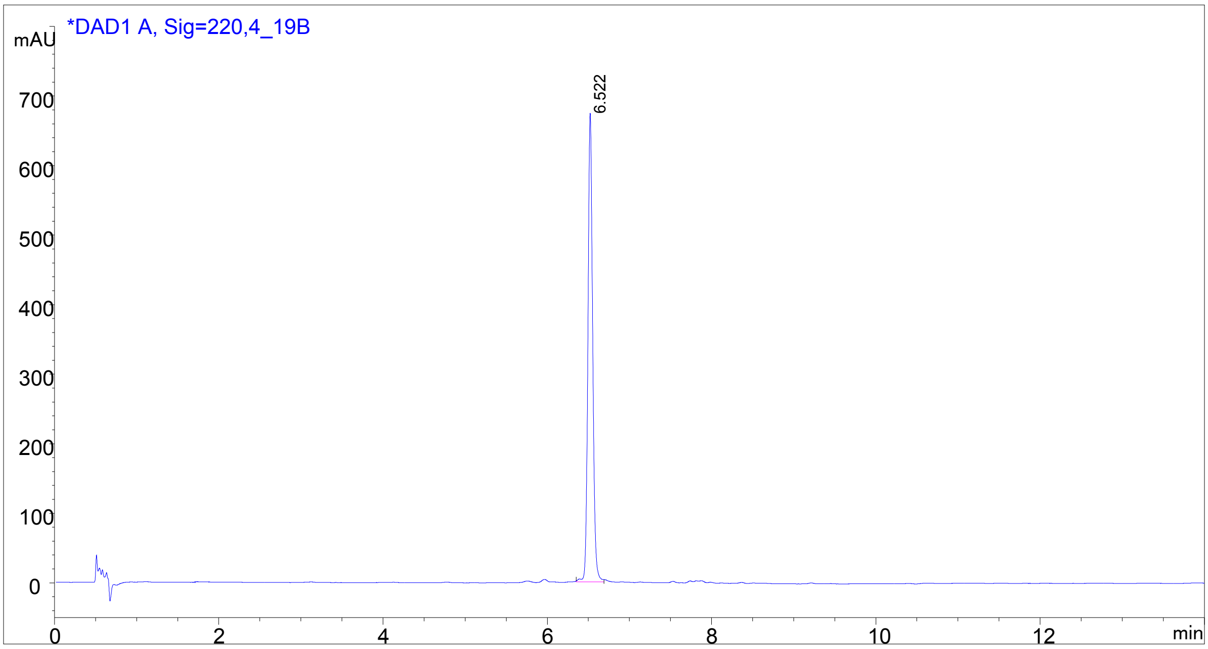

Figure S37. Chromatographic profile of **19b** monitored at λ=220 nm.

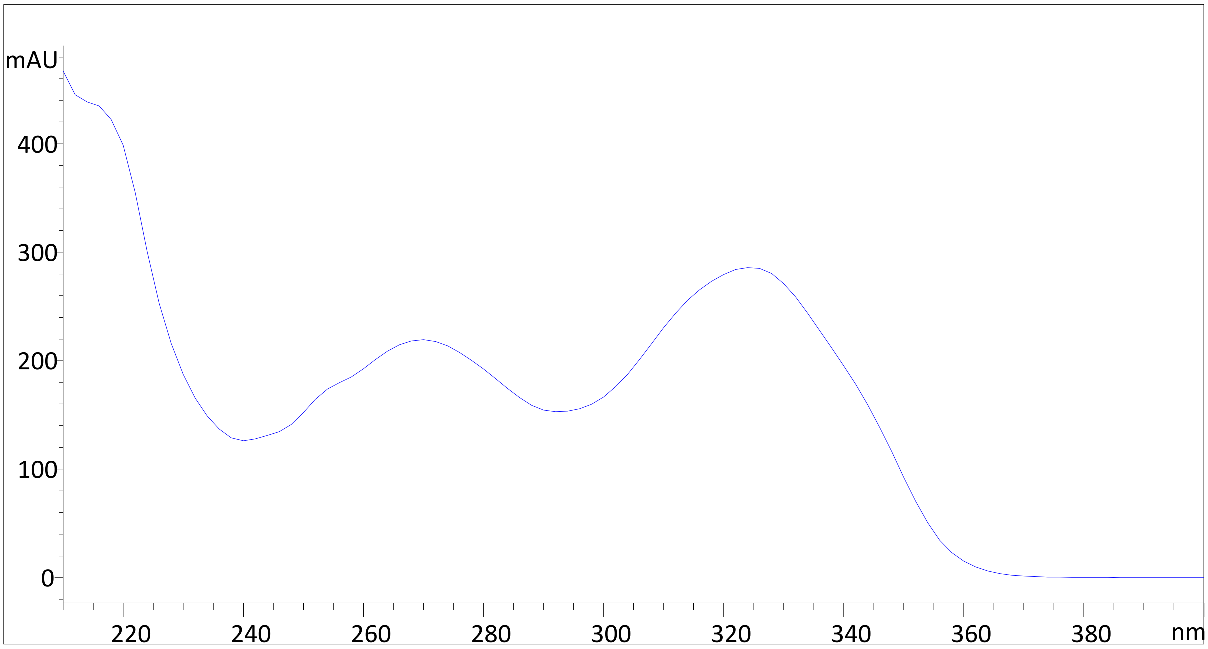

Figure S38. UV spectrum of the compound **19b**.

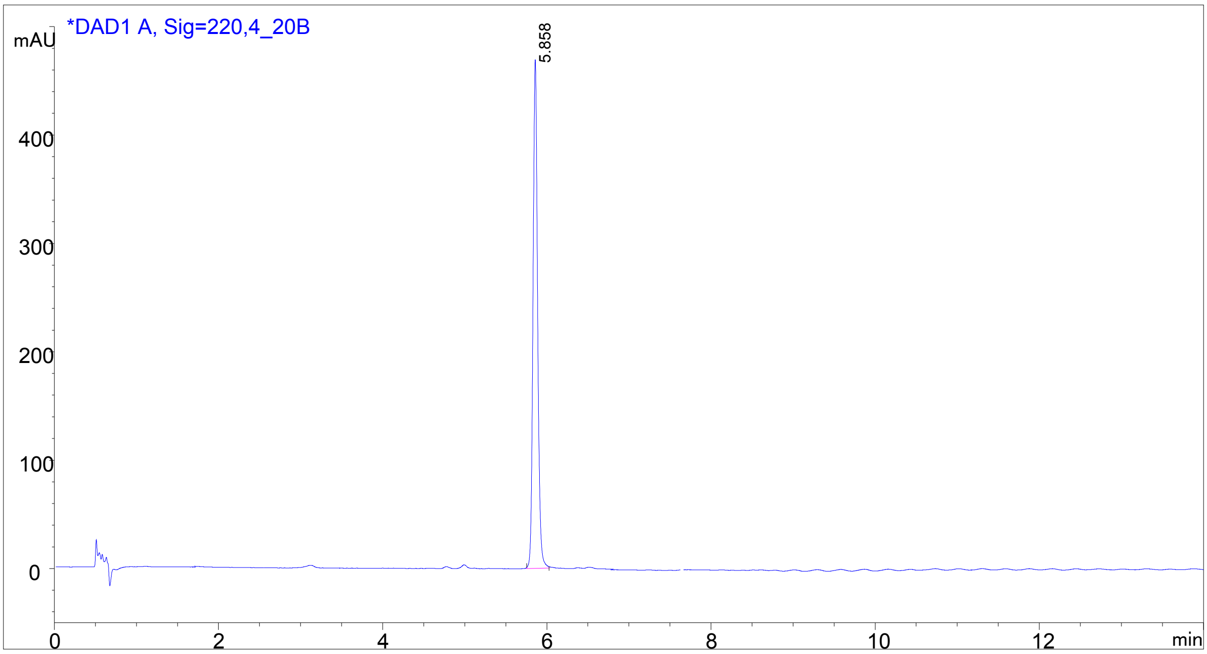

Figure S39. Chromatographic profile of **20b** monitored at λ=220 nm.

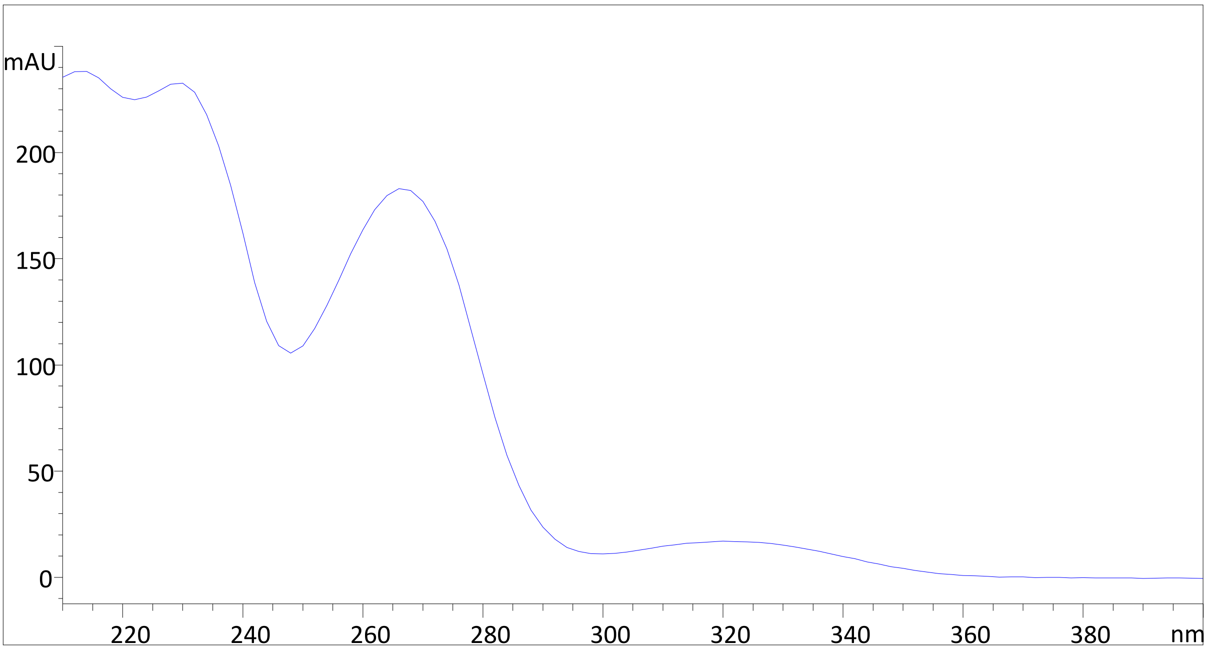

Figure S40. UV spectrum of the compound **20b**.
